# Adverse Effects of Excessive Zinc Intake in Infants and Children Aged 0–3 Years: A Systematic Review and Meta-Analysis

**DOI:** 10.1093/advances/nmac088

**Published:** 2022-09-02

**Authors:** Marena Ceballos-Rasgado, Nicola M Lowe, Simonette Mallard, Andrew Clegg, Victoria H Moran, Catherine Harris, Jason Montez, Maria Xipsiti

**Affiliations:** Centre for Global Development, University of Central Lancashire, Preston, Lancashire, United Kingdom; Centre for Global Development, University of Central Lancashire, Preston, Lancashire, United Kingdom; Independent contractor, Dunedin, New Zealand; Synthesis, Economic Evaluation and Decision Science (SEEDS) Group, Applied Health Research Hub, University of Central Lancashire, Preston, Lancashire, United Kingdom; Centre for Global Development, University of Central Lancashire, Preston, Lancashire, United Kingdom; Synthesis, Economic Evaluation and Decision Science (SEEDS) Group, Applied Health Research Hub, University of Central Lancashire, Preston, Lancashire, United Kingdom; Department of Nutrition and Food Safety, World Health Organization, Geneva, Switzerland; Food and Nutrition Division, Food and Agriculture Organization of the United Nations, Rome, Italy

**Keywords:** zinc, dietary requirements, children, upper limits, systematic review, meta-analysis

## Abstract

Zinc supplementation reduces morbidity, but evidence suggests that excessive intakes can have negative health consequences. Current guidelines of upper limits (ULs) of zinc intake for young children are extrapolated from adult data. This systematic review (PROSPERO; registration no. CRD42020215187) aimed to determine the levels of zinc intake at which adverse effects are observed in young children. Studies reporting potential adverse effects of zinc intake in children aged 0–3 y were identified (from inception to August 2020) in MEDLINE, Embase, and the Cochrane Library, with no limits on study design. Adverse clinical and physical effects of zinc intake were synthesized narratively, and meta-analyses of biochemical outcomes were conducted. Random effects models were used to generate forest plots to examine the evidence by age category, dose, dose duration, chemical formula of zinc, and zinc compared with placebo. The Joanna Briggs Institute Critical Appraisal Checklist, Cochrane Risk of Bias 2, and Grading of Recommendations Assessment, Development, and Evaluation (GRADE) guideline were employed to assess risk of bias and to appraise the certainty of evidence. Fifty-eight studies assessed possible adverse effects of zinc doses ranging from 3 to 70 mg/d. Data from 39 studies contributed to meta-analyses. Zinc supplementation had an adverse effect on serum ferritin, plasma/serum copper concentration, serum transferrin receptor, hemoglobin, hematocrit, and the odds of anemia in ≥1 of the subgroups investigated. Lactulose:mannitol ratio was improved with zinc supplementation, and no significant effect was observed on C-reactive protein, erythrocyte superoxide dismutase, zinc protoporphyrin, blood cholesterol, and iron deficiency anemia. The certainty of the evidence, as assessed using GRADE, was very low to moderate. Although possible adverse effects of zinc supplementation were observed in some subgroups, it is unclear whether these findings are clinically important. The synthesized data can be used to undertake a dose–response analysis to update current guidelines of ULs of zinc intake for young children.

## Teaser

**Statement of Significance**: This systematic review and meta-analysis was commissioned by the FAO-WHO, and aimed to synthesize the available evidence to answer clearly defined questions posed by the FAO-WHO expert group, regarding zinc intake in relation to needs for growth (tissue composition), excretion, absorption, and factors affecting bioavailability in children aged 0–3 y. Findings of this review can be a valuable resource for future national and international organizations looking to update or adapt dietary guidelines for infants and children.

## Introduction

The upper limit (UL) of a nutrient's intake has been defined as the maximum intake from food, water, and supplements that is unlikely to pose risk of adverse health effects to most individuals in the general population (1). This information is particularly valuable when designing large-scale supplementation or fortification programs to ensure that the resulting nutrient intake does not exceed a value that is considered safe for human health. ULs are determined through a risk assessment process that assesses the probability of the occurrence of an adverse health effect from an excess exposure to the nutrient (2). This process requires the collection of information of known or potential adverse effects attributed to the nutrient, followed by a dose–response analysis to determine the relation between the dose of the nutrient and adverse effect on key outcome measures (3). For most nutrients no adverse effects are anticipated when they are consumed as foods because their absorption and/or excretion are regulated through homeostatic mechanisms (1). This is the case for zinc, where absorption and excretion are adjusted over a wide range of dietary intakes (4). In addition, zinc is not stored in body tissues, thus the potential for zinc to reach toxic concentrations is limited. However, if zinc is ingested in excessive amounts or in smaller amounts but on a chronic basis through supplementation, it is associated with deleterious alterations in iron, copper lipoprotein, and cholesterol metabolism (3), and adverse physiological effects including nausea, vomiting, and general gastrointestinal disturbances (3, 5).

The current FAO-WHO values for zinc ULs are 35–50 mg/d (690 mmol/d) for adults, and 23–28 mg/d (350–430 mmol/d) for children, depending on their age (1). In setting these ULs, a dose–response analysis for children was not possible due to a lack of data, therefore the ULs for children in various age categories were extrapolated from adult data based on basal metabolic rate (1, 3). An alternative strategy was adopted when considering zinc ULs by expert groups convened by the Institute of Medicine (IOM) (6) and International Zinc Nutrition Consultative Group (IZiNCG) (7). Both groups used data from a small number of studies conducted in children relating to the impact of zinc intake on copper status. IZiNCG concluded that there were insufficient data to define ULs for children and instead published a “No Observed Adverse Effect Level” (NOAEL) value of 6–26 mg/d, depending on the age of the child. The IOM identified a NOAEL value and divided it by an uncertainty factor, which considered the length of exposure and the number of infants included in the 1 study (8). After obtaining values for young infants, the IOM adjusted the ULs for older infants and children on the basis of relative body weight to produce a recommendation of 4 mg/d for infants 0–6 mo, 5 mg/d for infants 7–12 mo, and 7 mg/d for children 1–3 y (6).

FAO-WHO has convened an expert group to update their vitamin and mineral requirements and ULs of intake for micronutrients in children aged 0–3 y (9), and commissioned this review to inform the work of updating the ULs for zinc in this age group. The aim of this review was to determine the levels of zinc intake at which adverse effects are observed in children aged 0–3 y.

## Methods

### Protocol and registration

This systematic review was registered with the international Prospective Register of Systematic Reviews (PROSPERO; registration no. CRD42020215187) and was conducted following the Preferred Reporting Items for Systematic Reviews and Meta-Analyses (PRISMA-2020) statement for reporting systematic reviews and meta-analyses (10).

### Eligibility criteria

Eligibility criteria are based on the Population, Intervention, Comparison, Outcomes, and Study (PICOS) elements. Criteria were identified through discussion with the expert group and are shown in **Table 1**.

**TABLE 1:** Eligibility criteria are based on the Population, Intervention, Comparison, Outcomes, and Study (PICOS) elements

Population(s)^1^	Children and adults who were generally healthy or who had symptoms related to excessive zinc intake
Interventions, exposures	Intake of zinc via supplements or foods (fortified and nonfortified)
	Studies where zinc intake was not given were excluded
	Studies where the effect of zinc intake could not be isolated were excluded
Comparator(s)	Higher zinc intake vs. lower or no zinc intake
Outcome	Adverse effects included impact on:
	Absorption/status of other minerals (e.g., copper, iron, etc.)
	Hemoglobin, ferritin
	Blood lipids
	Immune function
	Gastrointestinal function
	DNA breaks/damage
Study designs	Intervention studies assessing effects of zinc intake. Including but not limited to: RCTs, crossover RCTs, non-RCTs, pre-post studies
	Observational studies assessing effects of zinc intake. Including but not limited to: case-control, cohort studies, cross-sectional studies
	Case reports of excess zinc intake
	In vitro and animal studies were *not* included

1 Based on results of the scoping review and related discussions, the expert group concluded that the limited data available in children aged 0–36 mo would not be sufficient to identify ULs and therefore it was decided to expand the literature search to include studies in older children and adults. Data for older children and/or adults would be used (i.e., extrapolated) only if data obtained via the literature for children aged 0–36 mo were insufficient to identify ULs directly. RCT, randomized control trial, UL, upper limit.

### Search strategy, study selection, and data extraction

The search was carried out using MEDLINE (OVID), Embase (OVID), Cochrane Database of Systematic Reviews, and Cochrane Central Register of Controlled Trials (CENTRAL) (Cochrane Library) from date of inception to August 7, 2020. The searches had no date or language restriction. The initial search strategy is presented in **Supplemental File 1** and comprised terms related to zinc, adverse effects, toxicity, and outcomes known to be potentially affected by excess zinc. Because a preceding scoping review indicated a lack of studies investigating excess zinc intake in children aged 0–3 y, the search was not limited by age. However, for the purposes of this article, only studies that included children aged 0–3 y will be reported.

The search results were downloaded into Endnote software for automatic and manual deduplication and then exported into the Rayyan web app (11) where 1 reviewer (MC-R) screened for inclusion and exclusion by title and abstract. The articles were flagged when there was uncertainty, and discussions for their inclusion or exclusion took place with senior members of the review team (NML, VHM). Hand searches were conducted by examining the reference lists of the retrieved articles, and relevant systematic reviews. Articles potentially meeting inclusion criteria and those that remained uncertain were moved forward to the next stage, where 1 reviewer (SM) screened the full text. At each stage of screening, a randomly selected 10% of articles were cross-checked by a second member of the review team (NML, VHM, or MC-R). Any disagreement was resolved by discussion and changes made accordingly.

Due to the broad range of outcome measures reported by a small number of studies, the search strategy was adjusted to include studies that did not have terms related to toxicity and adverse effects in the title and abstract. The expanded search strategy is shown in Supplemental File 1. The purpose of this expanded search was to capture studies that measured relevant outcomes without the presupposition of toxicity, thus increasing the number of included studies, particularly at the lower zinc dose/exposure rates. The expanded search results were deduplicated against the original search and screened for inclusion and exclusion as described above (Supplemental File 1).

### Data extraction and synthesis

One reviewer (SM) extracted the data from the included articles into a specifically designed Excel form, and a randomly selected sample (10%) of extracted articles was cross-checked by a member of the review team (MC-R). Data extracted included bibliographic information, location, aim, study methods, population characteristics, type of exposures to zinc (the type of exposure, duration of exposure, amount of zinc exposed to), outcomes (adverse event or status measures), and background dietary zinc intake. If dietary zinc intake was not reported, data obtained from external studies conducted in areas and with demographics close to the study were used, with preference given to data nearest in age range to that of the study population. Where such data were not available, data from national surveys were obtained. Efforts were also taken to directly contact authors for dietary intake data, if available. For studies including only infants <6 mo of age at baseline (*n* = 2) (12, 13), estimated dietary zinc intake from exclusive breastfeeding was used, obtained from a systematic review on breast milk intake commissioned by FAO-WHO to inform the work of updating requirements and ULs (14). For studies in which zinc intake was only assessed from complementary foods, breast milk zinc intake was added to the complementary food zinc intake. These age-matched values were taken from the FAO-WHO breast milk review, to provide a total dietary zinc intake estimate (14).

Studies were considered for meta-analyses if they included children aged 0–3 y and where the effect of zinc intake could be isolated (including a comparable arm without zinc). Where possible, forest plots were generated for the following: age category (0–90 d, 91–180 d, 6–12 mo, >12 mo), dose of zinc given (<5 mg/d, 5–10 mg/d, 10.1–20 mg/d, >20 mg/d, bolus), duration of intervention (0–3 mo, >3 mo), chemical formula of zinc (gluconate, sulfate, acetate, not stated, other), zinc compared with placebo, and high compared with low dose of zinc.

A narrative synthesis was undertaken incorporating information about the population characteristics, zinc exposure, and physical and clinical descriptive outcomes proposed by the FAO-WHO expert group (i.e., vomiting, regurgitation, nausea, constipation, abdominal pain, drowsiness, mouth irritation, taste aversion, diarrhea, and dysentery). Because there was no standard way of reporting these outcomes, they were not considered for meta-analyses.

### Statistical analysis

Meta-analyses were conducted using RevMan (Version 5.4; The Nordic Cochrane Centre, The Cochrane Collaboration 2014). Where outcomes were presented as continuous data, they were synthesized as weighted mean differences with 95% CIs using the generic inverse-variance method. Dichotomous data were pooled as ORs with 95% CIs through the Mantel–Haenszel method. Given the likelihood of variability among the studies, we estimated random-effects models. Heterogeneity was assessed through visual inspection of forest plots and through the χ^2^ and *I*^2^ statistic, with possible causes investigated through subgroup and sensitivity analyses. Publication bias was assessed using funnel plots for comparisons of ≥10 studies (15), with the causes of asymmetry judged in relation to nonreporting bias, methodological quality, heterogeneity, and artefactual reasons. Where appropriate, Grading of Recommendations, Assessment, Development, and Evaluation (GRADE) assessment was adjusted to reflect any bias identified.

### Assessment of risk of bias

Cochrane Risk of Bias 2 (RoB) was used to assess the quality of all randomized controlled trials (RCTs) (16). Four trials were classified as “quasi-experimental” rather than RCTs (8, 17–19) because details of the randomization process were not made explicit, although all had elements of random allocation and all were double blind. In 2 studies participants were allocated into groups based on successive age sequence (8, 18), and in 2 studies methods of allocation were not adequately described (17, 19). Therefore these studies were deemed acceptable for inclusion in meta-analyses by consensus and were assessed using Cochrane RoB. Two case studies (20, 21) were assessed using the Joanna Briggs Institute (JBI) Appraisal checklist (22). The GRADE (23) system was used to evaluate the certainty of evidence of all outcomes included in meta-analyses. Because all studies included in meta-analyses had been assessed using Cochrane RoB, the initial level of certainty for GRADE was high (24). Reasons for considering lowering the level of certainty included risk of bias, inconsistency, indirectness (such as the population having an underlying health condition), imprecision, and publication bias (25). Raising the level of certainty was not considered because this is generally used for observational studies only (24).

## Results

### Description of studies

**FIGURE 1:**
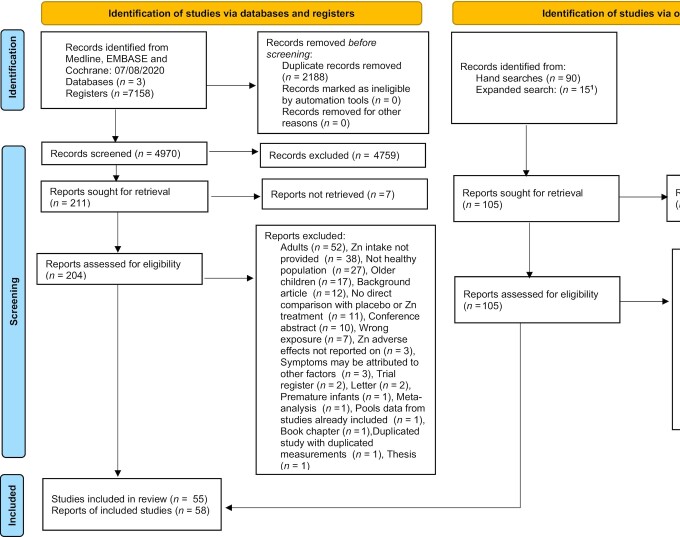
PRISMA-2020 flow diagram of the search procedure. ^1^See Supplemental File 2 for further information on how studies were selected. PRISMA, Preferred Reporting Items for Systematic Reviews and Meta-Analyses. (Note: Reports refers to ‘articles’ in the main text).

From the studies retrieved by the initial electronic search (*n* = 7158), hand searches (*n* = 90), and the expanded search (*n* = 15), 316 potentially relevant studies were assessed for inclusion when the full-text reports had been obtained. From these, 180 articles were excluded on a full-text basis and the reasons for exclusion are shown in the PRISMA diagram in **Figure 1**. A total of 136 articles were considered relevant across all population groups (*n* = 58 infants and children aged 0–3 y, *n* = 18 children aged >3 y, and *n* = 53 adults). Because the evidence available on infants and children aged 0–3 y was sufficient to avoid extrapolation from data on older children and adults, this article focuses only on 58 articles from 55 studies (8, 12, 13, 17–21, 26–75) that investigated possible adverse effects of excess zinc intake in infants and children aged 0–3 y. A complete list of biochemical outcome measures and physical and clinical descriptive outcomes included in these studies is given in **Table 2**. (Detailed information on study design can be found in Table 1 of **Supplemental File 2**.)

**TABLE 2:** Biochemical, physical, and clinical adverse effects of zinc intake in infants and children aged ≤3 y reported in the included studies^1^

Outcome reported	Number of studies^2^	References
*Biochemical outcome measures*		
Hemoglobin	34 (32)^3^	(8, 12, 13, 18, 19, 26, 28, 29, 31, 33–40, 43, 47, 48, 51, 53, 54, 58, 59, 62, 64, 65, 70–73) (66)^4^ (52)^5^
Hematocrit	6 (6)	(8, 19, 31, 51, 62, 65)
Serum ferritin	21 (20)	(12, 18, 19, 26, 28, 29, 31, 34, 35, 39, 40, 47, 48, 51, 59, 70–72, 74, 75) (52)^3^
Serum/soluble transferrin receptor	5 (5)	(12, 28, 36, 47, 74)
Iron deficiency	9 (9)	(12, 31, 34, 39, 47, 53, 59, 70, 71, 73)
Anemia	18 (18)	(12, 19, 29, 34, 36–39, 43, 47, 48, 53–55, 63, 64, 70, 71, 73)
Severe anemia	3 (3)	(12, 63, 64)
Iron deficiency anemia	4 (4)	(29, 34, 47, 71, 74)
Serum iron	2 (2)	(51, 65)
Zinc protoporphyrin	2 (2)	(53, 74)
Plasma/serum copper	13 (13)	(51, 65)
Erythrocyte superoxide dismutase	2 (2)	(18, 72)
Elevated C-reactive protein	3 (3)	(36, 43, 70)
Lactulose:mannitol ratio	2 (2)	(18, 60)
Serum total cholesterol	3 (3)	(8, 17, 72)
*Physical or clinical descriptive outcomes*		
Vomiting	13 (0)	(8, 27, 30, 36, 41, 44–46, 49, 50, 67, 69)
Regurgitation	5 (0)	(8, 33, 46, 67, 69)
Nausea	2 (0)	(44, 45)
Constipation	4 (0)	(44, 45, 49, 50)
Abdominal pain	3 (0)	(41, 44, 45)
Drowsiness	2 (0)	(44, 45)
Mouth irritation	2 (0)	(44, 45)
Taste aversion	3 (0)	(33, 44, 45)
Respiratory infection	13 (0)	(13, 18, 29, 33, 34, 49, 50, 55, 57–59, 61, 75)
URTI	3 (0)	(33, 34, 50)
Bronchiolitis	2 (0)	(33, 36)
Cough	2 (0)	(29, 72)
Diarrhea	27 (0)	(8, 13, 18, 26, 27, 29–34, 36, 42–45, 49, 50, 55, 56, 58, 59, 61, 63, 67, 72, 75)
Dysentery/bloody diarrhea	3 (0)	(36, 42, 55)
ORS use	2 (0)	(27, 56)
Malaria	9 (0)	(18, 43, 55, 58, 63, 64, 70, 73, 74)
Fever	5 (0)	(29, 34, 59, 72, 74)
Death	7 (0)	(31, 33, 36, 43, 49, 58, 63)
Other	23 (0)	(8, 18, 20, 21, 31–34, 36, 41–45, 50, 51, 56, 62–64, 68, 72, 74)

1 ORS, oral rehydration solution; URTI, upper respiratory tract infection.

2 Number of studies reporting the outcome in the review (no. studies included in meta-analysis).

3 Includes 2 values converted from hematocrit.

4 No SD values reported.

5 Not included in meta-analysis because same study as that of Rosado et al. (59).

Two articles, Hess et al. (43) and Sazawal et al. (63), were the same as the studies of Abbeddou et al. (74) and Olney et al. (53), respectively, but reported additional data, therefore all four articles were included in this review. Two articles that reported on the same study but measured outcomes at different timepoints following zinc supplementation [Muñoz et al. (52) at 6 mo and Rosado et al. (59) at 12 mo] were included in the review, but only data from Rosado et al. (59) were considered for meta-analyses.

Almost all included studies were RCTs (*n* = 52) (12, 13, 26–75) and 4 were quasi-experimental (8, 17–19).

Daily doses of zinc ranged from 3 mg/d to 45 mg/d, from zinc supplementation or fortification. Six studies (8, 19, 34, 48, 50, 72) administered doses <5 mg/d, 34 studies (in 36 articles: 12, 13, 17, 26, 29–31, 35–43, 47, 51, 53–56, 60–65, 68–75) reported administering doses of 5 to 10 mg/d, 10 studies (in 11 articles) (28, 44, 46, 49, 52, 56–59, 67, 69) reported the effects of doses between 10.1 and 20 mg/d, and in 6 studies children consumed doses of between 21 and 45 mg/d (20, 21, 33, 45, 49, 67).

In 1 study children received a dose of 50 mg weekly (42), and 2 studies gave a 70-mg bolus either weekly (66) or twice weekly (18).

Only 10 studies (13, 17, 19, 34, 37, 40, 47, 48, 60, 75) included in the meta-analyses included dietary zinc intake data. Estimated dietary zinc intake from the studies included in the meta-analyses can be found in Table 2 **of Supplemental File 2**.

A total of 39 studies from 41 articles (8, 12, 13, 17–19, 26, 28–40, 43, 47, 48, 51, 53–55, 58–60, 62–65, 67, 70–75) were included in meta-analyses. The outcomes that were examined through meta-analyses were hemoglobin, anemia, serum ferritin, serum copper, iron deficiency, serum transferrin receptor (sTfR), hematocrit, C-reactive protein (CRP), erythrocyte superoxide dismutase (eSOD), zinc protoporphyrin (ZPP), serum total cholesterol, lactulose:mannitol molar ratio, and serum iron concentration.

A total of 32 studies (8, 13, 18, 26, 27, 29–34, 36, 41–46, 49, 50, 55, 56, 58, 59, 61, 63, 67, 69, 72, 74, 75, 75) included physical and clinical outcomes (i.e., vomiting, regurgitation, nausea, constipation, abdominal pain, drowsiness, mouth irritation, taste aversion, diarrhea, and dysentery). These studies are summarized narratively.

The GRADE, RoB, and JBI quality assessment of the studies can be found in **Supplemental File 3**. Less than 20% of studies were at high risk of bias using Cochrane RoB 2 criteria, with risk of bias in the randomization process being the main contributor. After consideration of risk of bias, inconsistency, indirectness, imprecision, and publication bias, GRADE certainty of evidence assessments for meta-analyses ranged from very low to moderate quality. Certainty of the evidence was primarily downgraded for risk of bias, indirectness (where study populations included some older children and/or had underlying morbidities), and imprecision.

### Meta-analyses of the biochemical outcomes

Outcome measures from all studies were mapped and, where reported in ≥2 studies, were considered for meta-analysis. A summary of the biochemical outcome measures considered for meta-analyses is presented in Table 2. Where possible, forest plots have been generated for the following: age category (0–90 d, 91–180 d, 6 to <12 mo, ≥12 mo); dose of zinc given (<5 mg/d, 5–10 mg/d, 10.1–20 mg/d, >20 mg/d, bolus); duration of intervention (0–3 mo, >3 mo); chemical formula of zinc (gluconate, sulfate, acetate, not stated, other); zinc compared with placebo; and high compared with low dose of zinc.

### Hemoglobin

**FIGURE 2:**
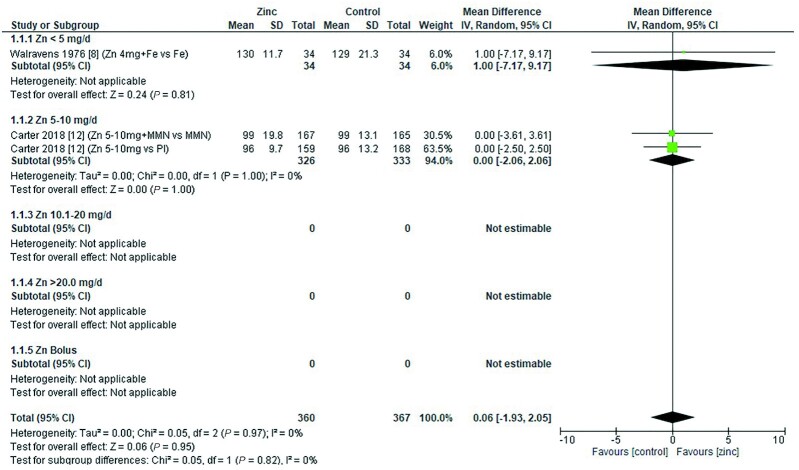
Effect of zinc supplementation on hemoglobin (g/L) in children aged 0–90 d by zinc dose. IV, inverse variance; Random, random effect model; 95% CI, 95% confidence interval; MMN, multiple micronutrients; Pl, placebo.

**FIGURE 3:**
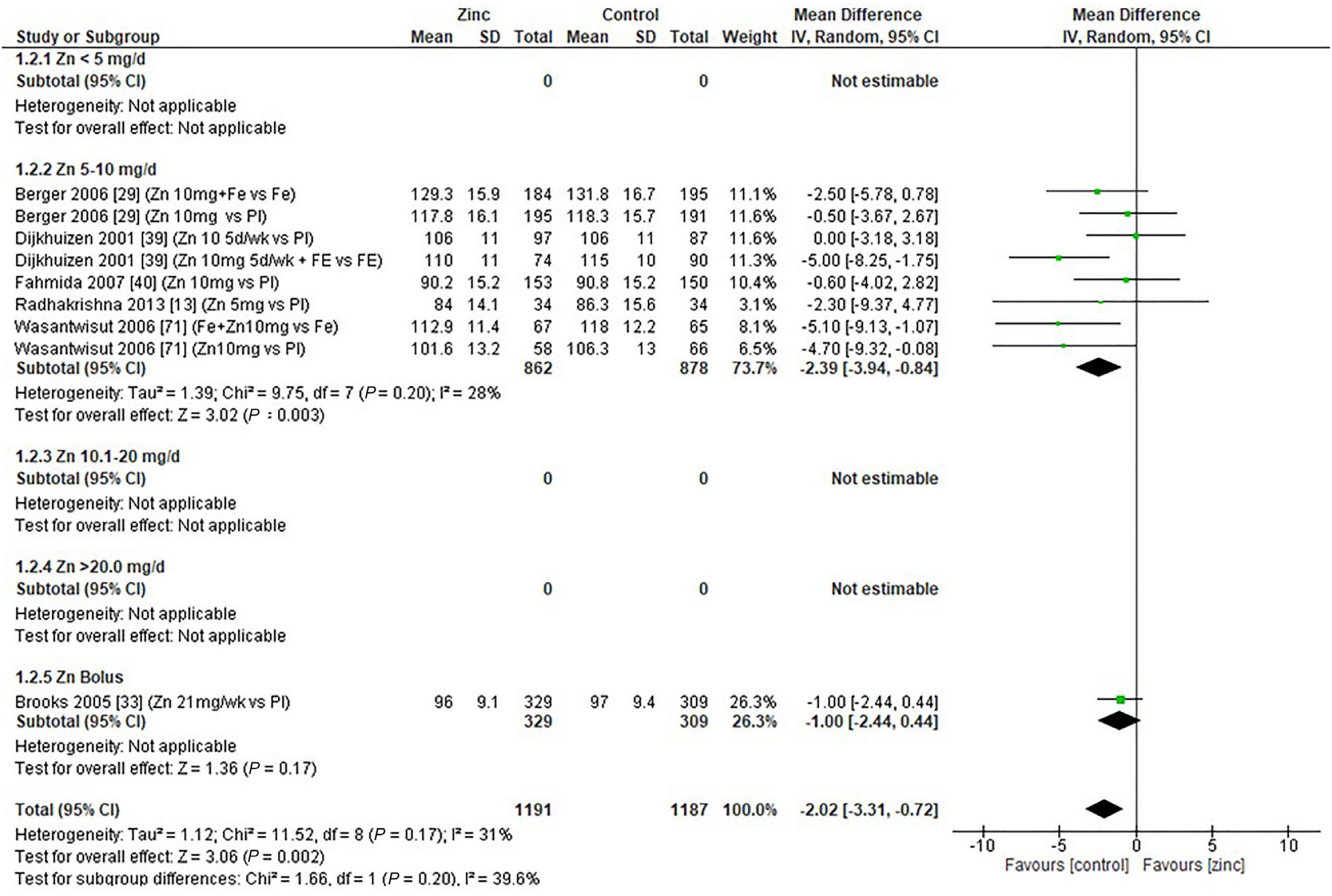
Effect of zinc supplementation on hemoglobin (g/L) in children aged 91–180 d by zinc dose. IV, inverse variance; Random, random effect model; 95% CI, 95% confidence interval; Pl, placebo.

**FIGURE 4:**
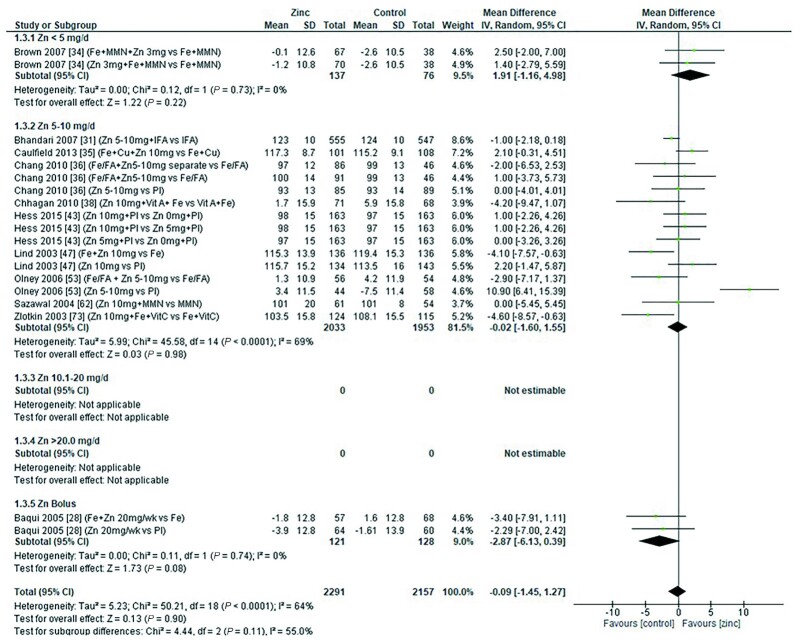
Effect of zinc supplementation on hemoglobin (g/L) in children aged >6 mo to 12 mo by zinc dose. FA, folic acid.; IFA, iron plus folic acid; IV, inverse variance; Random, random effect model; 95% CI, 95% confidence interval; MMN, multiple micronutrients; Pl, placebo; Vit A, vitamin A; Vit C, vitamin C.

**FIGURE 5:**
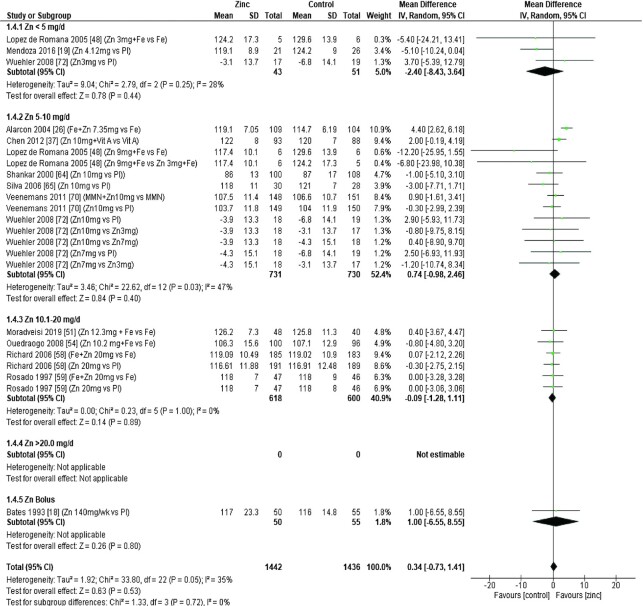
Effect of zinc supplementation on hemoglobin (g/L) in children aged >12 mo by zinc dose. IV, inverse variance, Random, random effect model; 95% CI, 95% confidence interval; MMN, multiple micronutrients; Pl, placebo; Vit A, vitamin A.

Thirty-two studies (8, 12, 13, 18, 19, 26, 28, 29, 31, 33–40, 43, 47, 48, 51, 53, 54, 58, 59, 62, 64, 65, 70–73) assessed the effect of zinc on hemoglobin, all of which were included in the meta-analyses. The pooled analyses of the effect of zinc supplementation on hemoglobin concentration (grams per liter) by age and dose, showed that overall, there was no evidence for an impact of zinc on hemoglobin concentrations in infants aged between 0 and 90 d when doses of 4–10 mg/d were provided (**Figure 2**). In children aged 91–180 d, doses of 5–10 mg/d were associated with a significant reduction in hemoglobin concentration [mean difference (MD): −2.39 g/L; 95% CI: −3.94, −0.84 g/L; *I*^2^ = 28%] (**Figure 3**). Pooled analyses of studies with children aged 6–12 mo (**Figure 4**) and >12 mo (**Figure 5**) showed that at the doses assessed, there were no significant effects on hemoglobin concentration.

No significant effects of zinc on hemoglobin concentrations were found in studies grouped by study duration, chemical formula, or comparator group, as summarized in **Table 3**. The quality of evidence for hemoglobin assessed using GRADE ranged from very low to moderate (Supplemental File 3).

**TABLE 3:** Summary of forest plot analyses of the effect of zinc supplementation intake in infants and children aged ≤3 y on hemoglobin (g/L), analyzed by duration, chemical formula, and comparator group^1^

Group/subgroup	References	Mean difference	95% CI	*P*	*I* ^2^
Children receiving interventions for <3 mo by zinc dose					
<5 mg/d	(48)	−5.40	−24.21, 13.41		NA
5–10 mg/d	(48, 73)	−5.26	−8.98, −1.54		0
10.1-20 mg/d	(51)	0.40	−3.67, 4.47		NA
Overall		−3.13	−6.82, 0.57	0.10	24
Children receiving interventions for >3 mo by zinc dose					
<5 mg/d	(8, 19, 34, 72)	0.39	−2.70, 3.48		33
5–10 mg/d	(12, 13, 26, 29, 31, 35–40, 43, 47, 53, 62, 64, 65, 70–72)	−0.21	−1.20, 0.77		63
10.1–20 mg/d	(54, 58, 59)	−0.13	−1.39, 1.12		0
Zn bolus^2^	(18, 28, 33)	−1.24	−2.53, 0.06		0
Overall		−0.25	−1.00, 0.50	0.51	54
Children receiving zinc through zinc sulfate by zinc dose					
<5 mg/d	(8, 34, 48, 72)	1.82	−0.89, 4.53		0
5–10 mg/d	(12, 13, 26, 29, 35, 39, 40, 47, 48, 65, 71, 72)	−0.97	−2.51, 0.56		66
10.1–20 mg/d	(54, 58, 59)	−0.03	−1.54, 1.49		0
Overall		−0.46	−1.60, 0.68	0.43	54
Children receiving zinc through zinc acetate by zinc dose					
Zn bolus^2^	(28, 33)	−1.30	−2.62, 0.01		0
Overall		−1.30	−2.62, 0.01	0.05	0
Children receiving zinc through an unstated approach by zinc dose					
5–10 mg/d	(31, 36, 43, 53, 70)	0.59	−1.01, 2.18		66
10.1–20 mg/d	(54)	−0.80	−4.80, 3.20		NA
Overall		0.47	−1.01, 1.96	0.53	0
Children receiving zinc through an “other” approach by zinc dose					
<5 mg/d	(19)	−5.10	−10.24, 0.04		NA
10.1–20 mg/d	(59)	0.00	−2.24, 2.24		0
Overall		−1.06	−3.73, 1.62	0.44	37
Children receiving zinc vs. placebo by zinc dose					
<5 mg/d	(19, 72)	−1.12	−9.70, 7.47		73
5–10 mg/d	(12, 13, 29, 36, 39, 40, 43, 47, 53, 64, 65, 70–72)	0.25	−1.11, 1.61		52
10.1–20 mg/d	(58, 59)	−0.18	−2.09, 1.73		0
Zn bolus^2^	(18, 28, 33)	−1.04	−2.39, 0.31		0
Overall		−0.10	−1.10, 0.90	0.84	43
Children receiving low- compared with high-dose zinc					
5–10 mg/d	(43, 48, 72)	0.35	−1.89, 2.58		0
Overall		0.35	−1.89, 2.58	0.76	0

1 NA, not applicable.

2 Bolus doses given as follows: Brooks et al. (33) 21 mg given weekly; Baqui et al. (28) 20 mg given weekly; Bates et al. (18) 70 mg given twice weekly.

### Anemia and severe anemia

**FIGURE 6:**
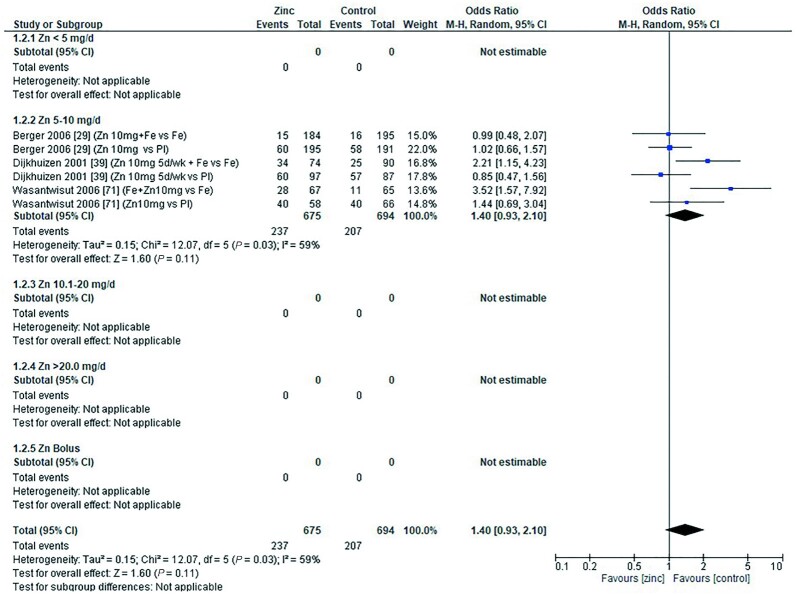
Effect of zinc supplementation on anemia (OR) in children aged 91–180 d by zinc dose. M-H, Mantel-Haenszel test; Random, random effect model; 95% CI, 95% confidence interval; Pl, placebo.

**FIGURE 7:**
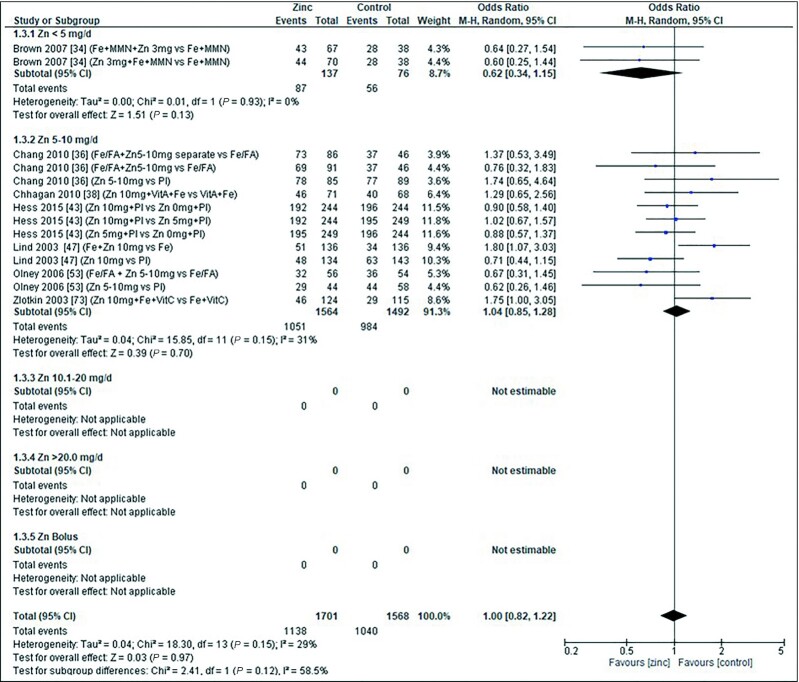
Effect of zinc supplementation on anemia (OR) in children aged >6 mo to 12 mo by zinc dose. FA, folic acid; M-H, Mantel-Haenszel test; Random, random effect model; 95% CI, 95% confidence interval; MMN, multiple micronutrients; Pl, placebo; Vit A, vitamin A; Vit C, vitamin C.

**FIGURE 8:**
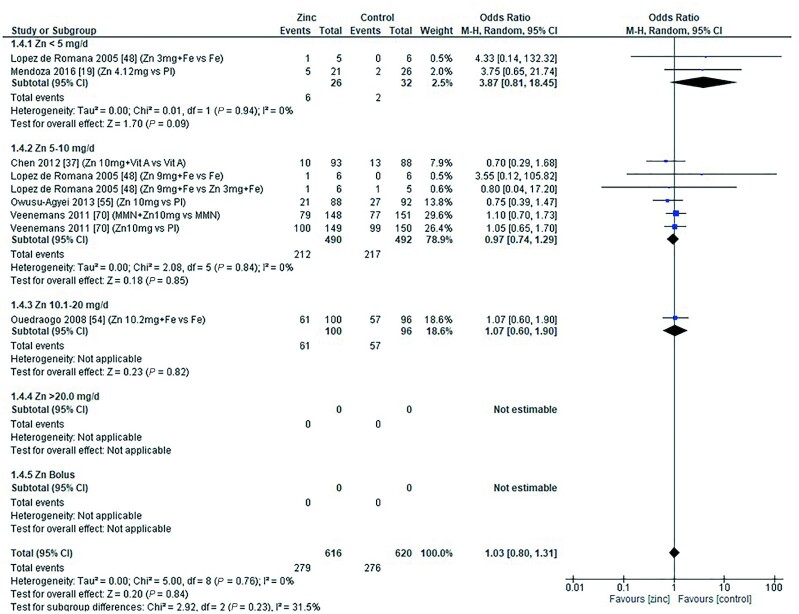
Effect of zinc supplementation on anemia (OR) in children aged >12 mo by zinc dose. M-H, Mantel-Haenszel test; Random, random effect model; 95% CI, 95% confidence interval; MMN, multiple micronutrients; Pl, placebo; Vit A, vitamin A.

The effect of zinc supplementation on the OR for anemia (defined as hemoglobin less than 10.0–11.0g/dL) was explored in all age categories. Only 1 study, by Carter et al. in 2018 (12), included children aged 0–90 d. Doses of 5–10 mg/d zinc were provided with no significant effect on the OR for anemia (OR = 1.20; 95% CI: 0.89, 1.60; *I*^2^ = not applicable). Three studies (29, 39, 71) (6 data sets) included children aged 91–180 d. All studies administered doses of 5–10 mg/d with and without additional iron, compared with iron alone or placebo, respectively (**Figure 6**). In children aged 6–12 mo, 7 studies (34, 36, 38, 43, 47, 53, 73) (14 comparisons) examined doses of zinc with and without combinations of iron, multiple micronutrients (MMN), and vitamin C (**Figure 7**). In children aged >12 mo, 6 studies (19, 37, 48, 54, 55, 70) (9 comparisons) investigated zinc supplementation with and without combinations of vitamin A, iron, and MMN (**Figure 8**). In all age groups, there was no significant effect of zinc supplementation on the OR for anemia.

Pooled analyses of the OR for anemia by duration of intervention, chemical formula of zinc supplement, and comparator (placebo or high compared with low zinc dose) are summarized in **Table 4**. Zinc supplementation had no effect on the OR for anemia when data were grouped by chemical formula and comparator. When grouped by study duration, 2 studies (48, 73) had interventions that lasted for 0–3 mo, and 15 studies (12, 19, 29, 34, 36–39, 43, 47, 53–55, 70, 71) (26 comparisons) had interventions that lasted >3 mo. In both duration categories children received doses of 3–10 mg/d. For studies with interventions of a shorter duration, the OR for anemia increased significantly with zinc supplementation (Table 4). For studies of longer duration, the overall OR for developing anemia was not significantly changed by zinc supplementation.

**TABLE 4:** Summary of forest plot analyses of the effect of zinc supplementation in infants and children aged ≤3 y on anemia (OR), analyzed by duration, chemical formula, and comparator group^1^

Group/subgroup	References	Mean difference	95% CI	*P*	*I* ^2^
Children receiving interventions for <3 mo by zinc dose					
<5 mg/d	(48)	4.33	0.14, 132.32		NA
5–10 mg/d	(48, 73)	1.74	1.01, 2.98		0
Overall		1.78	1.04, 3.03	0.03	0
Children receiving interventions for >3 mo by zinc dose					
<5 mg/d	(19, 34)	0.85	0.37, 1.95		44
5–10 mg/d	(12, 29, 36–39, 43, 47, 53–55, 70, 71)	1.08	0.93, 1.25		30
10.1–20 mg/d	(54)	1.07	0.60, 1.90		NA
Overall		1.06	0.92, 1.22	0.43	28
Children receiving zinc through zinc gluconate by zinc dose					
5–10 mg/d	(37, 38, 55, 73)	1.10	0.71, 1.72		41
Overall		1.10	0.71, 1.72	0.66	41
Children receiving zinc through zinc sulfate by zinc dose					
<5 mg/d	(34, 48)	0.66	0.36, 0.121		0
5–10 mg/d	(12, 29, 39, 47, 48, 71)	1.28	0.98, 1.67		49
Overall		1.18	0.92, 1.53	0.19	47
Children receiving zinc through an unstated approach by zinc dose					
5–10 mg/d	(36, 43, 53, 70)	0.97	0.81, 1.15		0
10.1–20 mg/d	(54)	1.07	0.60, 1.90		NA
Overall		0.97	0.82, 1.15	0.76	0
Children receiving zinc vs. placebo by zinc dose					
<5 mg/d	(19)	3.75	0.65, 21.74		NA
5–10 mg/d	(29, 36, 39, 43, 47, 53, 55, 70, 71)	0.89	0.75, 1.05		0
Overall		0.90	0.76, 1.06	0.21	0
Children receiving low compared with high dose zinc					
5–10 mg/d	(43, 48)	1.13	0.83, 1.53		0
Overall		1.13	0.83, 1.53	0.44	0

1 NA, not applicable.

The impact of zinc supplementation on the risk of severe anemia was examined by 3 studies (12, 63, 64), and defined as hemoglobin concentration <70 g/L in 2 studies (63, 64) and <85 g/L in 1 study (12). Combining data from these 3 studies did not reveal a significant impact of zinc supplementation at doses of 5–10 mg/d on the OR for severe anemia (OR: 1.00; 95% CI: 0.77, 1.28; *I*^2^ = 0%).

The quality of evidence for anemia assessed using GRADE ranged from very low to moderate (Supplemental File 3).

### Serum ferritin

**FIGURE 9:**
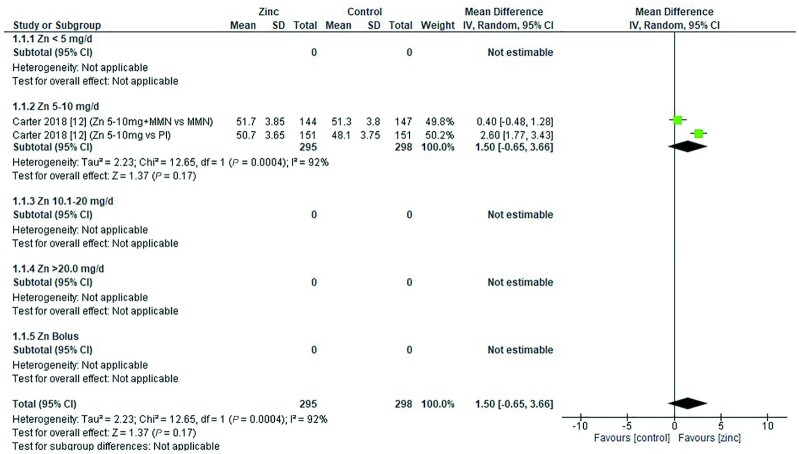
Effect of zinc supplementation on serum ferritin (μg/L) in children aged 0–90 d by zinc dose. IV, inverse variance; Random, random effect model; 95% CI, 95% confidence interval; MMN, multiple micronutrients; Pl, placebo.

**FIGURE 10:**
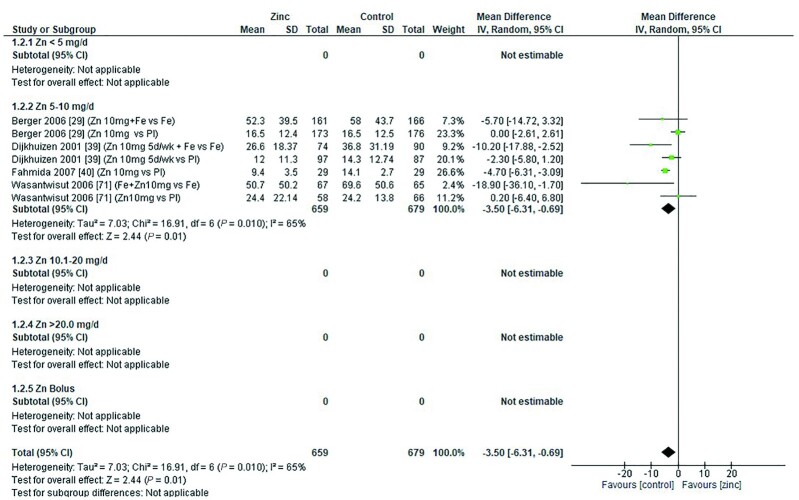
Effect of zinc supplementation on serum ferritin (μg/L) in children aged 91–180 d by zinc dose. IV, inverse variance; Random, random effect model; 95% CI, 95% confidence interval; Pl, placebo.

**FIGURE 11:**
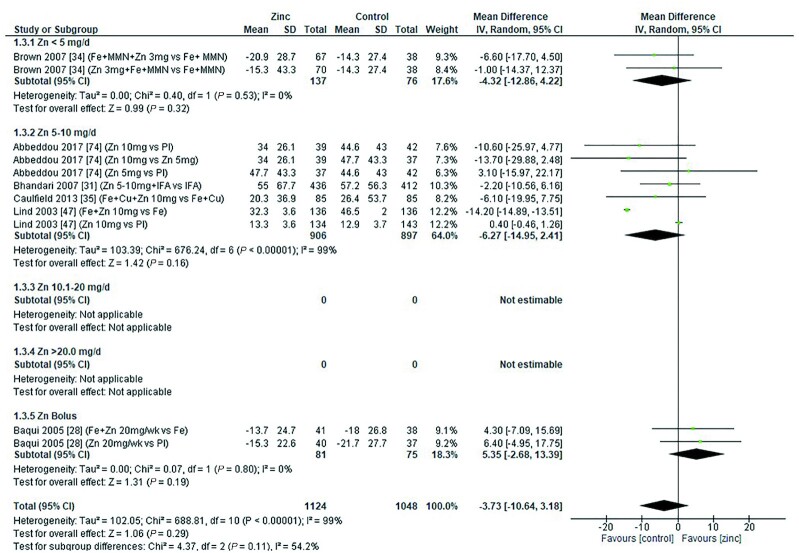
Effect of zinc supplementation on serum ferritin (μg/L) in children aged >6 mo to 12 mo by zinc dose. IFA, iron plus folic acid; IV, inverse variance; Random, random effect model, 95% CI; 95% confidence interval; MMN, multiple micronutrients; Pl, placebo.

**FIGURE 12:**
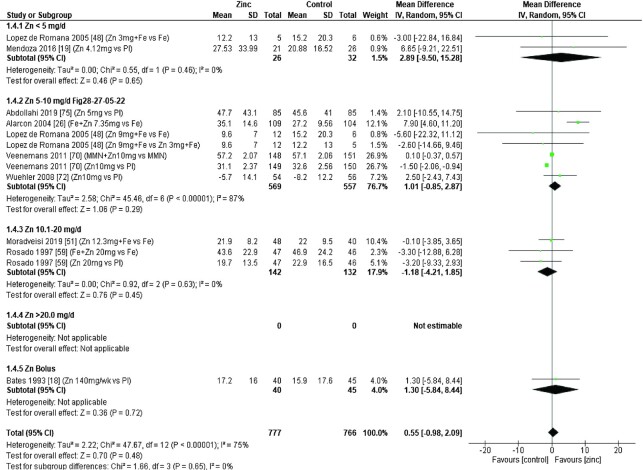
Effect of zinc supplementation on serum ferritin (μg/L) in children aged >12 mo by zinc dose. IV, inverse variance; Random, random effect model; 95% CI, 95% confidence interval; MMN, multiple micronutrients; Pl, placebo.

The effect of zinc supplementation on serum ferritin (micrograms per liter) in children was assessed by age category and dose. Meta-analysis showed that in children aged 91–180 d, a dose of 10 mg/d resulted in a significantly lower serum ferritin concentration than in controls. However, no significant effect was observed in children aged 0–90 d, 6–12 mo, or >12 mo at any of the doses assessed (**Figures 9**–**12**).

When assessed by dose and duration of intervention (across all ages), no significant difference was found in serum ferritin concentration compared with controls either in interventions lasting <3 mo or in those lasting >3 mo (**Table 5**). No significant effect associated with the zinc chemical formula could be detected with the data available (Table 5).

**TABLE 5:** Summary of forest plot analyses of the effect of zinc supplementation in infants and children aged ≤3 y on serum ferritin (μg/L), analyzed by duration, chemical formula, and comparator group^1^

Group/subgroup	References	Mean difference	95% CI	*P*	*I* ^2^
Children receiving interventions for <3 mo by zinc dose					
<5 mg/d	(48)	−3.00	−22.84, 16.84		NA
5–10 mg/d	(48)	−3.79	−16.21, 8.63		0
10.1–20 mg/d	(51)	−0.10	−3.85, 3.65		NA
Overall		−0.49	−4.02, 3.04	0.79	0
Children receiving interventions for >3 mo by zinc dose					
<5 mg/d	(19, 34)	−1.85	−9.37, 5.67		0
5–10 mg/d	(12, 26, 29, 31, 35, 39, 40, 47, 70–72, 74, 75)	−2.32	−5.18, −0.53		99
10.1–20 mg/d	(59)	−3.23	−8.40, 1.94		0
Zn bolus	(18, 28)	3.09	−2.25, 8.43		0
Overall		−1.80	−4.31, −0.72	0.16	98
Children receiving zinc through zinc gluconate by zinc dose					
Zn bolus^2^	(18)	1.30	−5.84, 8		NA
Overall		1.30	−5.84, 8	0.72	NA
Children receiving zinc through zinc sulfate by zinc dose					
<5 mg/d	(34, 48)	−4.11	−11.95, 3.73		0
5–10 mg/d	(12, 26, 29, 35, 39, 40, 47, 48, 71, 72, 75)	−2.50	−6.69, 1.70		99
10.1–20 mg/d	(51)	−0.10	−3.85, 3.65		NA
Overall		−2.48	−6.30, 1.34	0.20	99
Children receiving zinc through zinc acetate by zinc dose					
10.1–20 mg/d	(28)	5.35	−2.68, 13.39		0
Overall		5.35	−2.68, 13.39	0.19	0
Children receiving zinc through an unstated approach by zinc dose					
5–10 mg/d	(31, 70, 74)	−0.93	−2.46, 0.61		78
Overall		−0.93	−2.46, 0.61	0.24	78
Children receiving zinc through an “other” approach by zinc dose					
<5 mg/d	(19)	6.65	−9.21, 22.51		NA
10.1–20 mg/d	(59)	−3.23	−8.40, 1.94		0
Overall		−2.28	−7.19, 2.63	0.36	0
Children receiving zinc vs. placebo by zinc dose					
<5 mg/d	(19)	6.65	−9.21, 22.51		NA
5–10 mg/d	(12, 29, 39, 40, 47, 70–72, 74, 75)	−0.56	−2.35, 1.23		90
10.1–20 mg/d	(59)	−3.20	−9.33, 2.93		NA
Zn bolus^2^	(18, 28)	2.75	−3.30, 8.79		0
Overall		−0.41	−2.08, 1.25	0.63	86
Children receiving low- compared with high-dose zinc					
5–10 mg/d	(48, 74)	−8.10	−18.98, 2.78		45
Overall		−8.10	−18.98, 2.78	0.14	45

1 NA, not applicable.

2 Bolus doses given as follows: Baqui et al. (28) 20 mg/d once weekly; Bates et al. (18) 70 mg/d twice weekly.

The quality of evidence for serum ferritin using GRADE ranged from very low to moderate (Supplemental File 3).

### Serum/plasma copper concentration

**FIGURE 13:**
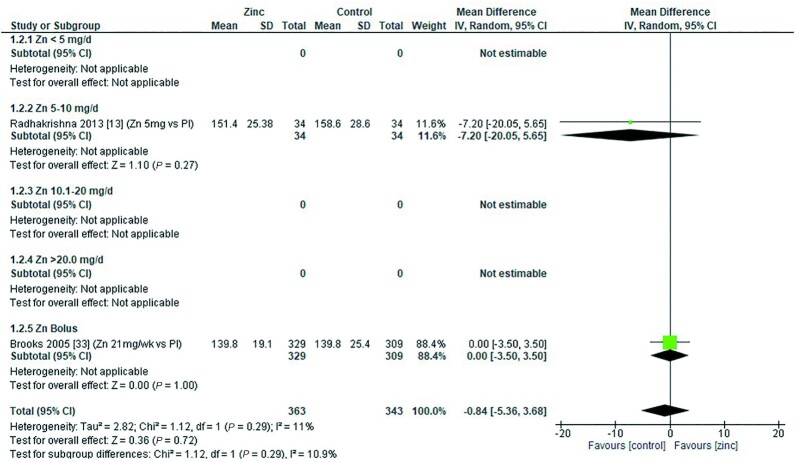
Effect of zinc supplementation on serum/plasma copper (μg/dL) in children aged 91–180 d by zinc dose. IV, inverse variance; Random, random effect model; 95% CI, 95% confidence interval; Pl, placebo.

**FIGURE 14:**
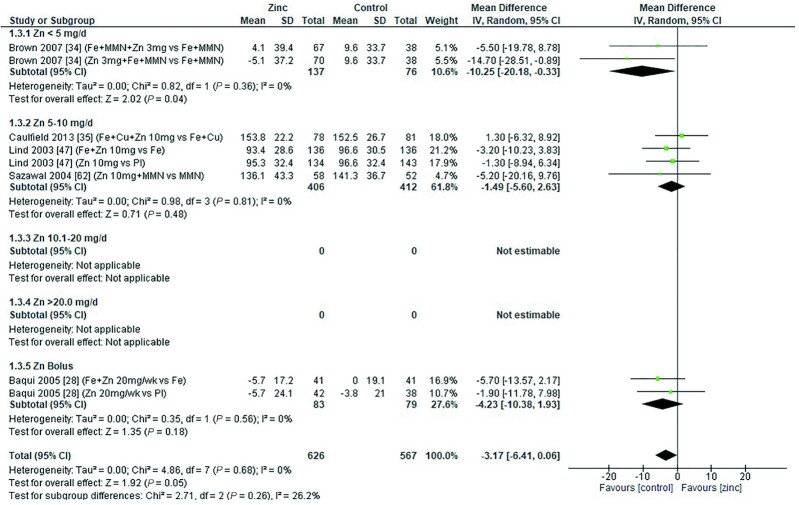
Effect of zinc supplementation on serum/plasma copper (μg/dL) in children aged >6 mo to 12 mo by zinc dose. MMN, multiple micronutrients; IV, inverse variance; Random, random effect model; 95% CI, 95% confidence interval Pl, placebo.

**FIGURE 15:**
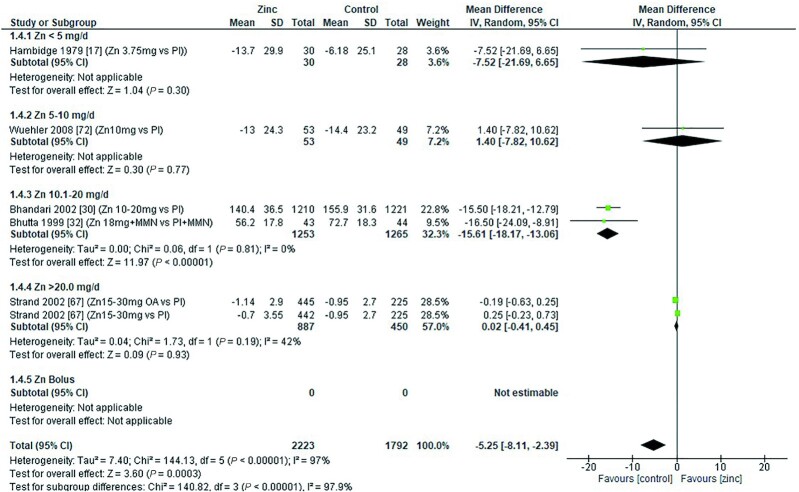
Effect of zinc supplementation on serum/plasma copper (μg/dL) in children aged >12 mo by zinc dose. IV, inverse variance; Random, random effect model; 95% CI, 95% confidence interval ; MMN, multiple micronutrients; OA, open arm; Pl, placebo.

Zinc supplementation had no significant effect on serum/plasma copper concentration (micrograms per deciliter) at any dose in children aged 0–3 mo [data from 1 study (8) *P* = 0.056] or those aged 3–6 mo (**Figure 13**). However, in age groups 6–12 mo and >12 mo (**Figures 14** and **15**), doses of 3–20 mg/d had a significant, negative effect on serum/plasma copper concentration.

The effect of zinc supplementation on serum copper concentrations across all age groups was analyzed by duration of intervention, chemical formula of zinc supplement, and comparator group (**Table 6**). From the 2 studies (32, 67) with interventions <3 mo, only 1 (32) reported a highly significant impact of a dose of 18 mg/d, given with MMN, compared with a placebo plus MMN in children >12 mo of age. For interventions >3 mo, data from 1 study (30) showed that a dose of 10–20 mg/d was associated with a significant reduction in serum/plasma copper concentration. This association was not shown for interventions with zinc doses of 0–10 mg/d zinc or a bolus of 20–21 mg/wk.

**TABLE 6:** Summary of forest plot analyses of the effect of zinc supplementation in infants and children aged ≤3 y on serum/plasma copper concentration (μg/dL), analyzed by duration, chemical formula, and comparator group^1^

Group/subgroup	References	Mean difference	95% CI	*P*	*I* ^2^
Children receiving interventions for <3 mo by zinc dose					
10.1–20 mg/d	(32)	−16.50	−24.09, −8.91		NA
>20 mg/d	(67)	0.02	−0.41, 0.45		42
Overall		−0.50	−1.90, 0.90	0.48	90
Children receiving interventions for >3 mo by zinc dose					
<5 mg/d	(8, 17, 34)	−4.62	−15.01, 5.78		50
5–10 mg/d	(13, 35, 47, 62, 72)	−1.50	−5.10, 2.11		0
10.1–20 mg/d	(30)	−15.50	−18.21, −12.79		NA
Zn bolus^2^	(28, 33)	−1.03	−4.08, 2.01		0
Overall		−4	−8.82, 0.82	0.10	82
Children receiving zinc through zinc gluconate by zinc dose					
5–10 mg/d	(62)	−5.20	−20.16, 9.76		NA
10.1–20 mg/d	(30)	−15.50	−18.21, −12.79		NA
Overall		−13.08	−21.64, −4.53	0.003	43
Children receiving zinc through zinc sulfate by zinc dose					
<5 mg/d	(34)	−10.25	−20.18, −0.33		0
5–10 mg/d	(8, 13, 35, 47, 72)	−0.63	−4.26, 2.99		0
10.1–20 mg/d	(32)	−16.50	−24.09, −8.91		NA
Overall		−4.09	−9.31, 1.12	0.12	61
Children receiving zinc through zinc acetate by zinc dose					
Zn bolus^2^	(28,33)	−1.03	−4.08, 2.01		0
Overall		−1.03	−4.08, 2.01	0.51	0
Children receiving zinc vs. placebo by zinc dose					
<5 mg/d	(17)	−7.52	−21.69, 6.65		NA
5–10 mg/d	(13, 47, 72)	−1.41	−6.76, 3.93		0
10.1–20 mg/d	(30, 32)	−15.61	−18.17, −13.06		0
>20 mg/d	(67)	0.02	−0.41, 0.45		42
Zn bolus^2^	(28, 33)	−0.21	−3.52, 3.09		0
Overall		−4.11	−6.48, −1.74	<0.001	94

1 NA, not applicable.

2 Bolus doses given as follows: Brooks et al. (33) 21/d mg given weekly; Baqui et al. (28) 20 mg/d once weekly; Bates et al. (18) 70 mg/d twice weekly.

Two studies used zinc gluconate, 1 at a dose of 10 mg/d of elemental zinc (62) and the other at 10–20 mg/d of elemental zinc depending on participant age (30). The overall effect was a significant reduction in serum/plasma copper concentration, mainly driven by the large effect at the higher dose. The overall effects of zinc sulfate, acetate, zinc-fortified breakfast (17), and nonstated forms of zinc were nonsignificant. However, studies using zinc sulfate at an elemental zinc dose of 3 mg/d (34) and 18 mg/d zinc with MMN (32) had a deleterious impact on serum/plasma copper concentrations. Combining studies where the comparator arm was a placebo, there was an overall significant effect of zinc on serum/plasma copper concentration (Table 5).

The quality of evidence for copper using GRADE ranged from very low to moderate (Supplemental File 3).

### Iron deficiency

**FIGURE 16:**
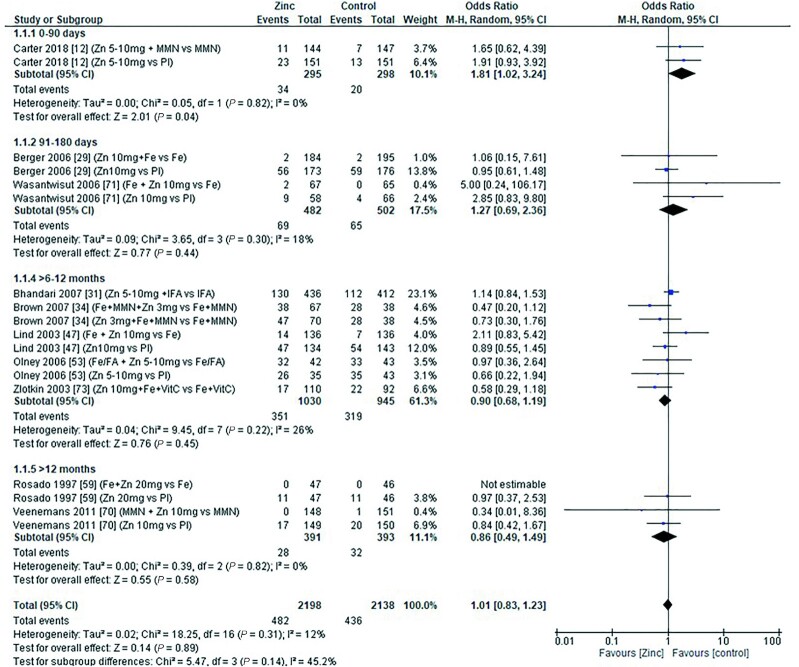
Effect of zinc supplementation in infants and children aged ≤3 y on iron deficiency (OR) by age group. Note: Zinc exposure corresponds to mg/d except for Rosado et al. (59) for which doses were given 6 d/wk. FA, folic acid; IFA, iron plus folic acid; M-H, Mantel-Haenszel test; Random, random effect model; 95% CI, 95% confidence interval; MMN, multiple micronutrients; Pl, placebo; Vit C, vitamin C.

Analysis of the effect of zinc supplementation on the OR for iron deficiency (serum ferritin concentration <12 μg/L) by age showed a significantly increased OR in infants aged <3 mo (**Figure 16**). However, this was derived from a single study (73), providing a dose of 10 mg/d zinc, with and without MMN. In older children, pooled analysis showed no evidence of a significant effect of zinc supplementation on the OR for iron deficiency at the doses, intervention duration, or chemical formula used in the studies **(Table****7**).

The quality of evidence for iron deficiency using GRADE ranged from very low to moderate (Supplemental File 3).

### Iron deficiency anemia

**FIGURE 17:**
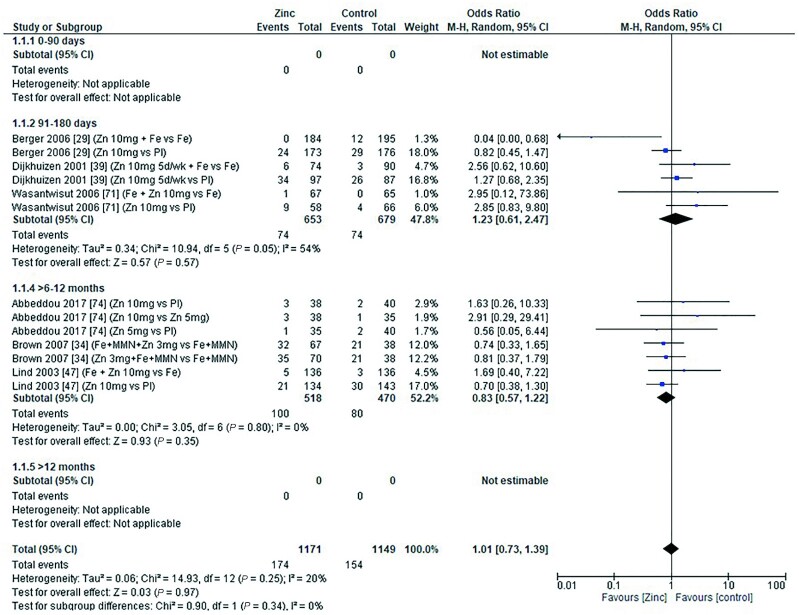
Effect of zinc supplementation in infants and children aged ≤3 y on iron deficiency anemia (OR) by age group. Note: Zinc exposure corresponds to mg/d. M-H, Mantel-Haenszel test; Random, random effect model; 95% CI, 95% confidence interval; MMN, multiple micronutrients; Pl, placebo.

No significant association was observed between zinc intake and the odds of iron deficiency anemia (defined as having both anemia and iron deficiency) in any of the assessed age categories (**Figure 17**), zinc dose, duration of dose, or zinc chemical formula (**Table 8**). No studies were conducted in children aged <3 mo or >12 mo.

The quality of evidence for iron deficiency using GRADE ranged from very low to low (Supplemental File 3).

### Serum transferrin receptor

**FIGURE 18:**
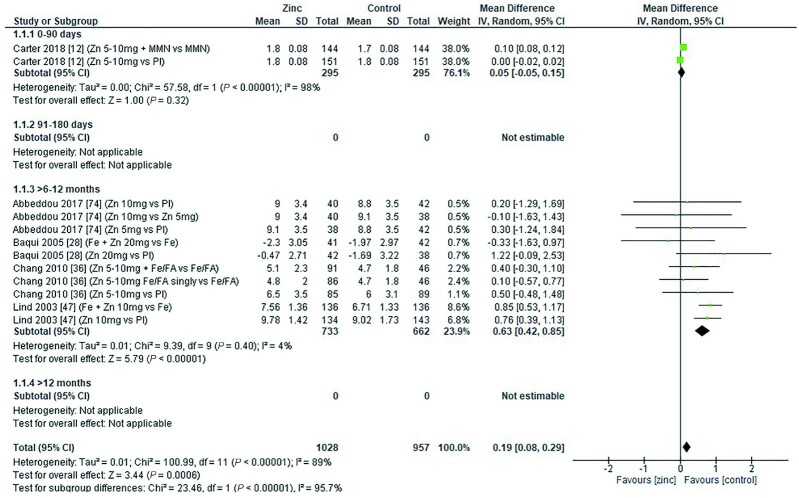
Effect of zinc supplementation in infants and children aged ≤3 y on serum/soluble transferrin receptor (mg/L) by age group. Note: Zinc exposure corresponds to mg/d except for Baqui et al. (28) for which dose was 20 mg/d once weekly, and Chang et al. (36) for which dose was given in alternate days. FA, folic acid; IV, inverse variance; Random, random effect model; 95% CI, 95% confidence interval; MMN, multiple micronutrients; Pl, placebo.

Five studies examined the impact of zinc (5–20 mg/d) on sTfR (milligrams per liter). Of these, 1 study (12) analyzed the effect of zinc on sTfR in children aged 0–3 mo; results showed no significant effect of a dose of 5–10 mg/d on sTfR (**Figure 18**). Four studies (28, 36, 47, 74) examined the impact of 5–20 mg/d zinc on sTfR in children aged 6–12 mo. Overall, there was a significant impact of zinc on sTfR, indicating worsening iron status with zinc supplementation (Figure 18). No data were available for children aged 91–180 d or for those aged >12 mo.

A statistically significant negative impact on sTfR was observed when zinc sulfate was administered. No significant effects were observed when data were combined by other chemical formula, duration of intervention, or comparator (**Table 9**).

The quality of evidence for sTfR using GRADE ranged from very low to moderate (Supplemental File 3).

### Hematocrit

**FIGURE 19:**
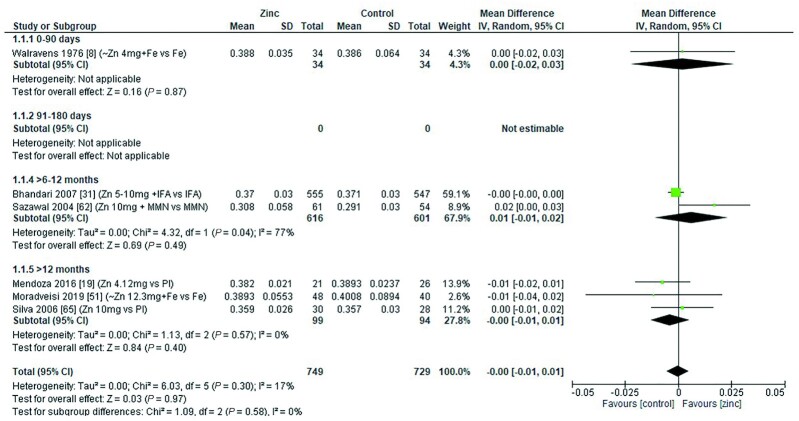
Effect of zinc supplementation in infants and children aged ≤3 y on hematocrit (proportion) by age group. Note: Zinc exposure corresponds to mg/d. IFA, iron folic acid; IV, inverse variance; Random, random effect model; 95% CI, 95% confidence interval; MMN, multiple micronutrients; Pl, placebo.

There was no overall effect of zinc supplementation on hematocrit in children aged 0–3 mo, 6–12 mo, or >12 mo (**Figure 19**). Zinc doses ranged from 4 to 12.3 mg/d, with additional supplements including iron, folic acid, and MMN. The duration of the intervention was >3 mo in all studies except that of Moradveisi et al. (51). When examined by chemical formula, 1 study reported that zinc gluconate had a significant positive effect on hematocrit (MD = 0.02%; 95% CI: 0.00, 0.03%; *P* = 0.04) (62). No other significant effects were observed (**Table 10**).

The quality of evidence for hematocrit using GRADE ranged from very low to moderate (Supplemental File 3).

### C-reactive protein

**FIGURE 20:**
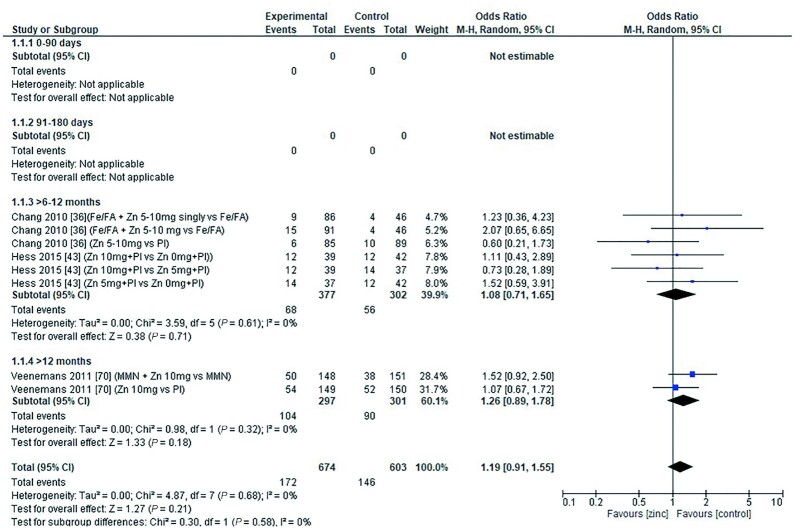
Effect of zinc supplementation in infants and children aged ≤3 y on raised C-reactive protein by age group. Note: Zinc exposure corresponds to mg/d except for Chang et al. (36) for which dose was given in alternate days. FA, folic acid; M-H, Mantel-Haenszel test; Random, random effect model; 95% CI, 95% confidence interval; MMN, multiple micronutrients; Pl, placebo.

Data assessing the effect of zinc intake on CRP were limited, and there are no data in children aged <6 mo, but data suggest there is no association between zinc supplementation and the likelihood of raised CRP in children aged >6 mo (**Figure 20**). The duration of the intervention for all studies was >3 mo. None of the studies stated the chemical formula of the zinc administered. The combined data from all studies showed no significant effect of duration of treatment or chemical formula on the likelihood of raised CRP (**Table 11**).

The quality of evidence for CRP using GRADE was low (Supplemental File 3).

### Erythrocyte SOD

**FIGURE 21:**
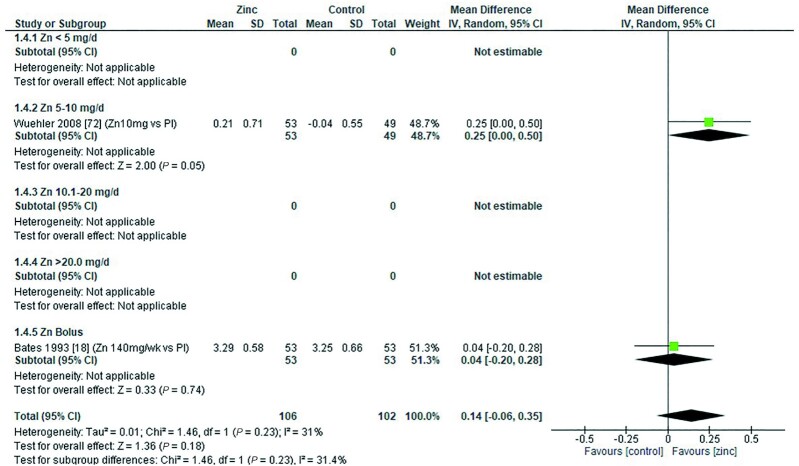
Effect of zinc supplementation on erythrocyte superoxide dismutase (IU/mg Hb) in children aged >12 mo by zinc dose. Note: Zinc exposure corresponds to mg/d. IV inverse variance; Random, random effect model; 95% CI, 95% confidence interval; Pl, placebo.

Two studies reported the effect of zinc supplementation on eSOD. Wuehler et al. (72) provided a dose of 10 mg/d elemental zinc (zinc sulfate), and Bates et al. (18) administered 2 bolus doses of 70 mg elemental zinc per week (zinc gluconate). Both studies compared zinc with a placebo in children aged >12 mo. Combining the data from the 2 studies showed no overall significant effect on eSOD (**Figure 21**).

The quality of evidence for eSOD using GRADE was low (Supplemental File 3).

### Zinc protoporphyrin

**FIGURE 22:**
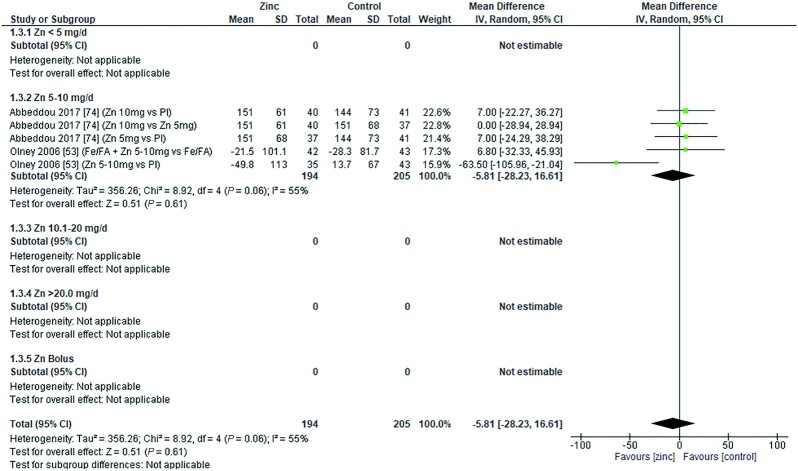
Effect of zinc supplementation on zinc protoporphyrin (μmol/mol heme) in children >6 mo to 12 mo by zinc dose. Note: Zinc exposure corresponds to mg/d. FA, folic acid; IV, inverse variance; Random, random effect model; 95% CI, 95% confidence interval; Pl, placebo.

Two studies (53, 74) measured ZPP following doses of 5–10 mg/d for a period >3 mo. The pooled analysis revealed that zinc supplementation had no significant effect on ZPP (**Figure 22**).

The quality of evidence for ZPP using GRADE was very low (Supplemental File 3).

### Serum total cholesterol

**FIGURE 23:**
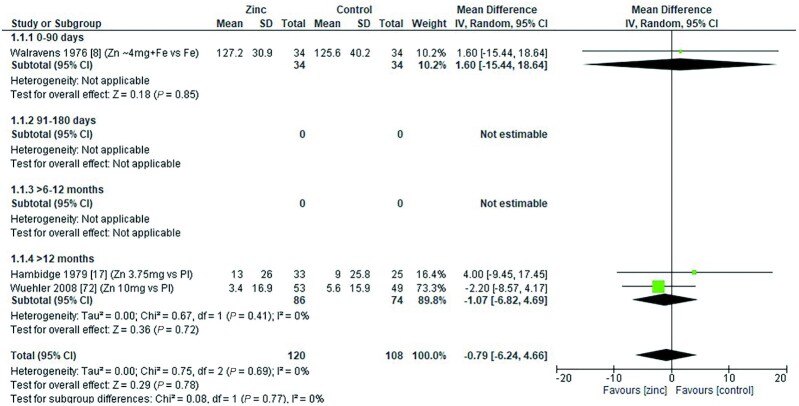
Effect of zinc supplementation in infants and children aged ≤3 y on serum total cholesterol (mg/dL) by duration of treatment. Note: Zinc exposure corresponds to mg/d except for Hambidge et al. (17) for which doses were given 6 days per week. IV, inverse variance; Random, random effect model; 95% CI, 95% confidence interval; Pl, placebo.

Three studies assessed the effect of zinc on serum total cholesterol (milligrams per deciliter). One study (8) examined serum total cholesterol in children aged 0–3 mo, and 2 studies (17, 72) were conducted with children aged >12 mo. There was no significant effect on zinc in any age group (**Figure 23**). All 3 studies had an intervention period >3mo, with doses ranging from 3.75 to 10 mg/d elemental zinc, either in the form of zinc sulfate (8, 72), or in a fortified breakfast cereal (17). Grouping the studies by dose, mode of delivery, duration, and zinc compared with placebo did not reveal any significant impact of zinc supplementation on total blood cholesterol concentration (**Table 12**).

The quality of evidence for serum total cholesterol using GRADE ranged from very low to low (Supplemental File 3).

### Lactulose:mannitol molar ratio

**FIGURE 24:**
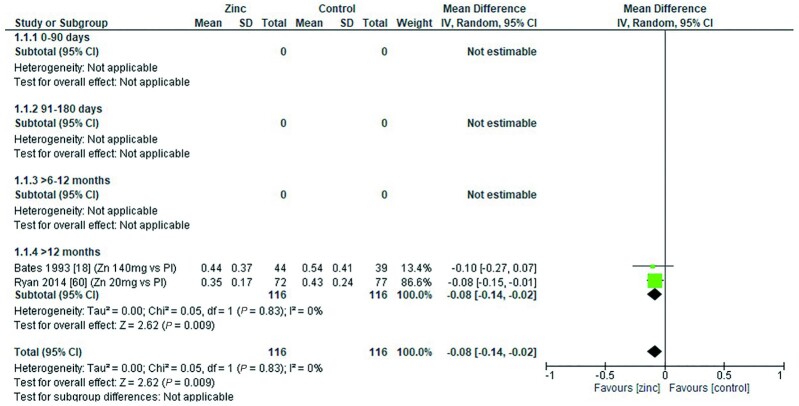
Effect of zinc supplementation in infants and children aged ≤3 y on lactulose:mannitol ratio by age group. Note: Zinc exposure corresponds to mg/d except for Bates et al. (18) for which doses of 70 mg/d were given twice weekly. IV, inverse variance; Pl, placebo.

Two studies (18, 60) reported the effect of zinc supplementation on lactulose:mannitol molar ratio. Both studies were conducted with children aged >12 mo. Overall, there was a significant reduction in the ratio following zinc supplementation, indicating reduced (improved) gut permeability (**Figure 24**). Ryan et al. (60) provided a dose of 20 mg/d elemental zinc in the form of zinc acetate for a period <3mo. This dose regimen yielded a significant reduction in the ratio compared with a placebo. Bates et al. (18) provided a dose of 70 mg elemental zinc, twice a week, in the form of zinc sulfate for a period >3 mo. There was a fall in the ratio compared with placebo, but it failed to reach statistical significance (**Table 13**).

The quality of evidence for lactulose:mannitol molar ratio using GRADE ranged from very low to low (Supplemental File 3).

### Serum iron concentration

**FIGURE 25:**
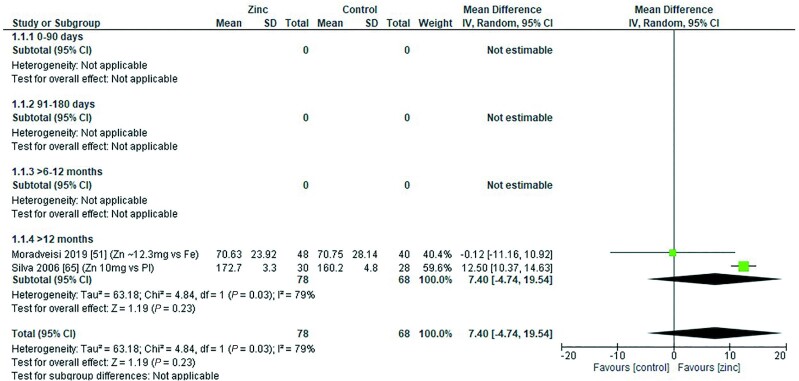
Effect of zinc supplementation in infants and children aged ≤3 y on serum iron concentration (μg/dL) by age group. IV, inverse variance; Random, random effect model, 95% CI, 95% confidence interval; Pl, placebo.

Two studies assessed the effect of zinc on serum iron concentration (micrograms per deciliter). Both studies were conducted in children aged >2 y (51, 65). Pooling data from both studies revealed no significant effect on serum iron concentrations (**Figure 25**). Moradveisi et al. (51) provided a dose of 12.3 mg/d elemental zinc as zinc sulfate with iron (60 mg/d) or iron alone (60 mg/d) for a period <3 mo with no significant effect. Silva et al. (65) provided a dose of 10 mg/d elemental zinc as zinc sulfate or a placebo for >3 mo. There was a significant increase in serum iron following zinc supplementation (**Table 14**).

The quality of evidence for serum iron using GRADE was very low (Supplemental File 3).

### Narrative description of physical and clinical outcomes

#### Vomiting, regurgitation, and nausea

Thirteen studies (8, 27, 30, 36, 41, 44–46, 49, 50, 67, 69) reported on the effect of zinc supplementation on the occurrence of vomiting (8, 27, 30, 36, 41, 44–47, 49, 50, 67, 69).

Overall, 4 (30, 46, 67, 69) of the 13 studies found a higher incidence of vomiting following zinc supplementation, with single daily zinc doses ranging from 10 to 20 mg for infants aged <12 mo, and 20 to 30 mg/d for children aged ≥12 mo. Three studies were of short duration (10–14 d) in children with acute diarrhea (46, 67) or pneumonia (69), and 1 study (30) was of 4 mo duration for the prevention of diarrhea. In Chang et al. (36), zinc provided at the same time as iron resulted in a significant increase in the frequency of vomiting compared with placebo or other intervention groups provided with zinc alone or zinc with iron separately.

Five studies (8, 33, 46, 67, 69) reported on posttreatment regurgitation. Three studies found an increased frequency of regurgitation in children with acute diarrhea (46, 67) or pneumonia (69), who received single daily zinc doses ranging from 10 to 20 mg (infants <12 mo of age) or 20 to 30 mg/d (children aged ≥12 mo) for 10–14 d. One study (33) that administered weekly zinc doses of 21 mg to children aged 2–11 mo for 12 mo for the prevention of diarrhea and pneumonia, cited taste aversion sometimes leading to regurgitation as a potential reason for the higher number of withdrawals in the treatment group (*n* = 103 compared with *n* = 44 in the placebo group).

Two studies (44, 45) reported on the frequency of nausea without specifying its relation to timing of treatment. Neither study found an increase in reports of nausea with zinc supplementation.

#### Constipation and abdominal pain

Four studies reported on the effect of zinc on constipation (44, 45, 49, 50); 1 found a significant difference between treatment groups (50).

Three trials (41, 44, 45) reported on the occurrence of abdominal pain without specifying its relation to timing of treatment. No significant differences between treatment groups were found.

#### Drowsiness

Two studies (44, 45) reported on the effect of zinc on drowsiness; neither study found a significant increase in reports of drowsiness with zinc supplementation.

#### Mouth irritation and taste aversion

Two studies (44, 45) reported on the frequency of mouth irritation without specifying its relation to timing of treatment. Both studies used a syrup formulation with 15 mg/d elemental zinc as zinc sulfate in 5 mL syrup; neither study found an increase in mouth irritation with zinc supplementation. Three studies (33, 44, 45) reported on taste aversion to zinc syrup, provided as 21 mg/d elemental zinc acetate in 10 mL syrup (33) or 15 mg/d elemental zinc as zinc sulfate in 5 mL syrup (44, 45). Only Brooks et al. (33) reported taste aversion, sometimes leading to regurgitation, and this was highlighted as a potential reason for the higher number of withdrawals in the treatment group (*n* = 103 compared with *n* = 44 in the placebo group) (44, 45).

#### Diarrhea and dysentery

Twenty-seven studies (8, 13, 18, 26, 27, 29–34, 36, 42–45, 49, 50, 55, 56, 58, 59, 61, 63, 67, 72, 75) reported on the effect of zinc on diarrhea, with the majority (*n* = 23) investigating zinc supplementation for the prevention or treatment of diarrhea. Four studies (8, 44, 45, 49) suggested that zinc supplementation could have a potential adverse effect on diarrheal incidence (8, 44, 76). None of the included studies reported a significant increase in or worsening of diarrhea with zinc supplementation.

Three studies (36, 42, 55) reported on the effect of zinc on dysentery or bloody/mucoid diarrhea in children. None of the studies reported a worsening of the condition as a result of zinc supplementation given alone or in combination with iron. Two of these studies reported an improvement in the dysentery, 1 when zinc was provided alone (42) and 1 when zinc was provided at the same time as iron (36).

#### Case studies

Two case studies were identified. Botash et al. (20) reported data from a 6-mo-old infant given a dose of 16–24 mg/d zinc prophylactically. Adverse effects were noted for hematological indices, including serum copper, ceruloplasmin, and serum iron. Sugiura et al. (21) reported on an 11-mo-old infant with atopic dermatitis who consumed 45 mg/d zinc, and recorded adverse effects on serum copper, ceruloplasmin, and hemoglobin.

## Discussion

The setting of ULs for zinc has previously been considered by international panels, including those convened by FAO-WHO (2004) (1), the IOM (77), IZiNCG (7), and the European Food Safety Authority (3). An early step in this process is to collate data from published literature that enable the relation between zinc intake and adverse effects on key outcomes to be described. To date, however, this has been hindered by a lack of data in the 0–3-y age range. Our search identified 62 studies that assessed possible adverse effects of zinc intake in children aged 0–3 y with zinc doses ranging from 3 to 70 mg/d. In most studies doses were <20 mg/d. Data from 39 studies allowed meta-analyses of outcome measures of interest, as identified by the FAO-WHO expert group. Meta-analyses revealed that zinc supplementation had a significant adverse effect on serum ferritin, plasma/serum copper concentration, sTfR, hemoglobin, hematocrit, and the odds of anemia in ≥1 of the subgroups of pooled data. A significant reduction of the lactulose:mannitol ratio was found, indicating improved gut permeability. No significant effects of zinc supplementation on CRP, eSOD, ZPP, blood cholesterol, or iron deficiency anemia were observed in any of the pooled datasets.

Our analyses revealed a significant reduction in serum copper concentration following zinc supplementation in children aged >6 to 12 mo (Figure 14) and >12 mo (Figure 15). Mean reductions were 3.17 and 5.25 μg/dL, respectively. Despite this decrease, the mean serum copper concentration reported in each of the studies remained within the reference range postintervention [children <1 y: 71.16–168.11 μg/dL (78); 0.5–2 y: 72–178 μg/dL (79); and 3–4 y: 80–160 μg/dL (79)], with the exception of 1 study where children were recovering from diarrhea and had low baseline serum copper concentrations (32). This example raises the question about the point at which a change in biochemical outcome measure becomes clinically important, such that the zinc dose that resulted in the change would be considered to pose a risk to health. This can be further explored using dose–response modeling to determine the zinc intake required to result in a clinically significant change in either serum copper concentration or serum ferritin concentration, including the contribution from background dietary zinc intake, in children of various age categories (80). Identifying the threshold value that corresponds to this clinically significant change is also a crucial part of this risk assessment process.

A competitive interaction between zinc and iron during intestinal absorption has been long debated (81), and it has been proposed that high zinc intakes could induce a secondary iron deficiency. In addition to serum ferritin, outcome measures relating to iron status that were included in our meta-analyses included hemoglobin, hematocrit, iron deficiency (measured by plasma ferritin concentration <12 μg/L), iron deficiency anemia (measured by hemoglobin <11 g/dL and plasma ferritin <12 μg/L), serum iron concentration, and sTfR. Meta-analysis of pooled data revealed that hemoglobin concentration was significantly reduced in children aged 91–180 d following zinc doses of 5–10 mg/d (Figure 6), but there was no significant effect on mean values from data pooled by age in the younger or older age categories. Hematocrit and serum iron concentration data were sparse, but analysis of pooled data provided no evidence for a significant effect of zinc supplementation on these outcome measures. Similarly, analysis of the pooled data from studies reporting the risk of iron deficiency anemia and anemia did not reveal any significant effect of zinc supplementation on the OR in any of the age categories for which there were data (Figures 6–8; Table 7). However, combining data from studies with a short duration (<3 mo) did reveal a significant increase in the OR for anemia indicating a possible short-term effect (Table 4).

**TABLE 7:** Summary of forest plot analyses of the effect of zinc supplementation in infants and children aged ≤3 y on iron deficiency (OR), analyzed by duration, chemical formula, and comparator group

		References	Mean difference	95% CI	*P*	*I* ^2^
By treatment duration	0–3 mo	(73)	0.58	0.29, 1.18		NA
	>3 mo	(12, 29, 31, 34, 47, 53, 59, 70, 71)	1.02	0.77, 1.24		29
	Overall		0.98	0.77, 1.24	0.87	32
By chemical formula	Zn gluconate	(73)	0.58	0.29, 1.18		NA
	Zn sulfate	(12, 29, 34, 47, 71)	1.14	0.82, 1.60		34
	Not stated	(31, 53, 70)	1.04	0.80, 1.34		0
	Other	(59)	0.97	0.37, 2.53		NA
	Overall		1.01	0.83, 1.23	0.89	12
Zinc vs. placebo		(12, 29, 53, 59, 70, 71)	1.04	0.79, 1.37	0.79	13

^1^NA, not applicable.

**TABLE 8:** Summary of forest plot analyses of the effect of zinc supplementation in infants and children aged ≤3 y on iron deficiency anemia (OR), analyzed by duration, chemical formula, and comparator group^1^

		References	Mean difference	95% CI	*P*	*I* ^2^
By treatment duration	>3 mo	(29, 34, 39, 47, 71, 74)	1.01	0.73, 1.39	0.97	20
By chemical formula	Zn sulfate	(29, 34, 39, 47, 71)	0.99	0.68, 1.44		34
	Not stated	(74)	1.46	0.42, 5.06		0
	Overall		1.01	0.73, 1.39	0.97	20
Zinc vs. placebo		(29, 39, 71, 74)	0.98	0.67, 1.45	0.93	20
High-dose vs. low-dose		(74)	4.93	0.56, 43.27	0.15	NA

1 NA, not applicable.

**TABLE 9:** Summary of forest plot analyses of the effect of zinc supplementation in infants and children aged ≤3 y on serum/soluble transferrin receptor (mg/L), analyzed by duration, chemical formula, and comparator group^1^

		References	Mean difference	95% CI	*P*	*I* ^2^
By treatment duration	>3 mo	(12, 28, 36, 47, 74)	0.19	0.08, 0.29	<0.001	89
By chemical formula	Zn sulfate	(12, 47)	0.18	0.07, 0.29		97
	Zn acetate	(28)	0.44	−1.08, 1.96		63
	Not stated	(36, 74)	0.26	−0.13, 0.65		0
	Overall		0.19	−0.08, 0.29	<0.001	89
Zinc vs. placebo and (ii) high-dose vs. low-dose zinc		(12, 28, 36, 47, 74)	0.43	−0.16, 0.93	0.08	76
High-dose vs. low-dose		(74)	−0.10	−1.19, 0.99	0.86	NA

1 NA, not applicable.

**TABLE 10:** Summary of forest plot analyses of the effect of zinc supplementation in infants and children aged ≤3 y on hematocrit (proportion), analyzed by duration of intervention, chemical formula, and comparator group^1^

		References	Mean difference	95% CI	*P*	*I* ^2^
By treatment duration	0–3 mo	(51)	−0.01	−0.04, 0.02		NA
	>3 mo	(8, 19, 31, 62, 65)	0.00	−0.01, 0.01		28
	Overall		−0.00	−0.01, 0.01	0.97	17
By chemical formula	Zn gluconate	(62)	0.02	0.00, 0.03		NA
	Zn sulfate	(8, 51, 65)	0.00	−0.01, 0.01		0
	Not stated	(31)	−0.00	−0.00, 0.00		NA
	Other	(19)	−0.01	−0.02, 0.01		NA
	Overall		−0.00	−0.01, 0.01	0.97	17
Zinc vs. placebo		(19, 65)	−0.00	−0.01, 0.01	0.51	0

1 NA, not applicable.

**TABLE 11:** Summary of forest plot analyses of the effect of zinc supplementation in infants and children aged ≤3 y on elevated C-reactive protein (OR), analyzed by duration, chemical formula, and comparator group^1^

		References	Mean difference	95% CI	*P*	*I* ^2^
By treatment duration	>3 mo	(36, 43, 70)	1.19	0.91, 1.55	0.21	0
By chemical formula	Not stated	(36, 43, 70)	1.19	0.91, 1.55	0.21	0
Zinc vs. placebo		(36, 43)	1.05	0.74, 1.48	0.79	0
High-dose vs. low -dose		(43)	0.73	0.37, 1.44	0.36	NA

1 NA, not applicable.

**TABLE 12:** Summary of forest plot analyses of the effect of zinc supplementation in infants and children aged ≤3 y on serum total cholesterol (mg/dL), analyzed by duration, chemical formula, and comparator group^1^

		References	Mean difference	95% CI	*P*	*I* ^2^
By treatment duration	>3 mo	(8, 17, 72)	−0.79	−6.24, 4.66	0.78	0
By chemical formula						
Zn sulfate		(8, 72)	−1.73	−7.70, 4.23		0
	Other	(17)	4.00	−9.45, 17.45		NA
	Overall		0.79	−6.24, 4.66	0.78	0
Zinc vs. placebo		(17, 72)	−1.07	−6.82, 4.69	0.72	0

1 NA, not applicable.

**TABLE 13:** Summary of forest plot analyses of the effect of zinc supplementation in infants and children aged ≤3 y on lactulose:mannitol molar ratio, analyzed by duration, chemical formula, and comparator group^1^

		References	Mean difference	95% CI	*P*	*I* ^2^
By treatment duration	0–3 mo	(60)	−0.08	−0.15, −0.01		NA
	>3 mo	(18)	−0.10	−0.27, 0.07		NA
	Overall		−0.08	−0.14, −0.02	<0.001	0
By chemical formula	Zn gluconate	(18)	−0.10	−0.27, 0.07		NA
	Zn acetate	(60)	−0.08	−0.15, −0.01		NA
	Overall		−0.08	−0.14, −0.12	<0.001	0
Zinc vs. placebo		(18, 60)	−0.08	−0.14, 0.02	0.009	0

1 NA, not applicable.

**TABLE 14:** Summary of forest plot analyses of the effect of zinc supplementation in infants and children aged ≤3 y on serum iron concentration (μg/dL), analyzed by duration, chemical formula, and comparator group^1^

		References	Mean difference	95% CI	*P*	*I* ^2^
By treatment duration	0–3 mo	(51)	−0.12	−11.16, 10.92		NA
	>3 mo	(65)	12.50	10.37, 14.63		NA
	Overall		7.40	−4.74, 19.54	0.23	79
By chemical formula	Zn sulfate	(51, 65)	7.40	−4.74, 19.54	0.23	79
Zinc vs. placebo		(65)	12.50	10.37, 14.63	<0.001	NA

1 NA, not applicable.

Concentrations of sTfR are high when iron deficiency is present, and in situations of increased erythropoietic activity (82). Most of the studies included in the meta-analysis were conducted in children aged 6–12 mo. Analysis of the pooled data indicated a highly significant, detrimental increase of sTfR concentrations. In some studies, children in the intervention but also in the comparison groups (36, 47, 74) had concentrations of sTfR above the reference values for healthy children proposed by some studies (82–84). However, there is a lack of standardization of the methods used to determine sTfR, which limits the comparability of the results and our understanding of the severity of the effect of zinc on sTfR concentrations (82).

The urinary lactulose:mannitol ratio is a biomarker for environmental enteropathy, and its reduction indicates a fall in the gut permeability, which is a desirable outcome from zinc supplementation. Meta-analysis of the data from the 2 studies that reported this outcome showed a statistically significant reduction of the lactulose:mannitol ratio following zinc supplementation in children aged >12 mo. This concurs with previous systematic reviews that have investigated the effectiveness of zinc supplementation as a treatment for diarrhea in children (85).

Doses of zinc ranging from 10 to 20 mg/d in infants aged <12 mo, and from 20 to 30 mg/d for children aged ≥12 mo, increased the risk of vomiting (30, 46, 67, 69) and regurgitation (46, 67, 69) in some studies. From the studies reporting on taste aversion, only 1 study (33), reported it as leading to regurgitation and authors highlighted it as a potential reason for withdrawals among the children in the zinc arm. The studies reporting on nausea, constipation, abdominal pain, mouth irritation, or dysentery did not find an increased incidence of these adverse effects as a result of the zinc doses provided.

Overall, the certainty of the evidence, as assessed using GRADE, was very low to moderate. Factors influencing the downgrading of the evidence included the presence of underlying morbidities and the inclusion of older children in some studies, imprecision in effect estimates due to low numbers and/or heterogeneity, and risk of bias due to randomization processes. Given these limitations in the certainty of the evidence, it is possible that data from further trials could alter the effect estimates summarized here.

### Strengths and limitations

Studies that have collected data on the potential adverse effects of zinc intake in children aged 0–3 y are scarce. As a consequence, it was not possible to conduct meta-analyses by age category, dose, dose duration, and chemical formula of zinc for all the outcomes explored. Additionally, most studies included data from children with relatively low exposures to zinc where adverse effects at such ranges might not be expected. Therefore, it was not possible to identify dose ranges in which zinc might be detrimental for most individuals in the target age group. Nonetheless, data from this review can be used by expert groups to conduct dose–response modeling to establish the tolerable upper intake levels of zinc in children aged 0–3 y.

## Conclusion

Although zinc supplementation at doses of 3 to 20 mg/d had an adverse effect on concentrations of serum/plasma copper, ferritin, hemoglobin, and sTfR in children aged 0–3 y, the change observed might not have a detrimental effect on healthy populations. However, recommended maximum zinc doses might need to be adjusted for children at risk or recovering from iron or copper deficiency. Data from this review can be used to undertake dose–response modeling to estimate tolerable upper intake levels of zinc in children aged 0–3 y. 

## Supplementary Material

nmac088_Supplemental_Files

## Data Availability

Data collection forms, data extracted from included studies, and data used for all analyses are available upon request to the corresponding author. The authors report no conflicts of interest.

## Notes

This systematic review was funded by FAO and WHO.

Author disclosures: The authors report no conflicts of interest. AC and CH are part-funded by the National Institute for Health Research Applied Research Collaboration North West Coast (ARC NWC). The views expressed in this publication are those of the author(s) and not necessarily those of the National Institute for Health Research or the Department of Health and Social Care.

The views expressed in this publication are those of the authors and not necessarily reflect the views or policies of the Food and Agriculture Organization of the United Nations, or the World Health Organization.

Supplemental Files 1–3 are available from the “Supplementary data” link in the online posting of the article and from the same link in the online table of contents at https://academic.oup.com/advances/.

Abbreviations used: CRP, C-reactive protein; eSOD, erythrocyte superoxide dismutase; GRADE, Grading of Recommendations Assessment, Development, and Evaluation; IDA, iron deficiency anemia; IOM, Institute of Medicine; IZiNCG, International Zinc Nutrition Consultative Group; JBI, Joanna Briggs Institute; MD, mean difference; MMN, multiple micronutrients; NA, not applicable; NOAEL, No Observed Adverse Effect Level; PRISMA, Preferred Reporting Items for Systematic Reviews and Meta-Analyses; RCT, randomized controlled trial; RoB, Cochrane Risk of Bias 2; sTfR, serum transferrin receptor; UL, upper limit; ZPP, zinc protoporphyrin.
