# Supplementary material for: Adverse Effects of Excessive Zinc Intake in Infants and Children Aged 0–3 Years: A Systematic Review and Meta-Analysis
Source: Adv Nutr. 2022 Sep 2;13(6):2488–518. doi: 10.1093/advances/nmac088 (PMC9776731; doi:10.1093/advances/nmac088)

**Adverse effects of excessive zinc intake in infants and children aged 0-3 years: A systematic review and meta-analysis.**

Supplementary File 1: Search Strategy

Contents

[Original Search strategy 1](#_Toc106008791)

[Cochrane 1](#_Toc106008792)

[Medline 4](#_Toc106008793)

[Embase 6](#_Toc106008794)

[Additional expanded Search post WHO Panel Meeting 7](#_Toc106008795)

[Cochrane 7](#_Toc106008796)

[Medline 10](#_Toc106008797)

[Embase 11](#_Toc106008798)

[Additional search filtering methods and results 13](#_Toc106008799)

[Step 1 13](#_Toc106008800)

[Step 2 14](#_Toc106008801)

[Figure 1 15](#_Toc106008802)

[Figure 2 16](#_Toc106008803)

# Original Search strategy

This Search Strategy includes terms relating to toxicity and adverse effects

## Cochrane

Cochrane Database of Systematic Reviews and Cochrane Central Register of Controlled Trials (CENTRAL) in the Cochrane Library

Date searched: 07/08/2020

| #1 | MeSH descriptor: [Zinc] explode all trees |
| --- | --- |
| #2 | MeSH descriptor: [Zinc Compounds] explode all trees |
| #3 | MeSH descriptor: [Zinc Acetate] explode all trees |
| #4 | zinc or zn |
| #5 | {OR #1-#4} |
| #6 | MeSH descriptor: [Drug-Related Side Effects and Adverse Reactions] explode all trees |
| #7 | MeSH descriptor: [Maximum Allowable Concentration] explode all trees |
| #8 | MeSH descriptor: [Drug Overdose] explode all trees |
| #9 | MeSH descriptor: [Zinc] explode all trees and with qualifier(s): [adverse effects - AE] |
| #10 | MeSH descriptor: [Zinc Compounds] explode all trees and with qualifier(s): [adverse effects - AE] |
| #11 | MeSH descriptor: [Zinc Acetate] explode all trees and with qualifier(s): [adverse effects - AE] |
| #12 | MeSH descriptor: [Zinc] explode all trees and with qualifier(s): [toxicity - TO] |
| #13 | MeSH descriptor: [Zinc Compounds] explode all trees and with qualifier(s): [toxicity - TO] |
| #14 | MeSH descriptor: [Zinc Acetate] explode all trees and with qualifier(s): [toxicity - TO] |
| #15 | (zinc or zn) NEAR/9 (adverse* or danger* or harmful or indirect or injurious or secondary or side or undesirable) NEAR/1 (complicat* or consequence* or effect* or affect* or event* or impact* or outcome* or react*) |
| #16 | (zinc or zn) NEAR/9 (excess* or too much or toxic* or exceed* or interact* or contraindicat* or poison* or overdos* or oversupp* or (over NEXT supp*)) |
| #17 | (zinc or zn) NEAR/9 (high* or max* or upper or safe or unsafe) NEAR/3 (limit* or level* or intake or dose* or dosing) |
| #18 | {OR #6-#17} |
| #19 | MeSH descriptor: [Absorption] explode all trees |
| #20 | (absorb* or absorption or status or deficien* or interact*) NEAR/4 (mineral* or nutrient* or micronutrient* or (trace NEXT metal*) or iron or fe or copper or cu or magnesium or Ca or mg or ca or tin or sn or phytate* or "phytic acid" or "inositol hexakisphosphate" or "inositol polyphosphate" or ip6) |
| #21 | MeSH descriptor: [Phytic Acid] explode all trees |
| #22 | MeSH descriptor: [Hemoglobins] explode all trees |
| #23 | MeSH descriptor: [Ferritins] explode all trees |
| #24 | MeSH descriptor: [Anemia, Iron-Deficiency] explode all trees |
| #25 | MeSH descriptor: [Anemia] explode all trees |
| #26 | h?emoglobin* or ferritin* or an?emi* |
| #27 | MeSH descriptor: [Lipids] explode all trees and with qualifier(s): [blood - BL] |
| #28 | MeSH descriptor: [Cholesterol] explode all trees and with qualifier(s): [blood - BL] |
| #29 | MeSH descriptor: [Triglycerides] explode all trees and with qualifier(s): [blood - BL] |
| #30 | MeSH descriptor: [Fatty Acids] explode all trees and with qualifier(s): [blood - BL] |
| #31 | MeSH descriptor: [Lipid Metabolism] explode all trees and with qualifier(s): [drug effects - DE] |
| #32 | MeSH descriptor: [Lipoproteins] explode all trees and with qualifier(s): [blood - BL] |
| #33 | (blood or serum or plasma) NEAR/2 (lipid* or fat?) |
| #34 | "lipid status" or cholesterol or triglyceride or lipoprotein* or triacylglycerol or hyperlipidemia or hypercholesterol?emia or hypocholesterol?emia |
| #35 | MeSH descriptor: [Gastrointestinal Tract] explode all trees |
| #36 | MeSH descriptor: [Gastrointestinal Diseases] explode all trees |
| #37 | MeSH descriptor: [Gastrointestinal Microbiome] explode all trees |
| #38 | (gastrointestinal or digestive or intestinal or gut or gastric or stomach or genitourinary or urolog* or urinary or gastroduodenal) NEAR/3 (system or tract or function* or health or microbiome or Microbiota or disorder* or disease* or bleed* or irritation or damag* or pain or injur*) |
| #39 | nausea or vomit* or diarrh?ea or headache* |
| #40 | MeSH descriptor: [Immunity] explode all trees |
| #41 | immune or immunity or immunodeficien* or leukopenia or neutropenia or "oxidative stress" |
| #42 | MeSH descriptor: [DNA Damage] explode all trees |
| #43 | (dna or "deoxyr.ibonucleic acid") NEAR/2 (damag* or break* or fragment*) |
| #44 | apoptosis or "cell death" or comet |
| #45 | {OR #19-#44} |
| #46 | #5 AND #18 AND #45 |

## Medline

Ovid MEDLINE(R) and Epub Ahead of Print, In-Process & Other Non-Indexed Citations and Daily 1946 to August 05, 2020

Date searched: 07/08/2020

| 1 | exp Zinc/ |
| --- | --- |
| 2 | exp Zinc Compounds/ or Zinc Acetate/ |
| 3 | (zinc or zn).ti,kf,kw. |
| 4 | (zinc or zn).ab. /freq=2 |
| 5 | 1 or 2 or 3 or 4 |
| 6 | exp "Drug-Related Side Effects and Adverse Reactions"/ or Maximum Allowable Concentration/ or Drug Overdose/ |
| 7 | exp Zinc/ae or exp Zinc Compounds/ae or Zinc Acetate/ae |
| 8 | exp Zinc/to or exp Zinc Compounds/to or Zinc Acetate/to |
| 9 | ((zinc or zn) adj9 (adverse* or danger* or harmful or indirect or injurious or secondary or side or undesirable) adj1 (complicat* or consequence* or effect* or affect* or event* or impact* or outcome* or react*)).tw. |
| 10 | ((zinc or zn) adj9 (excess* or too much or toxic* or exceed* or interact* or contraindicat* or poison* or overdos* or oversupp* or over supp*)).tw. |
| 11 | ((zinc or zn) adj9 (high* or max* or upper or safe or unsafe) adj3 (limit* or level* or intake or dose* or dosing)).tw. |
| 12 | or/6-11 |
| 13 | Absorption/ |
| 14 | ((absorb* or absorption or status or deficien* or interact*) adj4 (mineral* or nutrient* or micronutrient* or trace metal* or iron or fe or copper or cu or magnesium or Ca or mg or ca or tin or sn or phytate* or phytic acid* or inositol hexakisphosphate or inositol polyphosphate or ip6)).tw. |
| 15 | Phytic Acid/ |
| 16 | Hemoglobins/ |
| 17 | Ferritins/ |
| 18 | Anemia, Iron-Deficiency/ or Anemia/ |
| 19 | (h?emoglobin* or ferritin* or an?emi*).tw. |
| 20 | Lipids/bl [Blood] |
| 21 | Cholesterol/bl [Blood] |
| 22 | Triglycerides/bl [Blood] |
| 23 | Fatty Acids/bl [Blood] |
| 24 | Lipid Metabolism/de [Drug Effects] |
| 25 | exp Lipoproteins/bl [Blood] |
| 26 | ((blood or serum or plasma) adj2 (lipid* or fat ±1)).tw. |
| 27 | (lipid status or cholesterol or triglyceride or lipoprotein* or triacylglycerol or hyperlipidemia or hypercholesterol?emia or hypocholesterol?emia).tw. |
| 28 | Gastrointestinal Tract/ or exp Gastrointestinal Diseases/ |
| 29 | Gastrointestinal Microbiome/ |
| 30 | ((gastrointestinal or digestive or intestinal or gut or gastric or stomach or genitourinary or urolog* or urinary or gastroduodenal) adj3 (system or tract or function* or health or microbiome or Microbiota or disorder* or disease* or bleed* or irritation or damag* or pain or injur*)).tw. |
| 31 | (nausea or vomit* or diarrh?ea or headache*).tw. |
| 32 | Immunity/ |
| 33 | (immune or immunity or immunodeficien* or leukopenia or neutropenia or oxidative stress).tw. |
| 34 | exp DNA Damage/ |
| 35 | ((dna or deoxyr.ibonucleic acid) adj2 (damag* or break* or fragment*)).tw. |
| 36 | (apoptosis or cell death or comet).tw. |
| 37 | or/13-36 |
| 38 | 5 and 12 and 37 |
| 39 | exp Animals/ not humans/ |
| 40 | 38 not 39 |

## Embase

Embase (via Ovid) 1974 to 2020 August 06

Date searched: 07/08/2020

| 1 | exp zinc/ or zinc derivative/ or zinc oxide/ or zinc sulfate/ or zinc acetate/ |
| --- | --- |
| 2 | (zinc or zn).ti,kw. |
| 3 | (zinc or zn).ab. /freq=2 |
| 4 | 1 or 2 or 3 |
| 5 | exp adverse event/ |
| 6 | intoxication/ or drug overdose/ |
| 7 | maximum allowable concentration/ |
| 8 | exp zinc/ae or zinc derivative/ae or zinc oxide/ae or zinc sulfate/ae or zinc acetate/ae |
| 9 | exp zinc/to or zinc derivative/to or zinc oxide/to or zinc sulfate/to or zinc acetate/to |
| 10 | ((zinc or zn) adj9 (adverse* or danger* or harmful or indirect or injurious or secondary or side or undesirable) adj1 (complicat* or consequence* or effect* or affect* or event* or impact* or outcome* or react*)).tw. |
| 11 | ((zinc or zn) adj9 (excess* or too much or toxic* or exceed* or interact* or contraindicat* or poison* or overdos* or oversupp* or over supp*)).tw. |
| 12 | ((zinc or zn) adj9 (high* or max* or upper or safe or unsafe) adj3 (limit* or level* or intake or dose* or dosing)).tw. |
| 13 | or/5-12 |
| 14 | absorption/ |
| 15 | ((absorb* or absorption or status or deficien* or interact*) adj4 (mineral* or nutrient* or micronutrient* or trace metal* or iron or fe or copper or cu or magnesium or Ca or mg or ca or tin or sn or phytate* or phytic acid* or inositol hexakisphosphate or inositol polyphosphate or ip6)).tw. |
| 16 | phytic acid/ |
| 17 | hemoglobin/ |
| 18 | ferritin/ |
| 19 | anemia/ or iron deficiency anemia/ |
| 20 | (h?emoglobin* or ferritin* or an?emi*).tw. |
| 21 | lipid blood level/ |
| 22 | cholesterol blood level/ |
| 23 | triacylglycerol blood level/ |
| 24 | fatty acid blood level/ |
| 25 | lipid metabolism/ |
| 26 | lipoprotein blood level/ |
| 27 | ((blood or serum or plasma) adj2 (lipid* or fat ±1)).tw. |
| 28 | (lipid status or cholesterol or triglyceride or lipoprotein* or triacylglycerol or hyperlipidemia or hypercholesterol?emia or hypocholesterol?emia).tw. |
| 29 | gastrointestinal tract/ |
| 30 | exp gastrointestinal disease/ |
| 31 | intestine flora/ |
| 32 | ((gastrointestinal or digestive or intestinal or gut or gastric or stomach or genitourinary or urolog* or urinary or gastroduodenal) adj3 (system or tract or function* or health or microbiome or Microbiota or disorder* or disease* or bleed* or irritation or damag* or pain or injur*)).tw. |
| 33 | (nausea or vomit* or diarrh?ea or headache*).tw. |
| 34 | immunity/ |
| 35 | (immune or immunity or immunodeficien* or leukopenia or neutropenia or oxidative stress).tw. |
| 36 | exp DNA damage/ |
| 37 | ((dna or deoxyr.ibonucleic acid) adj2 (damag* or break* or fragment*)).tw. |
| 38 | (apoptosis or cell death or comet).tw. |
| 39 | or/14-38 |
| 40 | 4 and 13 and 39 |
| 41 | exp animal/ not human/ |
| 42 | 40 not 41 |

# Additional expanded Search post WHO Panel Meeting

Search Strategy that excludes terms relating to toxicity and adverse effects

## Cochrane

Database: Cochrane

Date searched: 07/08/2020

| **Search Number** | **Query** | **Hits** |
| --- | --- | --- |
| #1 | MeSH descriptor: [Zinc] explode all trees | 1664 |
| #2 | MeSH descriptor: [Zinc Compounds] explode all trees | 547 |
| #3 | MeSH descriptor: [Zinc Acetate] explode all trees | 26 |
| #4 | zinc or zn | 8913 |
| #5 | {OR #1-#4} | 8913 |
| #6 | MeSH descriptor: [Absorption] explode all trees | 3382 |
| #7 | (absorb* or absorption or status or deficien* or interact*) NEAR/4 (mineral* or nutrient* or micronutrient* or (trace NEXT metal*) or iron or fe or copper or cu or magnesium or calcium or mg or ca or tin or sn or phytate* or "phytic acid" or "inositol hexakisphosphate" or "inositol polyphosphate" or ip6) | 9209 |
| #8 | MeSH descriptor: [Phytic Acid] explode all trees | 105 |
| #9 | MeSH descriptor: [Hemoglobins] explode all trees | 9582 |
| #10 | MeSH descriptor: [Ferritins] explode all trees | 1035 |
| #11 | MeSH descriptor: [Anemia, Iron-Deficiency] explode all trees | 1360 |
| #12 | MeSH descriptor: [Anemia] explode all trees | 5425 |
| #13 | h?emoglobin* or ferritin* or an?emi* | 141646 |
| #14 | MeSH descriptor: [Lipids] explode all trees and with qualifier(s): [blood - BL] | 20282 |
| #15 | MeSH descriptor: [Cholesterol] explode all trees and with qualifier(s): [blood - BL] | 9383 |
| #16 | MeSH descriptor: [Triglycerides] explode all trees and with qualifier(s): [blood - BL] | 5664 |
| #17 | MeSH descriptor: [Fatty Acids] explode all trees and with qualifier(s): [blood - BL] | 4348 |
| #18 | MeSH descriptor: [Lipid Metabolism] explode all trees and with qualifier(s): [drug effects - DE] | 796 |
| #19 | MeSH descriptor: [Lipoproteins] explode all trees and with qualifier(s): [blood - BL] | 8555 |
| #20 | (blood or serum or plasma) NEAR/2 (lipid* or fat?) | 14680 |
| #21 | lipid status or cholesterol or triglyceride or lipoprotein* or triacylglycerol or hyperlipidemia or hypercholesterol?emia or hypocholesterol?emia | 51650 |
| #22 | MeSH descriptor: [Gastrointestinal Tract] explode all trees | 11922 |
| #23 | MeSH descriptor: [Gastrointestinal Diseases] explode all trees | 35352 |
| #24 | MeSH descriptor: [Gastrointestinal Microbiome] explode all trees | 605 |
| #25 | (gastrointestinal or digestive or intestinal or gut or gastric or stomach or genitourinary or urolog* or urinary or gastroduodenal) NEAR/3 (system or tract or function* or health or microbiome or Microbiota or disorder* or disease* or bleed* or irritation or damag* or pain or injur*) | 55321 |
| #26 | nausea or vomit* or diarrh?ea or headache* | 94375 |
| #27 | MeSH descriptor: [Immunity] explode all trees | 4036 |
| #28 | immune or immunity or immunodeficien* or leukopenia or neutropenia or "oxidative stress" | 79192 |
| #29 | MeSH descriptor: [DNA Damage] explode all trees | 451 |
| #30 | (dna or "deoxyribonucleic acid") NEAR/2 (damag* or break* or fragment*) | 1817 |
| #31 | apoptosis or "cell death" or comet | 5848 |
| #32 | {OR #6-#31} | 392090 |
| #33 | #5 AND #32 | 3933 |
|  | Review, protocols, RCTs only | 3905 |

## Medline

Ovid MEDLINE(R) and Epub Ahead of Print, In-Process, In-Data-Review & Other Non-Indexed Citations and Daily 1946 to June 10, 2021

Date searched: 11/06/2021

| **Search Number** | **Query** | **Hits** |
| --- | --- | --- |
| 1 | exp Zinc/ | 61897 |
| 2 | exp Zinc Compounds/ or Zinc Acetate/ | 14004 |
| 3 | (zinc or zn).ti,kf,kw. | 66473 |
| 4 | (zinc or zn).ab. /freq=2 | 78346 |
| 5 | 1 or 2 or 3 or 4 | 129394 |
| 6 | Absorption/ | 29821 |
| 7 | ((absorb* or absorption or status or deficien* or interact*) adj4 (mineral* or nutrient* or micronutrient* or trace metal* or iron or fe or copper or cu or magnesium or calcium or mg or ca or tin or sn or phytate* or phytic acid* or inositol hexakisphosphate or inositol polyphosphate or ip6)).tw. | 87189 |
| 8 | Phytic Acid/ | 3451 |
| 9 | Hemoglobins/ | 69910 |
| 10 | Ferritins/ | 20089 |
| 11 | Anemia, Iron-Deficiency/ or Anemia/ | 61040 |
| 12 | (h?emoglobin* or ferritin* or an?emi*).tw. | 321048 |
| 13 | Lipids/bl [Blood] | 52121 |
| 14 | Cholesterol/bl [Blood] | 65298 |
| 15 | Triglycerides/bl [Blood] | 50720 |
| 16 | Fatty Acids/bl [Blood] | 7327 |
| 17 | Lipid Metabolism/de [Drug Effects] | 9603 |
| 18 | exp Lipoproteins/bl [Blood] | 78246 |
| 19 | ((blood or serum or plasma) adj2 (lipid* or fat$1)).tw. | 55021 |
| 20 | (lipid status or cholesterol or triglyceride or lipoprotein* or triacylglycerol or hyperlipidemia or hypercholesterol?emia or hypocholesterol?emia).tw. | 372873 |
| 21 | Gastrointestinal Tract/ or exp Gastrointestinal Diseases/ | 1009492 |
| 22 | Gastrointestinal Microbiome/ | 21961 |
| 23 | ((gastrointestinal or digestive or intestinal or gut or gastric or stomach or genitourinary or urolog* or urinary or gastroduodenal) adj3 (system or tract or function* or health or microbiome or Microbiota or disorder* or disease* or bleed* or irritation or damag* or pain or injur*)).tw. | 340405 |
| 24 | (nausea or vomit* or diarrh?ea or headache*).tw. | 258888 |
| 25 | Immunity/ | 27146 |
| 26 | (immune or immunity or immunodeficien* or leukopenia or neutropenia or oxidative stress).tw. | 1136822 |
| 27 | exp DNA Damage/ | 100589 |
| 28 | ((dna or deoxyribonucleic acid) adj2 (damag* or break* or fragment*)).tw. | 153254 |
| 29 | (apoptosis or cell death or comet).tw. | 503347 |
| 30 | or/6-29 | 3824489 |
| 31 | 5 and 30 | 21188 |
| 32 | exp Animals/ not humans/ | 4840351 |
| 33 | 31 not 32 | 15080 |

## Embase

Embase (via Ovid) 1974 to 2021 June 10

Date searched: 11/06/2021

| **Search Number** | **Query** | **Hits** |
| --- | --- | --- |
| 1 | exp zinc/ or zinc derivative/ or zinc oxide/ or zinc sulfate/ or zinc acetate/ | 140130 |
| 2 | (zinc or zn).ti,kw. | 76649 |
| 3 | (zinc or zn).ab. /freq=2 | 92736 |
| 4 | 1 or 2 or 3 | 185508 |
| 5 | absorption/ | 51599 |
| 6 | ((absorb* or absorption or status or deficien* or interact*) adj4 (mineral* or nutrient* or micronutrient* or trace metal* or iron or fe or copper or cu or magnesium or calcium or mg or ca or tin or sn or phytate* or phytic acid* or inositol hexakisphosphate or inositol polyphosphate or ip6)).tw. | 111414 |
| 7 | phytic acid/ | 3851 |
| 8 | hemoglobin/ | 196713 |
| 9 | ferritin/ | 49648 |
| 10 | anemia/ or iron deficiency anemia/ | 214762 |
| 11 | (h?emoglobin* or ferritin* or an?emi*).tw. | 444290 |
| 12 | lipid blood level/ | 49885 |
| 13 | cholesterol blood level/ | 135786 |
| 14 | triacylglycerol blood level/ | 72231 |
| 15 | fatty acid blood level/ | 13050 |
| 16 | lipid metabolism/ | 81600 |
| 17 | lipoprotein blood level/ | 23893 |
| 18 | ((blood or serum or plasma) adj2 (lipid* or fat$1)).tw. | 71108 |
| 19 | (lipid status or cholesterol or triglyceride or lipoprotein* or triacylglycerol or hyperlipidemia or hypercholesterol?emia or hypocholesterol?emia).tw. | 489986 |
| 20 | gastrointestinal tract/ | 57909 |
| 21 | exp gastrointestinal disease/ | 91598 |
| 22 | intestine flora/ | 64549 |
| 23 | ((gastrointestinal or digestive or intestinal or gut or gastric or stomach or genitourinary or urolog* or urinary or gastroduodenal) adj3 (system or tract or function* or health or microbiome or Microbiota or disorder* or disease* or bleed* or irritation or damag* or pain or injur*)).tw. | 464951 |
| 24 | (nausea or vomit* or diarrh?ea or headache*).tw. | 402445 |
| 25 | immunity/ | 69689 |
| 26 | (immune or immunity or immunodeficien* or leukopenia or neutropenia or oxidative stress).tw. | 1501697 |
| 27 | exp DNA damage/ | 154609 |
| 28 | ((dna or deoxyribonucleic acid) adj2 (damag* or break* or fragment*)).tw. | 183978 |
| 29 | (apoptosis or cell death or comet).tw. | 655504 |
| 30 | or/5-29 | 4185763 |
| 31 | 4 and 30 | 33592 |
| 32 | (exp animal/ or animal.hw. or nonhuman/) not (exp human/ or human cell/ or (human or humans).ti.) | 6618878 |
| 33 | 31 not 32 | 22906 |

# Additional search filtering methods and results

## Step 1

(See Figure 1)

1. We re-run the searches for SR2 without the terms for adverse effects (lines 6-12 in the original search). This search generated 41, 891 results.
2. We ran the automatic de-duplication.
3. Using the “VLOOKUP exact” function in excel, we compared the Title of the studies included in the previous search (N=4,970) with the new search. This step identified N=6,646 studies that had the exact title. We transferred the N=6,646 to another excel sheet and removed duplicated titles (N=2040), finding that we identified N=4606 studies that were in the previous search. It is possible that the remaining (N=364) studies to complete the number of studies screened in the first search (N=4,970) were not identified with the “VLOOKUP exact” function. Therefore, these may be in the “new studies” category.
4. We then used the ISNUMBER function in excel to identify the key words of our outcomes of interest in the Abstract or titles of the studies. These could be used to filter out those studies that may not include any of the outcomes of interest. A total of N=14,325 studies are “New” and include at least one of the “terms” of the 6 main outcomes of interest in their abstract.

Table 1 shows the terms used to identify in the potential studies.

Table 1 Key to filter studies on main outcomes

**Haemoglobin:** Hb, Haemoglobin, Hemoglobin *(Main 6)*

**Serum/Plasma copper:**  Cu, Copper *(Main 6)*

**Haematocrit:** Hct, Haematocrit, hematocrit, PCV

**Ferritin:** FER, ferritin *(Main 6)*

**Serum/soluble transferrin receptor:** transferrin, stfr, tfr *(Main 6)*

**Iron deficiency:** Fe, Iron *(Main 6)*

**Anaemia:** Anaemia, Anemia *(Main 6)*

**Iron deficiency anemia:** IDA (Main 6)

**Zn protoporphyrin:** protoporphyrin

**Erythrocyte SOD:** Erythrocyte SOD, eSOD

**Raised CRP:** CRP

*Note:* The ISNUMBER function recognizes the word regardless if it is Caps Lock or not, and it also recognize a character within another word, for example it would recognize child in children. Therefore, we used spaces between the words: “ Cu “, “ fer “, “ IDA “ and “ Fe “ to avoid identifying these terms in non-relevant words.

1. Then the ISNUMBER function and key terms (Table 2) were used to filter those studies that could potentially have children in our age of interest. A total of N= 3,852 studies had a term for the age of interest and include a term of the 6 main outcomes

Table 2Key terms to filter studies on age of interest

child, school, infant, girl, boy, toddler, neonate, newborn, new-born, babies, baby

1. We the used the ISNUMBER function and key terms (Table 3) to filter those studies that could be RCT or quasi experimental studies.

Table 3Key terms to identify studies with the study design of interest

RCT, randomised, randomized, assigned, allocated, randomly, group

1. In a second excel sheet we de-duplicated studies by title to have an estimate how many of these studies were duplicated (N=667). A total of 1,410 studies include a term for the main 6 outcomes, age of interest and study design.

## Step 2

(See Figure 2)

1. Following revision of the terms used to filter the studies. We identified two more terms: “**Anemic”** and **“controlled”** that could potentially help us to identify relevant studies. The terms were search on the dataset of studies that were filtered out in Step 1, adding 29 articles for the total number of studies (N=1439) reviewed for Full text and abstract.
2. From the studies reviewed for full text and abstract N=1377 were not relevant.
3. 15 studies were duplicated with the first search but not filtered out through the automatic de-duplication. Two studies were excluded because these were studies published after September 2020 when our initial search took place.
4. 45 studies were included for full text screening, from which 32 were excluded.
5. 2 Additional studies were identified. One study through a conference abstract excluded in this additional search, and one study through revision of the excluded studies from the original title and abstract screening.
6. A total of N=15 studies were included.

## Figure 1


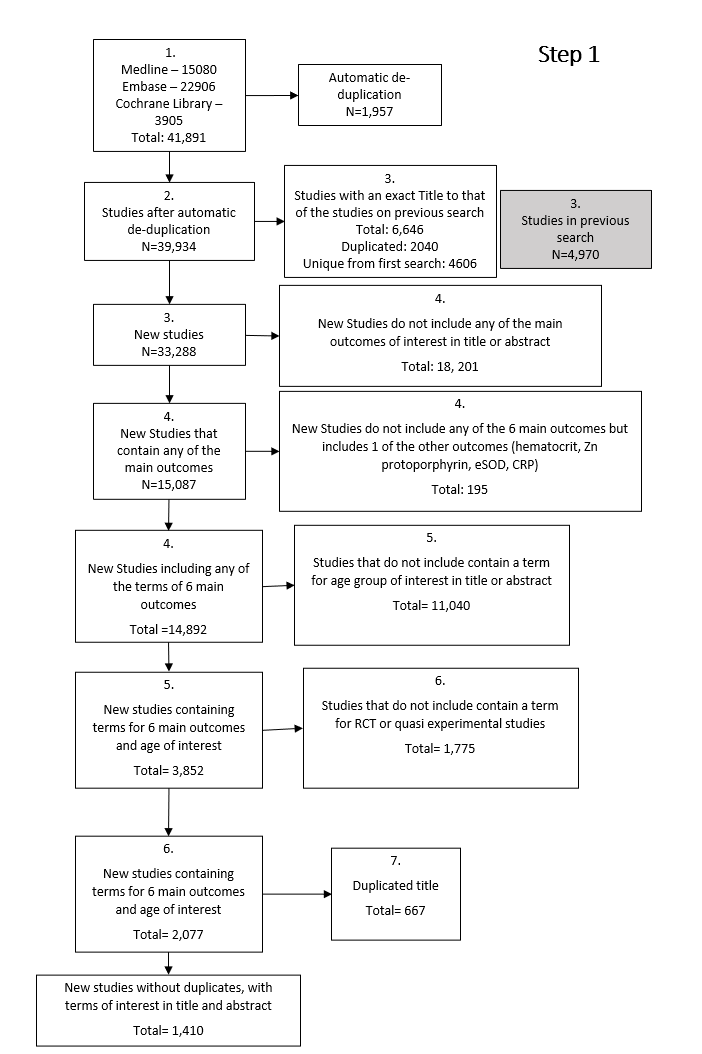


## Figure 2


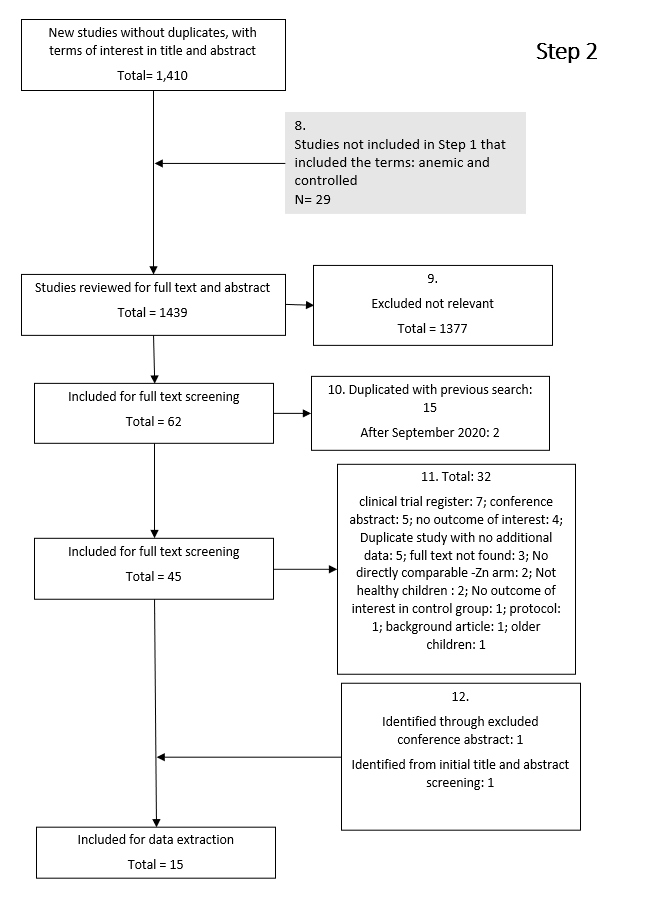

Supplement: nmac088_Supplemental_Files [file nmac088_supplemental_files.zip › Supplementary file 1 Original Search strategy.docx]
