# Supplementary material for: Adverse Effects of Excessive Zinc Intake in Infants and Children Aged 0–3 Years: A Systematic Review and Meta-Analysis
Source: Adv Nutr. 2022 Sep 2;13(6):2488–518. doi: 10.1093/advances/nmac088 (PMC9776731; doi:10.1093/advances/nmac088)
Supplement: nmac088_Supplemental_Files [file nmac088_supplemental_files.zip › Supplementary File 2 Additional Tables.docx]

**Adverse effects of excessive zinc intake in infants and children aged 0-3 years: A systematic review and meta-analysis.**

Supplementary File 2. Additional tables

Contents

[**Table 1** Overview of the studies included in the review 2](#_Toc106009061)

[**Table 2** Dietary intake of arms included in the meta-analyses 19](#_Toc106009062)

[References 23](#_Toc106009063)

**Note:** Reference as per the reference list at the end of this document.

# **Table 1** Overview of the studies included in the review

| **Author surname, year (Country)  Synthesis: N narrative, M meta-analysis** | **Study design** | **Zinc exposure (unit)** | **Duration of exposure (days/months/ years)** | **Form of exposure (supplement, diet, etc.)** | **Outcome measures reported** | **Evidence for adverse impact (yes/no) (for each outcome measure)** |
| --- | --- | --- | --- | --- | --- | --- |
| Abbeddou et al. (1)  (Burkina Faso) ^[[1]](#footnote-2)^ Synthesis: N & M | RCT (cluster randomized, partially double blinded): 2435 children aged 9 mo. of age were assigned to the intervention cohort (1) small quantity lipid based nutrient supplement [SQ-LNS] without added Zn + placebo tablet; 2) SQ-LNS with 5 mg added Zn + placebo tablet; 3) SQ-LNS with 10 mg added Zn + placebo tablet; 4) SQ-LNS without added Zn + 5 mg Zn tablet) until 18 mo. of age and 785 children were assigned to the non-intervention group who did not receive SQ-LNS or tablets from 9 to 18 mo. of age, but received SQ-LNS with 10 mg zinc for 9 mo. beginning at 18 mo. of age after the final blood sample was collected. Randomized at 2 levels: 1) the community and 2) the concession (extended family compound). | 5 mg/d  10 mg/d | 9 months | Lipid-based nutrient supplement | · Hemoglobin | · No |
|  |  |  |  |  | · Serum ferritin | · Yes (10 mg/d) |
|  |  |  |  |  | · Soluble transferrin receptor | · No |
|  |  |  |  |  | · Body iron stores | · Yes (10 mg/d) |
|  |  |  |  |  | · Zn protoporphyrin | · No |
|  |  |  |  |  | · Anemia | · No |
|  |  |  |  |  | · Iron deficiency anemia | · No |
|  |  |  |  |  | · Malaria | · No |
|  |  |  |  |  | · Fever | · No |
|  |  |  |  |  | · Diarrhea | · No |
| Abdollahi et al. (2)  (Iran)  Synthesis: N & M | RCT (multicenter cluster randomized, double blind, placebo-controlled effectiveness trial): 682 children aged 6-24 mo. were assigned to 5 mg elemental Zn/d (n =272) or placebo (n = 308) for 6 mo. | 5 mg/d | 6 months | Supplement | · General adverse effects | · No |
|  |  |  |  |  | · Serum Ferritin | · No |
|  |  |  |  |  | · Diarrhea | · No |
|  |  |  |  |  | · Respiratory diseases | · No |
| Alarcon et al. (3)  (Peru)  Synthesis: N & M | RCT (double blind, placebo controlled): 335 anemic children aged 6-35 mo. were assigned to 1) Fe, 2) Fe + Zn, or 3) Fe + Zn + Vit A (one off dose of vit A/placebo) for 18 wks. Zn/placebo + Fe provided >= 1 h apart 6d/wk | 0.7 mg/kg/d (~7.3 mg/d) | 18 weeks | Supplement | · Hemoglobin | · No |
|  |  |  |  |  | · Serum ferritin | · No |
|  |  |  |  |  | · Diarrhea | · No |
| Awasthi et al. (4)  (Brazil, India (2 sites), Egypt, Ethiopia, Philippines)  Synthesis: N | RCT (open label, non-placebo controlled, effectiveness trial): ORS vs ORS + 14 days of zinc (with culturally appropriate messages for zinc usage) for acute diarrhea in children aged 2-59 mo. | 10 mg/d  20 mg/d | 14 days | Supplement | · Vomiting on days 3-5 | · No |
|  |  |  |  |  | · Vomiting on days 15-17 | · No |
|  |  |  |  |  | · Diarrhea on days 3-5 | · No |
|  |  |  |  |  | · ORS use day 3 | · No |
| Baqui et al. (5)  (Bangladesh)  Synthesis: N & M | RCT (double blind, placebo controlled): 6-mo-old infants were assigned to receive weekly supplements of 1 mg riboflavin (control, n= 82) or 1 mg riboflavin + 20 mg iron (n =83), 20 mg zinc (n =83), or both (n =85) for 6 mo. Hemoglobin, serum ferritin, transferrin receptor, zinc, and copper concentrations were measured at baseline and at the end of intervention. | 20 mg (weekly) | 6 months | Supplement | · Hemoglobin | · No |
|  |  |  |  |  | · Serum Ferritin | · No |
|  |  |  |  |  | · Serum transferrin receptor | · No |
|  |  |  |  |  | · Serum copper | · Yes |
| Bates et al. (6)  (Gambia)  Synthesis: N & M | Controlled trial (double blind): 110 rural Gambian children aged between 0.57 and 2.30 years were divided into two matched groups, 70 mg Zn twice weekly for 1.25 years, or placebo. | 70 mg (twice weekly) | 1.25 years | Supplement (given in fruit drink) | · Hemoglobin | · No |
|  |  |  |  |  | · Serum Ferritin | · No |
|  |  |  |  |  | · CD4 lymphocytes, CD8 lymphocytes, ratio | · No |
|  |  |  |  |  | · C3 complement | · No |
|  |  |  |  |  | · Secretory IgA (urine) | · No |
|  |  |  |  |  | · Erythrocyte superoxide dismutase | · No |
|  |  |  |  |  | · Hair Cu | · No |
|  |  |  |  |  | · Lactulose, mannitol, creatinine ratios | · No |
|  |  |  |  |  | · Diarrhea | · No |
|  |  |  |  |  | · Malaria | · No |
|  |  |  |  |  | · Respiratory infection | · No |
| Berger et al. (7)  (Vietnam)  Synthesis: N & M | RCT (double blind, placebo controlled): 915 rural Vietnamese breastfed infants aged 4-6 mo. were assigned to 10 mg of iron, 10 mg of zinc, 10 mg of iron + 10 mg of zinc or placebo for 6 mo. Hemoglobin (Hb), serum ferritin (SF) and zinc (SZn), and anthropometry were measured before and at the end of the intervention. Morbidity was recorded daily. | 10 mg/d | 6 months | Supplement | · Hemoglobin | · No |
|  |  |  |  |  | · Serum Ferritin | · No |
|  |  |  |  |  | · Anemia | · No |
|  |  |  |  |  | · Iron deficiency anemia | · No |
|  |  |  |  |  | · Acute respiratory infection | · No |
|  |  |  |  |  | · Cough | · No |
|  |  |  |  |  | · Fever | · No |
|  |  |  |  |  | · Diarrhea | · No |
| Bhandari et al. (8)  (India)  Synthesis: N & M | RCT (double blind, placebo-controlled): infants & young children aged 6-30 mo. were randomized to receive placebo or elemental Zn (10 mg to infants and 20 mg to older children) for 4 mo. and incidence of severe & recurrent diarrhea recorded | Infants: 10 mg/d  Older children (up to 30 mo.): 20 mg/d | 4 months | Supplement | · Vomiting | · Yes |
|  |  |  |  |  | · Plasma Cu | · Yes |
|  |  |  |  |  | · Diarrhea | · No |
| Bhandari et al. (9)  (India)  Synthesis: N & M | RCT (double blind, cluster randomized at household level): 94,359 subjects aged 1–23 mo. were administered a daily dose of 10 mg zinc plus iron folic acid (IFA) or IFA alone for a duration of 12 mo. after enrolment. | Infants <6 mo.: 5 mg/d  Infants and children ≥ 6 mo.: 10 mg/d | 12 months | Supplement | · Plasma ferritin  · Iron deficiency | · No  · No |
|  |  |  |  |  | · Iron deficiency | · No |
|  |  |  |  |  | · Hematocrit | · No |
|  |  |  |  |  | · Hospitalizations for pneumonia, diarrhea | · No |
|  |  |  |  |  | · Death | · No |
| Bhutta et al. (10)  (Pakistan)  Synthesis: N & M | RCT (double blind, placebo controlled): 87 children aged 6-36 mo. with persistent diarrhea were assigned to a rice-lentil (Khitchri) and yogurt diet + 3mg/kg/d elemental zinc or placebo for 14 d until discharge from hospital. Primary outcome: overall weight gain by day 14. Secondary outcomes: overall energy intake, stool output, time to diarrheal recovery and weight gain (>3 days), plasma zinc, copper, prealbumin, and insulin-like growth factor-1 | 3 mg/kg/d (~18 mg/d) | 14 days | Supplement | · Plasma Cu | · Yes |
|  |  |  |  |  | · Lactulose:rhamnose ratio | · No |
|  |  |  |  |  | · Stool volume, frequency | · No |
| Botash et al. (11)  (USA)  Synthesis: N | Case report of 13 mo. infant who had been prescribed prophylactic zinc gluconate since 6 months of age due to a 4-year-old sibling having acrodermatitis enteropathica associated with low serum zinc | 16-24 mg/d  (Case report, n=1) | 6 months | Supplement (prophylaxis; sibling had acrodermatitis enteropathica) | · Hemoglobin | · Yes |
|  |  |  |  |  | · Reticulocytes | · Yes |
|  |  |  |  |  | · Serum iron | · No |
|  |  |  |  |  | · Total iron-binding capacity | · No |
|  |  |  |  |  | · Mean cell volume | · Yes |
|  |  |  |  |  | · White cell count | · Yes |
|  |  |  |  |  | · Neutrophils, lymphocytes, monocytes | · Yes |
|  |  |  |  |  | · Serum Cu | · Yes |
|  |  |  |  |  | · Ceruloplasmin | · Yes |
|  |  |  |  |  | · Skeletal | · Yes |
|  |  |  |  |  | · Bone marrow | · Yes |
|  |  |  |  |  | · Hair | · Yes |
|  |  |  |  |  | · Weight gain | · Yes |
| Brooks et al. (12)  (Bangladesh)  Synthesis: N & M | RCT (double blinded, placebo controlled): 1665 poor, urban children aged 60 days to 12 months were randomly assigned zinc (70 mg) or placebo orally once weekly for 12 months to examine effect on incidence of pneumonia & diarrhea | 21 mg (once weekly) | 12 months | Supplement | · Taste aversion/regurgitation | · Yes |
|  |  |  |  |  | · Hemoglobin | · No |
|  |  |  |  |  | · White blood cell count, differential | · No |
|  |  |  |  |  | · Serum Cu | · No |
|  |  |  |  |  | · Diarrhea | · No |
|  |  |  |  |  | · Upper respiratory tract infection | · No |
|  |  |  |  |  | · Pneumonia, severe pneumonia | · No |
|  |  |  |  |  | · Suppurative otitis media | · No |
|  |  |  |  |  | · Reactive airways disease or bronchiolitis, | · No |
|  |  |  |  |  | · Death | · No |
| Brown et al. (13)  (Peru) Synthesis: N & M | RCT (double blind): 302 6-8 mo. old children with LAZ <-0.5 (and presumed at high risk of Zn deficiency) were assigned to 1) 30 g dry weight of an iron-fortified cereal porridge and a separate dose of an aqueous multivitamin (MV) supplement between meals (control group), 2) the same porridge and MV with 3 mg Zn added to the supplement dose (Zn Supplementation group), or 3) the porridge with added zinc (150 mg/kg dry weight) and MV without zinc (Zn Fortification group) for 6 mo. | 3 mg/d | 6 months | Supplement + fortified porridge | · Hemoglobin | · No |
|  |  |  |  |  | · Anemia | · No |
|  |  |  |  |  | · Serum Ferritin | · No |
|  |  |  |  |  | · Iron deficiency | · No |
|  |  |  |  |  | · Iron deficiency anemia | · No |
|  |  |  |  |  | · Diarrhea | · No |
|  |  |  |  |  | · Upper respiratory tract infection | · No |
|  |  |  |  |  | · Lower respiratory tract infection | · No |
|  |  |  |  |  | · Fever | · No |
|  |  |  |  |  | · Diminished appetite | · No |
| Carter et al. (14)  (Tanzania)  Synthesis: N & M | RCT (secondary analysis of a double blind, placebo controlled 2x2 factorial design trial): ﻿6 wk old infants were randomized for 18 mo. to daily Zn, multivitamins (MV), Zn and MV, or placebo treatment arms. ﻿Hb and red blood cell indices were measured at baseline and at 6, 12, and 18 mo. of age. Plasma samples from 589 infants were examined for iron deficiency at 6 mo. | 6 wk-6 mo.: 5 mg/d ≥7 mo.: 10 mg/d | 18 months | Supplement | · Hemoglobin | · No |
|  |  |  |  |  | · Serum Ferritin | · No |
|  |  |  |  |  | · Iron deficiency | · Yes |
|  |  |  |  |  | · Soluble transferrin receptor | · No |
|  |  |  |  |  | · Anemia | · No |
| Caulfield et al. (15)  (Peru)  Synthesis: N & M | RCT (double blind, placebo controlled): 251 infants aged 6 mo. were assigned to daily supplements of 10 mg of iron and 0.5 mg of copper with or without 10 mg of zinc from 6 to 18 mo. of age. | 10 mg /d | 12 months | Supplement | · Hemoglobin | · No |
|  |  |  |  |  | · Serum Ferritin | · No |
|  |  |  |  |  | · Plasma Cu | · No |
| Chang et al. (16) (Bangladesh)  Synthesis: N & M | RCT (double blind, placebo controlled): 1000 infants aged 6-18 mo. were randomized for 6 mo. to daily placebo; alternate days placebo and Zn (average 2.5 mg/d 6-11 mo. [i.e., 5 mg on alternate days]; average 5 mg/d 12-18 mo. [10 mg on alternate days); alternate days placebo and iron/folic acid + Zn; alternate days placebo and iron/folic acid; or alternate days iron and Zn | 6-11 mo.: 5 mg (alternate days)  ≥ 12 mo.: 10 mg (alternate days) | 6 months | Supplement | · Diarrhea, dysentery | · No |
|  |  |  |  |  | · Hemoglobin | · No |
|  |  |  |  |  | · Serum transferrin receptor | · No |
|  |  |  |  |  | · Anemia | · No |
|  |  |  |  |  | · CRP | · No |
|  |  |  |  |  | · Vomiting | · No |
|  |  |  |  |  | · Infectious hospitalizations, bronchiolitis hospitalizations | · No |
| Chen et al. (17)  (China)  Synthesis: N & M | RCT (cluster randomized by class): 290 preschoolers aged 36-72 mo. were randomized to 1 of 3 treatment groups: 1) vitamin A, 2) vitamin A plus zinc (10 mg/d 5x/wk), and 3) vitamin A combined with additional multiple micronutrients (0 mg Zn) for 6 mo. | 10 mg (5 d/wk)  + dietary intake 7 mg/d | 6 months | Supplement | · Anemia | · No |
|  |  |  |  |  | · Hemoglobin | · No |
| Chhagan et al. (18)  (South Africa) Synthesis: M | RCT (double-blind, placebo controlled): 373 children aged 6 mo. were randomized separately in 3 cohorts - (32 HIV-infected children, 154 HIV-uninfected children born to HIV- infected mothers, and 187 uninfected children born to HIV-uninfected mothers) to receive daily vitamin A (VA) [n = 124], vitamin A plus zinc (VAZ) [n = 123], or multiple micronutrients that included vitamin A and zinc (MM) [n = 126] for 18 months | 10 mg /d | 18 months | Supplement, crushed into porridge | · Anemia | · No |
|  |  |  |  |  | · Hemoglobin | · Yes |
| Dijkhuizen et al. (19)  (Indonesia)  Synthesis: N | RCT (double blind, placebo controlled): 478 infants aged 4 mo. were supplemented for 6 mo. with iron (10 mg/d), zinc (10 mg/d), iron + zinc (10 mg of each/d) or placebo | 10 mg /d | 6 months | Supplement | · Hemoglobin | · No |
|  |  |  |  |  | · Serum Ferritin | · No |
|  |  |  |  |  | · Anemia | · No |
|  |  |  |  |  | · Iron deficiency anemia | · No |
| Fahmida et al. (20)  (Indonesia)  Synthesis: N & M | RCT (double blind, placebo controlled): 800 infants aged 3-6months in rural Indonesia were assigned to daily Zn (10 mg/d), zinc+iron, Zn+Fe (10 mg/d of each), zinc+iron+vitamin-A, Zn+Fe+vitA (10 mg/d of each zinc and iron plus 1,000 IU vitamin A), or placebo for 6 mo. | 10 mg /d | 6 months | Supplement | · Hemoglobin | · No |
|  |  |  |  |  | · Serum Ferritin | · No |
| Fallah et al. (21)  (Iran)  Synthesis: N | RCT (single blind, placebo controlled): 18 to 60 mo. old children presenting with first febrile seizure and having a normal serum Zn randomized to 2 mg/kg/d Zn or placebo for 6 mo. | 0.45 mg/kg/d (~6 mg/d) | 6 months | Supplement | · Vomiting | · No |
|  |  |  |  |  | · Heartburn | · No |
|  |  |  |  |  | · Abdominal pain | · No |
|  |  |  |  |  | · All gastrointestinal side effects | · No |
| Gupta et al. (22)  (India)  Synthesis: N | RCT (double blind, placebo controlled): ﻿280 rural children aged 6 to 41 mo. were assigned to 10 mg zinc/d for 5 d/ wk, 50 mg zinc once weekly (other 4 days received placebo) or placebo for 16 wk | 10 mg (5 d/wk.)  50 mg/d (weekly) | 16 weeks | Supplement | · General adverse effects | · No |
|  |  |  |  |  | · Diarrhea | · No |
| Hambidge et al. (23)  (USA) Synthesis: M | Quasi-experimental study: 96 children aged 33-90 mo. assigned to zinc fortified cereal or non-fortified cereal for 9 mo. Biochemical parameters including serum copper collected | 3.75 mg (6 d/wk) | 9 months | Fortified cereal | · Plasma copper | · No |
|  |  |  |  |  | · Serum cholesterol | · No |
| Hess et al. (24)  (Burkina Faso) Synthesis: N & M^[[2]](#footnote-3)^ | RCT (cluster randomized, partially double blinded): 2435 children aged 9 mo. of age were assigned to the intervention cohort (1) small quantity lipid based nutrient supplement [SQ-LNS] without added Zn + placebo tablet; 2) SQ-LNS with 5 mg added Zn + placebo tablet; 3) SQ-LNS with 10 mg added Zn + placebo tablet; 4) SQ-LNS without added Zn + 5 mg Zn tablet) until 18 mo. of age and 785 children were assigned to the non-intervention group who did not receive SQ-LNS or tablets from 9 to 18 mo. of age | 5 mg/d  10 mg/d |  |  | · Hemoglobin | · No |
|  |  |  |  |  | · Anemia | · No |
|  |  |  |  |  | · CRP | · No |
|  |  |  |  |  | · α-1-acid glycoprotein | · No |
|  |  |  |  |  | · Diarrhea | · No |
|  |  |  |  |  | · Malaria | · No |
|  |  |  |  |  | · Hospitalization | · No |
|  |  |  |  |  | · Death | · No |
| Kurugöl et al. (25)  (Turkey)  Synthesis: N | RCT (double blind, placebo controlled): ﻿ 200 healthy children 2-10y of age receive either 15 mg zinc sulphate or placebo for 7 mo.; the Zn dose was increased to two times per day (30 mg of zinc) at the onset of cold, until symptoms resolved | 15 mg/d | 7 months | Supplement | · Nausea/vomiting | · No |
|  |  |  |  |  | · Abdominal pain | · No |
|  |  |  |  |  | · Taste aversion | · No |
|  |  |  |  |  | · Diarrhea, constipation | · No |
|  |  |  |  |  | · Mouth irritation | · No |
|  |  |  |  |  | · Sleepiness, headache, itching, dizziness | · No |
|  |  |  |  |  | · All adverse effects | · No |
| Kurugöl et al. (26)  (Turkey)  Synthesis: N | RCT (double blind, placebo controlled): 150 children presenting with at least two of 10 symptoms of common cold within the 24 – 48 h of the onset of illness were randomized to Zn (30 mg/d) or placebo for 10 days | 30 mg/d | 10 days | Supplement | · Nausea/vomiting | · No |
|  |  |  |  |  | · Abdominal pain | · No |
|  |  |  |  |  | · Taste aversion | · No |
|  |  |  |  |  | · Diarrhea, constipation | · No |
|  |  |  |  |  | · Mouth irritation | · No |
|  |  |  |  |  | · Drowsiness | · No |
|  |  |  |  |  | · Dry mouth | · No |
|  |  |  |  |  | · Any adverse effect | · No |
| Larson et al. (27)  (India)  Synthesis: N | RCT (double blind, placebo controlled): 1600 children aged 3-59 mo. with acute diarrhea were randomized to one of three groups: no treatment, placebo, or Zn sulphate tablet (20 mg). They were then observed for 60 minutes, and all vomiting or regurgitation episodes were recorded. after the 60 min observation period, all children who had not already had Zn were given it | 20 mg (once only) | 1 day | Supplement | · Vomiting | · Yes |
|  |  |  |  |  | · Regurgitation | · Yes |
| Lind et al. (28)(Indonesia)  Synthesis: N & M | RCT (double blind, placebo controlled): infants (n=680) were randomly assigned to daily supplementation with 10 mg Fe (Fe group), 10 mg Zn (Zn group), 10 mg Fe + 10 mg Zn (Fe+Zn group), or placebo from 6 to 12 mo. of age. | 10 mg/d | 6 months | Supplement | · Vomiting | · No |
|  |  |  |  |  | · Hemoglobin | · Yes |
|  |  |  |  |  | · Serum Ferritin | · Yes |
|  |  |  |  |  | · Serum transferrin receptor | · No |
|  |  |  |  |  | · Serum Cu | · No |
|  |  |  |  |  | · Anemia | · Yes |
|  |  |  |  |  | · Iron deficiency | · Yes |
|  |  |  |  |  | · Iron deficiency anemia | · No |
| López de Romaña et al. (29)  (Peru)  Synthesis: M | RCT: 41 children aged 3-4 y and at high risk of Zn deficiency assigned to 2 meals/d a total of 100 g wheat products fortified with 3 mg Fe as ferrous sulphate and either 0 (group Zn-0), 3 (group Zn-3) or 9 (group Zn-9) mg Zn as zinc sulphate per 100 g flour for 70 d. | 3 mg/d    9 mg/d | 70 days | Fortified wheat products | · Hemoglobin | · No |
|  |  |  |  |  | · Anemia | · No |
|  |  |  |  |  | · Serum Ferritin | · No |
| Malik et al. (30)  (India)  Synthesis: N | RCT (double blind, placebo controlled): 272 children aged 6-11 mo. with acute respiratory infections randomized to zinc 20 mg/d or placebo for 2 weeks, and followed up over several months for incidence of ARI | 20 mg/d | 2 weeks | Supplement | · Vomiting | · No |
|  |  |  |  |  | · Diarrhea | · No |
|  |  |  |  |  | · Constipation | · No |
|  |  |  |  |  | · Acute respiratory infection | · No |
|  |  |  |  |  | · Death | · No |
| Martinez-Estevez et al. (31)  (Colombia)  Synthesis: N | RCT (triple blind, placebo controlled): 355 healthy children aged 6 to 12 mo. randomized to Zn 5mg/d or placebo for 12 mo. The outcomes analyzed were incidence of URTI, acute diarrheal disease episodes, and side effects of the interventions. | 4 mg/d | 12 months | Supplement | · General adverse effects | · No |
|  |  |  |  |  | · Vomiting | · No |
|  |  |  |  |  | · Constipation | · No |
|  |  |  |  |  | · Upper respiratory tract infection | · No |
|  |  |  |  |  | · Diarrhea | · No |
| Mendoza et al. (32)  (Venezuela) Synthesis: M | Quasi-experimental (double blind controlled trial): 58 children aged 2-6 y with WAZ + HAZ <10th centile assigned to zinc fortified (4.12 mg/d) or non-fortified biscuits for 16 weeks. | 4.12 mg/d | 16 weeks | Fortified biscuits | · Hemoglobin | · No |
|  |  |  |  |  | · Hematocrit | · No |
|  |  |  |  |  | · Serum Ferritin | · No |
|  |  |  |  |  | · Anemia | · No |
| Moradveisi et al. (33)  (Iran) Synthesis: M | RCT: 88 children aged 6 mo. - 4y with mild-moderate IDA were assigned to receive zinc + iron (1 mg Zn/kg/d) or iron alone (5 mg Fe/kg/d) for 1 month. | 12.3 mg/d | 1 month | Supplement | · Hemoglobin | · No |
|  |  |  |  |  | · Hematocrit | · No |
|  |  |  |  |  | · Serum Ferritin | · No |
|  |  |  |  |  | · Serum iron | · No |
|  |  |  |  |  | · Total iron binding capacity | · No |
|  |  |  |  |  | · Mean cell volume | · No |
|  |  |  |  |  | · Pallor | · No |
|  |  |  |  |  | · Irritability | · No |
|  |  |  |  |  | · Pica | · No |
|  |  |  |  |  | · Low appetite | · No |
| Muñoz et al. (34)  (Mexico) ^[[3]](#footnote-4)^ Synthesis: -- | RCT (double blind, placebo controlled): 219 rural Mexican children aged 18–36 mo. were assigned to 20 mg Zn/d, 20 mg Fe/d, 20 mg Zn/d + 20 mg Fe/d, or placebo for 6 mo. | 20 mg/d | 6 months | Supplement (provided in a beverage) | · Hemoglobin | · No |
|  |  |  |  |  | · Serum Ferritin | · No |
| Olney, 2 et al. (35)  (Tanzania (Zanzibar))  Synthesis: N & M^[[4]](#footnote-5)^ | RCT (double blind, placebo controlled): 212 children aged 5-11 mo. at enrolment randomized to 1) iron + folic acid (FeFA) (12.5 mg Fe + 50 ug folic acid), 2) Zn 10 mg, 3) iron + folic acid + Zn 10 mg (FeFA + Zn) or 4) placebo for 12 mo. Children < 12 mo. received half the dose | <12 mo.: 5 mg/d  ≥ 12 mo.: 10 mg/d | 6 months | Supplement | · Hemoglobin | · No |
|  |  |  |  |  | · Iron deficiency | · No |
|  |  |  |  |  | · Anemia | · No |
|  |  |  |  |  | · Zinc protoporphyrin | · No |
| Ouédraogo et al. (36)  (Burkina Faso) Synthesis: M | RCT (double blind): 299 children aged 6-23 mo. with mild-moderate anemia were assigned to iron (15 mg Fe x 5 d/wk), iron + zinc (10.2 mg Zn x 5 d/wk) or multiple micronutrients for 6 months. | 10.2 mg  (5 d/wk) | 6 months | Supplement | · Hemoglobin | · No |
|  |  |  |  |  | · Anemia | · No |
| Owusu-Agyei et al. (37)  (Ghana)  Synthesis: M | RCT (double blind, placebo controlled): 200 children aged 6-24 mo. assigned to Zinc 10 mg/d + Vit A or Vit A alone for 6 mo. | 10 mg/d | 6 months | Supplement | · Hemoglobin | · No |
|  |  |  |  |  | · Anemia | · No |
|  |  |  |  |  | · Malaria | · No |
|  |  |  |  |  | · Diarrhea | · No |
|  |  |  |  |  | · Lower respiratory tract infection | · No |
| Passariello et al. (38)  (Italy)  Synthesis: N | RCT (single blind, controlled): 83 children aged 5-36 mo. presenting with acute gastroenteritis (diarrhea) to family pediatricians were randomized tr receive standard hypotonic ORS or a gel hypotonic super-ORS containing zinc. The main study outcome was ORS intake in the first 24 h. ORS intake at 4 h, rate of diarrhea resolution at 72 h of treatment, total duration and severity of diarrhea, hospitalization, and adverse effects were also evaluated. | ~10-20 mg/24 hours | 24 hours | Gel oral rehydration solution | · General adverse effects | · No |
|  |  |  |  |  | · Diarrhea | · No |
|  |  |  |  |  | · ORS consumption | · No |
| Radhakrishna et al. (39)  (India)  Synthesis: N & M | RCT (double blind, placebo controlled): 324 infants aged 4 mo. supplemented with Zn 5 mg/d + 0.5 mg riboflavin or placebo + riboflavin for 14 mo. | 5 mg/d  + dietary intake 0.97 mg/d | 12 months | Supplement | · Hemoglobin | · No |
|  |  |  |  |  | · Cu | · No |
|  |  |  |  |  | · Diarrhea | · No |
|  |  |  |  |  | · Respiratory infection | · No |
| Rahman et al. (40)  (India)  Synthesis: N | RCT (double blind, placebo controlled): ﻿2x2 factorial trial where 800 children aged 12-35 mo. living in urban slums in Dhaka were assigned to either: 20 mg zinc once daily for 14 days; 200 000 IU vitamin A, single dose on day 14; both zinc and vitamin A; or placebo. The children were followed up once a week for six months, and morbidity information was collected. | 20 mg/d | 14 days | Supplement | · Acute lower respiratory tract infection | · Yes |
|  |  |  |  |  | · Severe acute lower respiratory tract infection | · Yes |
| Richard et al. (41)  (Peru)  Synthesis: N & M | RCT (double blind, placebo controlled): 855 children aged 6 mo. to 15 y assigned to 1) iron 15 mg 2) zinc 20 mg, 3) iron + zinc or 4) placebo for 7 mo. | 20 mg/d | 7 months | Supplement | · Hemoglobin | · No |
|  |  |  |  |  | · Malaria | · No |
|  |  |  |  |  | · Diarrhea | · No |
|  |  |  |  |  | · Acute lower respiratory tract infection | · No |
|  |  |  |  |  | · Death | · No |
| Rosado et al. (42)  (Mexico)^[[5]](#footnote-6)^ Synthesis: N & M | RCT (double blind, placebo controlled): 219 children aged 18-36 mo. were supplemented with 20 mg Zn as zinc methionine, 20 mg Fe as ferrous sulphate, 20 mg Zn + 20 mg Fe, or a placebo for 12 months. | 20 mg  (6 d/wk) | 12 months | Supplement (beverage) | · Hemoglobin | · No |
|  |  |  |  |  | · Serum Ferritin | · No |
|  |  |  |  |  | · Iron deficiency | · No |
|  |  |  |  |  | · Respiratory disease | · No |
|  |  |  |  |  | · Diarrhea | · No |
|  |  |  |  |  | · Fever | · No |
| Ryan et al. (43)  (Malawi) Synthesis: N | RCT (double-blind, placebo controlled): 234 children aged 1-3 y assigned to zinc 20 mg, albendazole (1 dose) or placebo for 14 d. | 20 mg/d | 14 days | Supplement | · Lactulose:mannitol | · No |
| Sampaio et al. (44)  (Brazil)  Synthesis: N | RCT (double blind, placebo controlled): 143 healthy children, aged 6 to 48 mo., were assigned to sprinkles (multiple micronutrients) or sprinkles + zinc for 90 days and followed regarding the outcomes of diarrhea and ARI. | 5 mg/d | 3 months | Sprinkles | · Acute respiratory infection | · No |
|  |  |  |  |  | · Diarrhea | · No |
| Sazawal et al. (45)  (India)  Synthesis: N & M | RCT (double blind, placebo controlled): subsample of 115 children (Zn 61, Cu 54) aged 5-12 mo. were randomly selected for inclusion in this sub-study [in the original trial, 609 children aged 6 to 35 mo. and presenting with acute diarrhea were enrolled and assigned to zinc 10 mg or control groups] | 10 mg/d | 4 months | Supplement | · Hemoglobin | · No |
|  |  |  |  |  | · Hematocrit | · No |
|  |  |  |  |  | · Mean corpuscular volume, Hb | · No |
|  |  |  |  |  | · Platelets | · No |
|  |  |  |  |  | · Granulocytes, monocytes, eosinophils | · No |
|  |  |  |  |  | · Plasma Cu | · No |
| Sazawal et al. (46)  (Tanzania [Pemba])^[[6]](#footnote-7)^ Synthesis: N & M NB. | RCT (cluster randomized, placebo-controlled, double blind): 24,076 children aged 1-35 mo. were assigned to iron + folic acid, FeFA + Zn (10 or 5 mg by age), or placebo (zinc only arm not included in this report) for ~12 mo. (FeFA/FeFA+ Zn stopped early due to safety concerns). Primary endpoints were all-cause mortality and admission to hospital. | <12 mo.: 5 mg/d    ≥12 mo.: 10 mg/d | 12 months | Supplement | · General adverse effects | · No |
|  |  |  |  |  | · Death | · No |
|  |  |  |  |  | · Hospitalizations | · No |
|  |  |  |  |  | · Malaria | · No |
|  |  |  |  |  | · Infections | · No |
|  |  |  |  |  | · Diarrhea | · No |
|  |  |  |  |  | · Severe anemia | · No |
| Shankar et al. (47)  (Papua New Guinea)  Synthesis: N & M | RCT (double-blind, placebo-controlled): 274 children aged 6 to 60 months were assigned to 10 mg Zn or placebo 6 d/wk for 46 wks. | 10 mg  (6 d/wk) | 46 weeks | Supplement | · Hemoglobin | · No |
|  |  |  |  |  | · Anemia | · No |
|  |  |  |  |  | · Malaria | · Yes |
|  |  |  |  |  | · Hospital visits/admissions | · No |
| Silva et al. (48)  (Brazil)  Synthesis: N & M | RCT (Single blinded, placebo controlled): 58 children aged 12 to 59 mo. enrolled in a nutritional recovery program received 10 mg/day of zinc sulphate or placebo for four months | 10 mg/d | 4 months | Supplement | · Hemoglobin | · No |
|  |  |  |  |  | · Hematocrit | · No |
|  |  |  |  |  | · Serum iron | · No |
| Smith et al. (49)  (Belize)  Synthesis: N | RCT (double blind, placebo controlled): 43 children aged 22 to 66 mo. either Zn, vitamin A, Zn and vitamin A or a placebo, (70 mg Zn and/or 3030 RE vitamin A, once per week) for 6 mo. in a 2x2 factorial design. | 70 mg (once weekly) | 6 months | Beverage | · Hemoglobin | · No |
| Strand et al. (50)  (Nepal)  Synthesis: N & M | RCT (double blind, placebo controlled): 1792 cases of acute diarrhea in children aged 6-35 mo. randomized to 1) placebo, 2) Zinc, 3) Zinc + massive dose vit A, 4) (open) zinc daily | <12 mo.: 15 mg/d ≥ 12 mo.: 30 mg/d | 10 days | Supplement | · Plasma Cu | · No |
|  |  |  |  |  | · Regurgitation | · Yes |
|  |  |  |  |  | · Vomiting | · Yes |
|  |  |  |  |  | · Diarrhea | · No |
| Sugiura et al. (51)  (Japan)  Synthesis: N | Case report: 2 y 4 mo. boy was treated with 314 mg/Zinc gluconate/d (45 mg elemental Zn/d) for atopic dermatitis at another dermatology clinic since the age of 1 y and 5 mo. | 45 mg (daily)  (Case report, n=1) | 11 months | Supplement (treatment for atopic dermatitis) | · Skin | · Yes |
|  |  |  |  |  | · Hair | · Yes |
|  |  |  |  |  | · Anthropometry | · No |
|  |  |  |  |  | · Hepatosplenomegaly | · Yes |
|  |  |  |  |  | · Development | · Yes |
|  |  |  |  |  | · Demyelination (on MRI) | · No |
|  |  |  |  |  | · White cell count | · Yes |
|  |  |  |  |  | · Hemoglobin | · Yes |
|  |  |  |  |  | · Mean corpuscular volume | · Yes |
|  |  |  |  |  | · Platelets | · Yes |
|  |  |  |  |  | · Immunoglobulins | · Yes |
|  |  |  |  |  | · Serum Cu | · Yes |
|  |  |  |  |  | · Ceruloplasmin | · Yes |
| Surono et al. (52)  (Indonesia)  Synthesis: N | RCT (double blind, placebo controlled): 2x2 factorial trial, 48 children aged 12-24 mo. were assigned to powder containing the probiotic Lactobacillus plantarum IS-10506, zinc [20 mg zinc sulphate monohydrate (8 mg zinc elemental)], L. plantarum IS-10506 + zinc, or placebo for 90 days. Blood and stool samples were collected at baseline and at the end of the study period. | 8 mg/d | 3 months | Supplement | · General adverse events | · No |
|  |  |  |  |  | · Fecal secretory IgA | · No |
| Valentiner-Branth, et al. (53) (Nepal)  Synthesis: N | RCT (double blind, placebo controlled): children aged 2–35 mo. with severe (n = 149) or non-severe (n = 2479) pneumonia were assigned to receive zinc (10 mg if aged 2–11 mo., or 20 mg >=12 mo.) or placebo daily for 14 d as an adjuvant to antibiotics. The primary outcomes were treatment failure, defined as a need for change in antibiotics or hospitalization, and time to recovery from pneumonia. | 2-11 mo.: 10 mg/d  ≥ 12 mo.: 20 mg/d | 14 days | Supplement | · Vomiting/regurgitation within 15 mins of initial dose | · Yes |
|  |  |  |  |  | · Vomiting initial 24 hours | · Yes |
|  |  |  |  |  | · Vomiting/regurgitation during 14 days of supplementation | · Yes |
|  |  |  |  |  | · Pneumonia | · No |
| Veenemans et al. (54)  (Tanzania)  Synthesis: N & M | RCT (double blind, placebo controlled): 2x2 factorial trial, 612 rural Tanzanian children aged 6–60 months in an area with intense malaria transmission and with HAZ <-1.5 were randomized to receive daily oral supplementation with either zinc alone (10 mg), multi-nutrients without zinc, multi-nutrients with zinc, or placebo for a median of 251 days (95% reference range: 191–296 days) | 10 mg/d | 8.4 months | Supplement | · Malaria | · No |
|  |  |  |  |  | · Inflammation (CRP) | · No |
|  |  |  |  |  | · Anemia | · No |
|  |  |  |  |  | · Plasma ferritin | · No |
|  |  |  |  |  | · Iron deficiency | · No |
|  |  |  |  |  | · Hemoglobin | · No |
| Walravens et al. (55)  (USA)  Synthesis: N & M | Quasi-experimental: 68 full-term healthy neonates assigned to control (Similac with iron, Zn 1.8 mg/L) or zinc fortified (Similac with iron, Zn 5.8 mg/L) from age 4-6 d to 6 mo. | 3.8 mg/d | 6 months | Fortified formula | · Hematocrit | · No |
|  |  |  |  |  | · Plasma copper | · No |
|  |  |  |  |  | · Plasma cholesterol | · No |
|  |  |  |  |  | · Vomiting | · No |
|  |  |  |  |  | · Regurgitation | · No |
|  |  |  |  |  | · Diarrhea | · No |
|  |  |  |  |  | · GI disturbance | · No |
| Wasantwisut et al. (56)  (Thailand)  Synthesis: N & M | RCT (double blind, placebo controlled): ﻿2x2 factorial design, 609 rural predominantly breastfed Thai infants aged 4–6 mo. were supplemented daily with 10 mg of iron and/or 10 mg of zinc for 6 mo. to investigate effects and interactions on micronutrient status and growth | 10 mg/d | 6 months | Supplement | · Hemoglobin | · Yes |
|  |  |  |  |  | · Serum Ferritin | · Yes |
|  |  |  |  |  | · Anemia | · Yes |
|  |  |  |  |  | · Iron deficiency | · Yes |
|  |  |  |  |  | · Iron deficiency anemia | · Yes |
|  |  |  |  |  | · Iron deficiency anemia | · No |
| Wuehler et al. (57)  (Ecuador)  Synthesis: N & M | RCT (double blind, placebo controlled): 631 Ecuadorian children aged 12–30 mo. with LAZ< -1.3 were assigned to 1 of 5 daily supplements for 6 mo.: 3, 7, or 10 mg Zn as zinc sulphate, 10 mg Zn + 0.5 mg Cu as copper sulphate, or placebo. | 3 mg/d  7 mg/d  10 mg/d | 6 months | Supplement | · Hemoglobin | · No |
|  |  |  |  |  | · Serum Ferritin | · No |
|  |  |  |  |  | · Plasma Cu | · No |
|  |  |  |  |  | · Ceruloplasmin | · No |
|  |  |  |  |  | · Erythrocyte super oxide dismutase activity | · No |
|  |  |  |  |  | · HDL, total cholesterol | · No |
|  |  |  |  |  | · Diarrhea | · No |
|  |  |  |  |  | · Low appetite | · No |
|  |  |  |  |  | · Cough | · No |
|  |  |  |  |  | · Nasal discharge | · No |
|  |  |  |  |  | · Fever | · No |
| Zlotkin et al. (58)  (Ghana)  Synthesis: M | RCT (double-blind, controlled): 304 anemic infants aged 6-18 mo. were assigned to FeZn + vit C (80 mg Fe, 10 mg Zn, 50 mg ascorbic acid) or Fe + Vit C for 2 mo. | 10 mg/d | 2 months | Sprinkles | · Hemoglobin | · Yes |
|  |  |  |  |  | · Anemia | · Yes |
|  |  |  |  |  | · Serum Ferritin | · No |
|  |  |  |  |  | · Iron deficiency | · No |
|  |  |  |  |  | · Malaria | · No |

**Note:** Reference numbers for this table are per the reference list at the end of this supplementary file. Synthesis N= narrative analysis, M- meta-analysis. ARI: acute respiratory infection, CRP: C-reactive protein, GI: gastrointestinal infection, HAZ: height-for-age z-score, IDA: iron deficiency anemia, IFA: iron folic acid, LAZ: length-for-age z-score, MRI: magnetic resonance imaging, MV: multivitamin, NRCT: non-randomized controlled trial, ORS: oral rehydration solution, RE: retino equivalents, RCT: randomized controlled trial, SQ-LNS: small quantity lipid based nutrient supplement, SF serum ferritin: SZn: serum zinc, URTI: upper respiratory tract infection: VA: vitamin A: VAZ: vitamin A plus Zinc, WAZ: weight-for-age z-score.

# **Table 2** Dietary intake of arms included in the meta-analyses

| **Author (Year)** | **Country** | **Dietary Zn intake (mg/d)** | **Total dietary Zn intake (incl. breast milk intake)**^[[7]](#footnote-8)^ **(mg/d)** | **Mean Zn dose study arm 1 (mg/d)** | **Mean Zn dose study arm 2**  **(mg/d)** | **Mean Zn dose study arm 3**  **(mg/d)** | **Age range**  **[mean] in study** | **Age range [mean] in external study** | **Mean Plasma [Zn] at baseline (µg/dL) [% deficient]** | **Data Source** |
| --- | --- | --- | --- | --- | --- | --- | --- | --- | --- | --- |
| Abbeddou et al. (1) | Burkina Faso | 5.4 | 5.4 | 5 | 10 | 5 | 8.8-9.9 [9.4] mo. | 24-36 mo. | 66.2 [35] | Provided by authors of other study: (59) |
| Abdollahi et al. (2) | Iran | 3.44 | 3.44 | 5 | . | . | 6-24 [15.9] mo. | . | 83.5 [20.6] | Provided by authors |
| Alarcon et al. (3) | Peru | 3.8 | 4.09 | 6.3 | . | . | 6-35 [17.5] mo. | 6-35 mo. | NR | (60) |
| Baqui et al. (5) | Bangladesh | 1.2 | 1.2 | 2.9 | 2.9 | . | 6 [6.4] mo. | 6-8 [7.1] mo. | 67.2 [45.6] | (61) |
| Bates et al. (6) | Gambia | 4.31 | 4.31 | 20 | . | . | 6-26 [17.6] mo. | 17 mo. | 106.6 [NR] | (62) |
| Berger et al. (7) | Vietnam | 1.1 | 2.28 | 10 | 10 | . | 4-6 [5.9] mo. | 6-9 mo. | 93.7 [1.0] | (63) |
| Bhandari et al. (8) | India | 1.3 | 1.59 | <12 mo.: 10  ≥12 mo.: 20 | . | . | 6-30 [15.3] mo. | 10-18 mo. [14.8] mo. | 62.0 [44.0] | (64) |
| Bhandari et al. (9) | India | 2.7 | 3.34 | <6 mo.: 5  ≥6 mo.: 10 | . | . | 1-23 [11.7] mo. | 9-24 mo. | 64.1 [35.8] | (65) |
| Bhutta et al. (10) | Pakistan | 3.0 | 3.0 | 18 | . | . | 6-36 [12.4] mo. | 0-23 mo. | 74.2 [29.0] | (66) |
| Brooks et al. (12) | Bangladesh | 0.78 | 1.99 | 3 | . | . | 2-11 [5.3] mo. | 9-12 mo. | 64.1 [NR] | (67) |
| Brown et al. (13) | Peru | 1.5 | 1.5 | 3 | . | . | 6-8 [7.5] mo. | 6-8 [7.5] mo. | 77.6 [16.8] | (68) |
| Carter et al. (14) | Tanzania | . | 2.03 | <7 mo.: 5  ≥7 mo.: 10 | <7 mo.: 5  ≥7 mo.: 10 | . | 6 wks. | . | NR | Exclusive breastfeeding data used |
| Caulfield et al. (15) | Peru | 2.2 | 2.84 | 10 | . | . | 6 [6.3] mo. | 6-11 mo. | 72.2 [NR] | (60) |
| Chang et al. (16) | Bangladesh | 1.3 | 1.94 | <12 mo.: 2.5  ≥12 mo.: 5 | <12 mo.: 2.5  ≥12 mo.: 5 | <12 mo.: 2.5  ≥12 mo.: 5 | 6-18 [11] mo. | 6-59 mo. | 65.0 [NR] | (69) |
| Chen et al. (17) | China | 7.5 | 7.5 | 7.14 | . | . | 36-72 [51.5] mo. | . | 80.0[25.8] | Reported in study (17) |
| Chhagan et al. (18) | South Africa | 0.9 | 1.54 | 10 | . | . | 6 mo. | 6-9 mo. | NR | (70) |
| Dijkhuizen et al. (19) | Indonesia | 1.2 | 2.38 | 10 | 10 | . | 4-6 [4.2] mo. | 6 mo. | 85.0 [27.6] | (71) |
| Fahmida et al. (20) | Indonesia | 1.1 | 1.1 | 10 | . | . | 3-6 [5] mo. | . | 100.0 [NR] | Provided by authors |
| Hambidge et al. (23) | USA | 6.5 | 6.5 | 3.2 | . | . | 33-90 [57.9] | . | 79.9 [NR] | Reported in study (23) |
| Hess et al. (24) | Burkina Faso | 5.4 | 5.4 | 5 | 10 | 5 | 8.8-9.9 [9.4] mo. | 24-36 mo. | 68.3 [NR] | Provided by authors of another study: (59) |
| Lind et al. (28) | Indonesia | 0.84 | 1.48 | 10 | 10 | . | 6 [6.1] mo. | . | 59.0 [78.0] | Provided by authors |
| López de Romaña et al. (29) | Peru | 4.97 | 4.97 | 3 | 9 | . | 3-4 y [46.6 mo.] | . | 75.2 [20.4] | Reported in study (29) |
| Mendoza et al. (32) | Venezuela | 3.24 | 3.24 | 4.12 | . | . | 2-6 y [49 mo.] | . | 83.5 [NR] | Reported in study (32) |
| Moradveisi et al. (33) | Iran | 6 | 6 | 12.3 | . | . | 6-48 [24.2] mo. | 3 y | NR | (72) |
| Olney et al. (35) | Tanzania | 1.48 | 2.12 | <12 mo.: 5  ≥12 mo.: 10 | <12 mo.: 5  ≥12 mo.: 10 | . | 5-18 [8.8] mo. | 6-11 [8.3] mo. | NR | (73) |
| Ouédraogo et al. (36) | Burkina Faso | 5.4 | 5.4 | 7.29 | . | . | 6-23 [13.1] mo. | 24-36 mo. | NR | Provided by authors of other study: (59) |
| Owusu-Agyei et al. (37) | Ghana | 2.5 | 2.5 | 10 | . | . | 6-24 [14.5] mo. | 6-23 mo. | 75.0 [NR] | (74) |
| Radhakrishna et al. (39) | India | . | 0.94 | 5 | . | . | 4 mo. | . | 67.4 [NR] | Data reported in study, but for 18 mo. of age. Exclusive breastfeeding data used |
| Richard et al. (41) | Peru | 2.7 | 2.7 | 20 | 20 | . | 0.5 to 15 y | 0-14 [7.6] y | 69.0 [46.0] | (75) |
| Rosado et al. (42) | Mexico | 4.5 | 4.5 | 17.14 | . | . | 18-36 [29.8] mo. | 1-4 y | 96.6 [20.0] | (76) |
| Ryan et al. (43) | Malawi | 2.6 | 2.89 | 20 | . | . | 12-36 [22.5] mo. | . | NR | Reported in study (43) |
| Sazawal et al. (45) | India | 1.8 | 2.44 | 10 | . | . | 5-12 [8.5] mo. | 9-12 mo. | 63.9 [NR] | (65) |
| Sazawal et al. (46) | Tanzania | 2.1 | 2.39 | <12 mo.: 5  ≥12 mo.: 10 | . | . | 1-35 [23.1] mo. | 6-23 mo. | NR | (73) |
| Shankar et al. (47) | Papua New Guinea | 4.6 | 4.6 | 8.57 | . | . | 6-60 mo. | 72-96 mo. | 70.5 [26.0] | (77) |
| Silva et al. (48) | Brazil | 3.6 | 3.89 | 10 | . | . | 12-59 [23.5] mo. | 6-24 mo. | 56.3 [100.0] | (78) |
| Strand et al. (50) | Nepal | 2.1 | 2.1 | <12 mo.: 15  ≥12 mo.: 30 | <12 mo.: 15  ≥12 mo.: 30 | . | 6-35 [15.5] mo. | 13-24 mo. | 56.9 [NR] | (79) |
| Veenemans et al. (54) | Tanzania | 3.5 | 3.79 | 10 | 10 | . | 6-60 mo. | 9-24 mo. | 62.8 [67.3] | (65) |
| Walravens et al. (55) | USA | 3.8 | 3.8 | 3.8 | 3.8 | . | 4-6 d | . | 69.5 [NR] | (55) |
| Wasantwisut et al. (56) | Thailand | 2.3 | 2.3 | 10 | 10 | . | 4-6 [4.5] mo. | 9 mo. | 72.4 [50.4]  [33.5] | (80) |
| Wuehler et al. (57) | Ecuador | 4.8 | 4.8 | 3 | 7 | 10 | 9-23 [20.9] mo. | 1-3 y | 70.8 [31.8] | (81) |
| Zlotkin et al. (82) | Ghana | 2.5 | 2.5 | 10 | . | . | 6-18 [10.3] mo. | 6-23 mo. | 131.4 [NR] | (74) |

**Note:** Reference numbers for this table are per the reference list at the end of this supplementary file. Not reported (NR)

# References

1. Abbeddou S, Yakes Jimenez E, Somé JW, Ouédraogo JB, Brown. KH, Hess SY. Small-quantity lipid-based nutrient supplements containing different amounts of zinc along with diarrhea and malaria treatment increase iron and vitamin A status and reduce anemia prevalence, but do not affect zinc status in young Burkinabe children: A cl. BMC Pediatr [Internet]. 2017;17(1):1–17. Available from: http://dx.doi.org/10.1186/s12887-016-0765-9

2. Abdollahi M, Ajami M, Abdollahi Z, Kalantari N, Houshiarrad A, Fozouni F, et al. Zinc supplementation is an effective and feasible strategy to prevent growth retardation in 6 to 24 month children: A pragmatic double blind, randomized trial. Heliyon [Internet]. 2019 Nov;5(11):e02581. Available from: https://doi.org/10.1016/j.heliyon.2019.e02581

3. Alarcon K, Kolsteren PW, Prada AM, Chian AM, Velarde RE, Pecho IL, et al. Effects of separate delivery of zinc or zinc and vitamin A on hemoglobin response, growth, and diarrhea in young Peruvian children receiving iron therapy for anemia. Am J Clin Nutr. 2004;80(5):1276–82.

4. Awasthi S. Zinc Supplementation in Acute Diarrhea is Acceptable, Does Not Interfere with Oral Rehydration, and Reduces the Use of Other Medications. J Pediatr Gastroenterol Nutr [Internet]. 2006 Mar;42(3):300–5. Available from: http://journals.lww.com/jpgn

5. Baqui AH, Fischer Walker CL, Zaman K, El Arifeen S, Chowdhury HR, Wahed MA, et al. Weekly iron supplementation does not block increases in serum zinc due to weekly zinc supplementation in Bangladeshi infants. J Nutr. 2005;135(9):2187–91.

6. Bates CJ, Bates PH, Dardenne M, Prentice A, Lunn PG, Northrop-Clewes CA, et al. A trial of zinc supplementation in young rural Gambian children. Br J Nutr. 1993;69(1):243–55.

7. Berger J, Ninh NX, Khan NC, Nhien N V., Lien DK, Trung NQ, et al. Efficacy of combined iron and zinc supplementation on micronutrient status and growth in Vietnamese infants. Eur J Clin Nutr. 2006;60(4):443–54.

8. Bhandari N, Bahl R, Taneja S, Strand T, Molbak K, Ulvik RJ, et al. Substantial Reduction in Severe Diarrheal Morbidity by Daily Zinc Supplementation in Young North Indian Children. Pediatrics [Internet]. 2002 Jun 1;109(6):e86–e86. Available from: http://www.pediatrics.org/cgi/content/full/109/6/

9. Bhandari N, Taneja S, Mazumder S, Bahl R, Fontaine O, Bhan MK. Adding Zinc to Supplemental Iron and Folic Acid Does Not Affect Mortality and Severe Morbidity in Young Children. J Nutr [Internet]. 2007 Jan 1;137(1):112–7. Available from: https://academic.oup.com/jn/article/137/1/112/4664272

10. Bhutta ZA, Nizami SQ, Isani Z. Diarrhea in Pakistan. Pediatrics. 1999;103(4):1–9.

11. Botash AS. Zinc-Induced Copper Deficiency in an Infant. Arch Pediatr Adolesc Med [Internet]. 1992 Jun 1;146(6):709. Available from: https://ovidsp.ovid.com/ovidweb.cgi?T=JS&CSC=Y&NEWS=N&PAGE=fulltext&D=med3&AN=1595625

12. Brooks WA, Santosham M, Naheed A, Goswami D, Wahed MA, Diener-West M, et al. Effect of weekly zinc supplements on incidence of pneumonia and diarrhoea in children younger than 2 years in an urban, low-income population in Bangladesh: randomised controlled trial. Lancet [Internet]. 2005 Sep;366(9490):999–1004. Available from: https://ovidsp.ovid.com/ovidweb.cgi?T=JS&CSC=Y&NEWS=N&PAGE=fulltext&D=emed9&AN=41327324

13. Brown KH, De Romaña DL, Arsenault JE, Peerson JM, Penny ME. Comparison of the effects of zinc delivered in a fortified food or a liquid supplement on the growth, morbidity, and plasma zinc concentrations of young Peruvian children. Am J Clin Nutr. 2007;85(2):538–47.

14. Carter RC, Kupka R, Manji K, McDonald CM, Aboud S, Erhardt JG, et al. Zinc and multivitamin supplementation have contrasting effects on infant iron status: A randomized, double-blind, placebo-controlled clinical trial. Eur J Clin Nutr [Internet]. 2018 Jan 6;72(1):130–5. Available from: http://www.nature.com/articles/ejcn2017138

15. Caulfield LE, Zavaleta N, Chen P, Colombo J, Kannass K. Mineral status of non-anemic Peruvian infants taking an iron and copper syrup with or without zinc from 6 to 18 months of age: A randomized controlled trial. Nutrition [Internet]. 2013 Nov;29(11–12):1336–41. Available from: https://linkinghub.elsevier.com/retrieve/pii/S0899900713002815

16. Chang S, El Arifeen S, Bari S, Wahed MA, Rahman KM, Rahman MT, et al. Supplementing iron and zinc: Double blind, randomized evaluation of separate or combined delivery. Eur J Clin Nutr [Internet]. 2010 Feb 11;64(2):153–60. Available from: http://www.nature.com/articles/ejcn2009127

17. Chen L, Liu YF, Gong M, Jiang W, Fan Z, Qu P, et al. Effects of vitamin a, vitamin a plus zinc, and multiple micronutrients on anemia in preschool children in Chongqing, China. Asia Pac J Clin Nutr. 2012;21(1):3–11.

18. Chhagan MK, Van Den Broeck J, Luabeya KKA, Mpontshane N, Tomkins A, Bennish ML. Effect on longitudinal growth and anemia of zinc or multiple micronutrients added to vitamin A: A randomized controlled trial in children aged 6-24 months. BMC Public Health. 2010;10:1–11.

19. Dijkhuizen MA, Wieringa FT, West CE, Martuti S, Muhilal. Effects of iron and zinc supplementation in Indonesian infants on micronutrient status and growth. J Nutr. 2001;131(11):2860–5.

20. Fahmida U, Rumawas JSP, Utomo B, Patmonodewo S, Schultink W. Linear Growth of Stunted Infants With Low Haemoglobin. Asia Pac J Clin Nutr. 2007;16(October 2006):301–9.

21. Fallah R, Sabbaghzadegan S, Karbasi SA, Binesh F. Efficacy of zinc sulfate supplement on febrile seizure recurrence prevention in children with normal serum zinc level: A randomised clinical trial. Nutrition. 2015 Nov 1;31(11–12):1358–61.

22. Gupta DN, Mondal SK, Ghosh S, Rajendran K, Sur D, Manna B. Impact of zinc supplementation on diarrhoeal morbidity in rural children of West Bengal, India. Acta Paediatr Int J Paediatr. 2003 May 1;92(5):531–6.

23. Hambidge KM, Chavez MN, Brown RM, Walravens PA. Zinc nutritional status of young middle-income children and effects of consuming zinc-fortified breakfast cereals. Am J Clin Nutr. 1979;32(12):2532–9.

24. Hess SY, Abbeddou S, Jimenez EY, Somé JW, Vosti SA, Ouédraogo ZP, et al. Small-Quantity Lipid-Based Nutrient Supplements, Regardless of Their Zinc Content, Increase Growth and Reduce the Prevalence of Stunting and Wasting in Young Burkinabe Children: A Cluster-Randomized Trial. PLoS One. 2015;10(3):e0122242.

25. Kurugöl Z, Akilli M, Bayram N, Koturoglu G. The prophylactic and therapeutic effectiveness of zinc sulphate on common cold in children. Acta Paediatr [Internet]. 2006 Oct 1;95(10):1175–81. Available from: http://doi.wiley.com/10.1080/08035250600603024

26. Kurugöl Z, Bayram N, Atik T. Effect of zinc sulfate on common cold in children: Randomized, double blind study. Pediatr Int. 2007;49(6):842–7.

27. Larson CP, Hoque ABMM, Larson CP, Khan AM, Saha UR. Initiation of zinc treatment for acute childhood diarrhoea and risk for vomiting or regurgitation: A randomized, double-blind, placebo-controlled trial. J Heal Popul Nutr. 2005;23(4):311–9.

28. Lind T, Lönnerdal B, Stenlund H, Ismail D, Seswandhana R, Ekström E-C, et al. A community-based randomized controlled trial of iron and zinc supplementation in Indonesian infants: interactions between iron and zinc. Am J Clin Nutr [Internet]. 2003 Apr 1;77(4):883–90. Available from: https://academic.oup.com/ajcn/article/77/4/883/4689762

29. López De Romaña D, Salazar M, Hambidge KM, Penny ME, Peerson JM, Krebs NF, et al. Longitudinal measurements of zinc absorption in Peruvian children consuming wheat products fortified with iron only or iron and 1 of 2 amounts of zinc. Am J Clin Nutr. 2005;81(3):637–47.

30. Malik A, Taneja DK, Devasenapathy N, Rajeshwari K. Zinc supplementation for prevention of acute respiratory infections in infants: A randomized controlled trial. Indian Pediatr. 2014;51(10):780–4.

31. Martinez-Estevez NS, Alvarez-Guevara AN, Rodriguez-Martinez CE. Effects of zinc supplementation in the prevention of respiratory tract infections and diarrheal disease in Colombian children: A 12-month randomised controlled trial. Allergol Immunopathol (Madr) [Internet]. 2016 Jul 1;44(4):368–75. Available from: https://linkinghub.elsevier.com/retrieve/pii/S0301054616300192

32. Mendoza NJ, Del Y, Peña CB, Frank Papalé-Centofanti J, Torres-Villanueva M, Castro M. Anthropometric and biochemical nutritional status, parasitic infestation, social stratification. The effects of zinc supplementation in children of Venezuelan public kindergartens. Rev Esp Nutr Comunitaria. 2016;22(2).

33. Moradveisi B, Yazdanifard P, Naleini N, Sohrabi M. Comparison of iron alone and zinc plus iron supplementation effect on the clinical and laboratory features of children with iron deficiency anemia. Int J Hematol Stem Cell Res. 2019;13(4):220–8.

34. Muñoz EC, Rosado JL, López P, Furr HC, Allen LH. Iron and zinc supplementation improves indicators of vitamin A status of Mexican preschoolers. Am J Clin Nutr [Internet]. 2000 Mar 1;71(3):789–94. Available from: https://academic.oup.com/ajcn/article/71/3/789/4729207

35. Olney DK, Pollitt E, Kariger PK, Khalfan SS, Ali NS, Tielsch JM, et al. Combined iron and folic acid supplementation with or without zinc reduces time to walking unassisted among Zanzibari infants 5- to 11-mo old. J Nutr. 2006;136(9):2427–34.

36. Ouédraogo HZ, Dramaix-Wilmet M, Zeba AN, Hennart P, Donnen P. Effect of iron or multiple micronutrient supplements on the prevalence of anaemia among anaemic young children of a malaria-endemic area: A randomized double-blind trial. Trop Med Int Heal. 2008;13(10):1257–66.

37. Owusu-Agyei S, Newton S, Mahama E, Febir LG, Ali M, Adjei K, et al. Impact of vitamin A with zinc supplementation on malaria morbidity in Ghana. Nutr J. 2013;12(1):1–9.

38. Passariello A, Nocerino R, Terrin G, Cecere G, De Marco G, Micillo M, et al. Acceptability and efficacy of a gel hypotonic oral rehydration solution in children with acute gastroenteritis. Eur J Gastroenterol Hepatol [Internet]. 2015 May 14;27(5):523–6. Available from: https://journals.lww.com/00042737-201505000-00007

39. Radhakrishna K V, Hemalatha R, Geddam JJB, Kumar PA, Balakrishna N, Shatrugna V. Effectiveness of zinc supplementation to full term normal infants: a community based double blind, randomized, controlled, clinical trial. PLoS One [Internet]. 2013;8(5):e61486. Available from: http://www.ncbi.nlm.nih.gov/pubmed/23737940

40. Rahman MM, Vermund SH, Wahed MA, Fuchs GJ, Baqui AH, Alvarez JO. Simultaneous zinc and vitamin A supplementation in Bangladeshi children: randomised double blind controlled trial. BMJ [Internet]. 2001 Aug 11;323(7308):314–8. Available from: https://www.bmj.com/lookup/doi/10.1136/bmj.323.7308.314

41. Richard SA, Zavaleta N, Caulfield LE, Black RE, Witzig RS, Shankar AH. Zinc and iron supplementation and malaria, diarrhea, and respiratory infections in children in the Peruvian Amazon. Am J Trop Med Hyg. 2006;75(1):126–32.

42. Rosado JL, López P, Muñoz E, Martinez H, Allen LH. Zinc supplementation reduced morbidity, but neither zinc nor iron supplementation affected growth or body composition of Mexican preschoolers. Am J Clin Nutr. 1997;65(1):13–9.

43. Ryan KN, Stephenson KB, Trehan I, Shulman RJ, Thakwalakwa C, Murray E, et al. Zinc or albendazole attenuates the progression of environmental enteropathy: A randomized controlled trial. Clin Gastroenterol Hepatol [Internet]. 2014;12(9):1–8. Available from: http://dx.doi.org/10.1016/j.cgh.2014.01.024

44. Sampaio DLB, de Mattos ÂP, Ribeiro TCM, Leite ME de Q, Cole CR, Costa-Ribeiro H. Zinc and other micronutrients supplementation through the use of sprinkles: impact on the occurrence of diarrhea and respiratory infections in institutionalized children. J Pediatr (Rio J) [Internet]. 2013 May;89(3):286–93. Available from: https://www.cochranelibrary.com/central/doi/10.1002/central/CN-00959582/full

45. Sazawal S, Malik P, Jalla S, Krebs N, Bhan M, Black R. Zinc supplementation for four months does not affect plasma copper concentration in infants. Acta Paediatr [Internet]. 2004 May;93(5):599–602. Available from: https://ovidsp.ovid.com/ovidweb.cgi?T=JS&CSC=Y&NEWS=N&PAGE=fulltext&D=med5&AN=15174779

46. Sazawal S, Black RE, Ramsan M, Chwaya HM, Stoltzfus RJ, Dutta A, et al. Effects of routine prophylactic supplementation with iron and folic acid on admission to hospital and mortality in. Lancet [Internet]. 2006;367(9505):133–43. Available from: http://www.sciencedirect.com/science?_ob=GatewayURL&_origin=ScienceSearch&_method=citationSearch&_piikey=S0140673606679622&_version=1&_returnURL=&md5=59b88b4fa0756164e14fc63cc3316c5c

47. Shankar AH, Genton B, Baisor M, Jaino P, Tamja S, Adiguma T, et al. The influence of zinc supplementation on morbidity due to Plasmodium falciparum: A randomized trial in preschool children in Papua New Guinea. Am J Trop Med Hyg. 2000;62(6):663–9.

48. Silva APR, Vitolo MR, Zara LF, Castro CFS. Effects of zinc supplementation on 1- to 5-year old children. J Pediatr (Rio J). 2006;82(3):227–31.

49. Smith JC, Rao D, Makdani D, Hegar A, Douglass LW. Vitamin A and Zinc Supplementation of Preschool Children. J Am Coll Nutr. 1999;18(3):213–22.

50. Strand TA, Chandyo RK, Bahl R, Sharma PR, Adhikari RK, Bhandari N, et al. Effectiveness and Efficacy of Zinc for the Treatment of Acute Diarrhea in Young Children. Pediatrics [Internet]. 2002 May 1;109(5):898–903. Available from: http://pediatrics.aappublications.org/cgi/doi/10.1542/peds.109.5.898

51. Sugiura T, Goto K, Ito K, Ueta A, Fujimoto S, Togari H. Chronic zinc toxicity in an infant who received zinc therapy for atopic dermatitis. Acta Paediatr [Internet]. 2005 Sep 5;94(9):1333–5. Available from: https://ovidsp.ovid.com/ovidweb.cgi?T=JS&CSC=Y&NEWS=N&PAGE=fulltext&D=med6&AN=16203677

52. Surono IS, Martono PD, Kameo S, Suradji EW, Koyama H. Effect of probiotic L. plantarum IS-10506 and zinc supplementation on humoral immune response and zinc status of Indonesian pre-school children. J Trace Elem Med Biol [Internet]. 2014;28(4):465–9. Available from: http://dx.doi.org/10.1016/j.jtemb.2014.07.009

53. Valentiner-Branth P, Shrestha PS, Chandyo RK, Mathisen M, Basnet S, Bhandari N, et al. A randomized controlled trial of the effect of zinc as adjuvant therapy in children 2-35 mo of age with severe or nonsevere pneumonia in Bhaktapur, Nepal. Am J Clin Nutr. 2010 Jun 1;91(6):1667–74.

54. Veenemans J, Milligan P, Prentice AM, Schouten LRA, Inja N, van der Heijden AC, et al. Effect of Supplementation with Zinc and Other Micronutrients on Malaria in Tanzanian Children: A Randomised Trial. von Seidlein L, editor. PLoS Med [Internet]. 2011 Nov 22;8(11):e1001125. Available from: https://dx.plos.org/10.1371/journal.pmed.1001125

55. Walravens PA, Hambidge KM. Growth of infants fed a zinc supplemented formula. Am J Clin Nutr. 1976;29(10):1114–21.

56. Wasantwisut E, Winichagoon P, Chitchumroonchokchai C, Yamborisut U, Boonpraderm A, Pongcharoen T, et al. Iron and Zinc Supplementation Improved Iron and Zinc Status, but Not Physical Growth, of Apparently Healthy, Breast-Fed Infants in Rural Communities of Northeast Thailand. J Nutr [Internet]. 2006 Sep 1;136(9):2405–11. Available from: https://academic.oup.com/jn/article/136/9/2405/4664952

57. Wuehler SE, Sempértegui F, Brown KH. Dose-response trial of prophylactic zinc supplements, with or without copper, in young Ecuadorian children at risk of zinc deficiency. Am J Clin Nutr [Internet]. 2008 Mar 1;87(3):723–33. Available from: https://academic.oup.com/ajcn/article/87/3/723/4633435

58. Zlotkin S, Arthur P, Schauer C, Antwi KY, Yeung G, Piekarz A. Home-Fortification with Iron and Zinc Sprinkles or Iron Sprinkles Alone Successfully Treats Anemia in Infants and Young Children. J Nutr [Internet]. 2003 Apr 1;133(4):1075–80. Available from: https://academic.oup.com/jn/article/133/4/1075/4688097

59. Diop L, Becquey E, Turowska Z, Huybregts L, Ruel MT, Gelli A. Standard Minimum Dietary Diversity Indicators for Women or Infants and Young Children Are Good Predictors of Adequate Micronutrient Intakes in 24–59-Month-Old Children and Their Nonpregnant Nonbreastfeeding Mothers in Rural Burkina Faso. J Nutr [Internet]. 2021 Feb 1;151(2):412–22. Available from: https://academic.oup.com/jn/article/151/2/412/6039354

60. Instituto Nacional de Salud / Centro Nacional de Alimentación y Nutrición/Dirección Ejecutiva de Vigilancia Alimentaria y Nutricional. Informe Final: Encuesta Vigilancia Alimentaria y Nutricional por etapas de vida niños menores de 36 meses 2015. 2016; Available from: http://www.portal.ins.gob.pe/es/component/rsfiles/preview?path=cenan%2FVigilancia+de+Indicadores+Nutricionales+B%2FVIN+ENAHO+POBLACION+2013-2014+220116.pdf

61. Kimmons JE, Dewey KG, Haque E, Chakraborty J, Osendarp SJM, Brown KH. Low Nutrient Intakes among Infants in Rural Bangladesh Are Attributable to Low Intake and Micronutrient Density of Complementary Foods. J Nutr [Internet]. 2005 Mar 1;135(3):444–51. Available from: https://academic.oup.com/jn/article/135/3/444/4663669

62. Paul AA, Bates CJ, Prentice A, Day KC, Tsuchiya H. Zinc and phytate intake of rural Gambian infants: Contributions from breastmilk and weaning foods. Int J Food Sci Nutr. 1998;49(2):141–55.

63. Van Hoan N, Van Phu P, Salvignol B, Berger J, Trèche S. Effect of the consumption of high energy dense and fortified gruels on energy and nutrient intakes of 6–10-month-old Vietnamese infants. Appetite [Internet]. 2009 Oct;53(2):233–40. Available from: https://linkinghub.elsevier.com/retrieve/pii/S0195666309005637

64. Huey SL, Jiang L, Fedarko MW, McDonald D, Martino C, Ali F, et al. Nutrition and the Gut Microbiota in 10- to 18-Month-Old Children Living in Urban Slums of Mumbai, India. Young VB, editor. mSphere [Internet]. 2020 Oct 28;5(5). Available from: https://journals.asm.org/doi/10.1128/mSphere.00731-20

65. Maciel BLL, Costa PN, Filho JQ, Ribeiro SA, Rodrigues FAP, Soares AM, et al. Higher Energy and Zinc Intakes from Complementary Feeding Are Associated with Decreased Risk of Undernutrition in Children from South America, Africa, and Asia. J Nutr [Internet]. 2021 Jan 4;151(1):170–8. Available from: https://academic.oup.com/jn/article/151/1/170/5906637

66. Aga Khan University, Ministry of National Health Services, Regulations & Coordination (Pakistan), Ministry of Planning and Development (Pakistan), Pakistan Medical Research Council, United Nations Children’s Fund (UNICEF). Pakistan National Nutrition Survey 2011. Pakistan Natl Nutr Surv [Internet]. 2011;1–84. Available from: http://www.mhinnovation.net/sites/default/files/downloads/innovation/research/Pakistan National Nutrition Survey 2011.pdf

67. Sanin KI, Islam MM, Mahfuz M, Ahmed AMS, Mondal D, Haque R, et al. Micronutrient adequacy is poor, but not associated with stunting between 12-24 months of age: A cohort study findings from a slum area of Bangladesh. van Wouwe JP, editor. PLoS One [Internet]. 2018 Mar 29;13(3):e0195072. Available from: https://dx.plos.org/10.1371/journal.pone.0195072

68. Arsenault JE, López de Romaña D, Penny ME, Van Loan MD, Brown KH. Additional Zinc Delivered in a Liquid Supplement, but Not in a Fortified Porridge, Increased Fat-Free Mass Accrual among Young Peruvian Children with Mild-to-Moderate Stunting. J Nutr [Internet]. 2008 Jan 1;138(1):108–14. Available from: https://academic.oup.com/jn/article/138/1/108/4664978

69. Campbell RK, Hurley KM, Shamim AA, Shaikh S, Chowdhury ZT, Mehra S, et al. Complementary Food Supplements Increase Dietary Nutrient Adequacy and Do Not Replace Home Food Consumption in Children 6–18 Months Old in a Randomized Controlled Trial in Rural Bangladesh. J Nutr [Internet]. 2018 Sep 1;148(9):1484–92. Available from: https://academic.oup.com/jn/article/148/9/1484/5079788

70. Faber M. Complementary foods consumed by 6 – 12-month-old rural infants in South Africa are inadequate in micronutrients. Public Health Nutr [Internet]. 2005 Jun 2;8(4):373–81. Available from: https://www.cambridge.org/core/product/identifier/S1368980005000522/type/journal_article

71. Diana A, Mallard SR, Haszard JJ, Purnamasari DM, Nurulazmi I, Herliani PD, et al. Consumption of fortified infant foods reduces dietary diversity but has a positive effect on subsequent growth in infants from Sumedang district, Indonesia. Wieringa F, editor. PLoS One [Internet]. 2017 Apr 20;12(4):e0175952. Available from: https://dx.plos.org/10.1371/journal.pone.0175952

72. Ahmadi A, Moazen M, Mosallaei Z, Mohammadbeigi A, Amin-Iari F. Nutrient intake and growth indices for children at kindergartens in Shiraz, Iran. J Pak Med Assoc [Internet]. 2014 Mar;64(3):316–21. Available from: http://www.ncbi.nlm.nih.gov/pubmed/24864607

73. Kinabo J, Mamiro P, Mwanri A, Bundala N, Kulwa K, Picado J, et al. Adequacy of macro and micronutrients in infants and young children’s diets in Zanzibar, Tanzania. Afr Health Sci [Internet]. 2019 Jan 1;19(4):3063–77. Available from: https://www.ajol.info/index.php/ahs/article/view/192289

74. de Jager I, Borgonjen-van den Berg KJ, Giller KE, Brouwer ID. Current and potential role of grain legumes on protein and micronutrient adequacy of the diet of rural Ghanaian infants and young children: using linear programming. Nutr J [Internet]. 2019 Dec 21;18(1):12. Available from: https://nutritionj.biomedcentral.com/articles/10.1186/s12937-019-0435-5

75. Rodriguez C, Smith E, Villamor E, Zavaleta N, Respicio-Torres G, Contreras C, et al. Development and Validation of a Food Frequency Questionnaire to Estimate Intake among Children and Adolescents in Urban Peru. Nutrients [Internet]. 2017 Oct 14;9(10):1121. Available from: http://www.mdpi.com/2072-6643/9/10/1121

76. Mundo-Rosas V, Rodríguez-Ramírez S, Shamah-Levy T. Energy and nutrient intake in Mexican children 1 to 4 years old: results from the Mexican National Health and Nutrition Survey 2006. Salud Publica Mex [Internet]. 2009;51. Available from: http://www.scielo.br/scielo.php?script=sci_arttext&pid=S0036-36342009001000008&lng=en&nrm=iso&tlng=en

77. Gibson RS, Heywood A, Yaman C, Sohlström A, Thompson LU, Heywood P. Growth in children from the Wosera subdistrict, Papua New Guinea, in relation to energy and protein intakes and zinc status. Vol. 53, American Journal of Clinical Nutrition. 1991. p. 782–9.

78. Beinner MA, Menezes MÂ de BC, Silva JBB da, Amorim FR de, Jansen AK, Lamounier JA. Plasma zinc and hair zinc levels, anthropometric status and food intake of children in a rural area of Brazil. Rev Nutr [Internet]. 2010 Feb;23(1):75–83. Available from: http://www.scielo.br/scielo.php?script=sci_arttext&pid=S1415-52732010000100009&lng=en&tlng=en

79. Morseth MS, Torheim LE, Chandyo RK, Ulak M, Shrestha SK, Shrestha B, et al. Severely inadequate micronutrient intake among children 9–24 months in Nepal—The MAL‐ED birth cohort study. Matern Child Nutr [Internet]. 2018 Apr 2;14(2). Available from: https://onlinelibrary.wiley.com/doi/10.1111/mcn.12552

80. Kittisakmontri K, Lanigan J, Sangcakul A, Tim-Aroon T, Meemaew P, Wangaueattachon K, et al. Comparison of 24-Hour Recall and 3-Day Food Records during the Complementary Feeding Period in Thai Infants and Evaluation of Plasma Amino Acids as Markers of Protein Intake. Nutrients [Internet]. 2021 Feb 17;13(2):653. Available from: https://www.mdpi.com/2072-6643/13/2/653

81. Freire W., Ramírez M, Belmont P, Mendieta M, Silva M, Romero N, et al. RESUMEN EJECUTIVO. TOMO I. Encuesta Nacional de Salud y Nutrición del Ecuador. ENSANUT-ECU 2011-2013. Ministerio de Salud Pública/Instituto Nacional de Estadística y Censos., editor. Quito, Ecuador; 2013.

82. Zlotkin SH, Schauer C, Agyei SO, Wolfson J, Tondeur MC, Asante KP, et al. Demonstrating zinc and iron bioavailability from intrinsically labeled microencapsulated ferrous fumarate and zinc gluconate sprinkles in young children. J Nutr [Internet]. 2006 Apr 1;136(4):920–5. Available from: https://academic.oup.com/jn/article/136/4/920/4664283

1. Where zinc intake from breast milk is already included in the dietary intake column, where estimated dietary zinc intake likely exceeds the true intake due to differences in age of the study populations, or where children are aged >36 mo., no additional zinc intake from breast milk has been added to the total estimate; 2<60 µg/dL; 3<65 µg/dL; 4 <64 µg/dL; 5 <70 µg/ dL; NR= not reported. [↑](#footnote-ref-2)
2. Same study as Abbeddou et al. (1) but with additional data [↑](#footnote-ref-3)
3. Same study as Rosado et al. (42), but Rosado reports outcomes following 12 months supplementation [↑](#footnote-ref-4)
4. Same study as Sazawal et al. (46) but with additional data [↑](#footnote-ref-5)
5. Same study as Muñoz et al. (34) but Muñoz reports outcomes following 6 months supplementation [↑](#footnote-ref-6)
6. Same study as Olney et al. (35) different outcomes [↑](#footnote-ref-7)
7. Where zinc intake from breast milk is already included in the dietary intake column, where estimated dietary zinc intake likely exceeds the true intake due to differences in age of the study populations, or where children are aged >36 mo, no additional zinc intake from breast milk has been added to the total estimate [↑](#footnote-ref-8)
