# Supplementary material for: Adverse Effects of Excessive Zinc Intake in Infants and Children Aged 0–3 Years: A Systematic Review and Meta-Analysis
Source: Adv Nutr. 2022 Sep 2;13(6):2488–518. doi: 10.1093/advances/nmac088 (PMC9776731; doi:10.1093/advances/nmac088)
Supplement: nmac088_Supplemental_Files [file nmac088_supplemental_files.zip › Supplementary File 3 Risk of Bias, GRADE and JBI.docx]

**Adverse effects of excessive zinc intake in infants and children aged 0-3 years: A systematic review and meta-analysis.**

Supplementary File 3. Risk of Bias, GRADE and JBI

**Note:** Reference numbers pertain to the reference list at the end of this document.

Table of Contents

[Risk of Bias Summary 3](#_Toc109898512)

[Studies included in the meta-analyses 3](#_Toc109898513)

[Studies included in the narrative 4](#_Toc109898514)

[Risk of Bias Graph 5](#_Toc109898515)

[Studies included in the meta-analyses 5](#_Toc109898516)

[Studies in the narrative 5](#_Toc109898517)

[GRADE 6](#_Toc109898518)

[GRADE Evidence table: Hemoglobin concentration (g/L) 6](#_Toc109898519)

[GRADE Evidence table: Anemia 17](#_Toc109898520)

[GRADE Evidence table: Ferritin concentration (µg/L) 25](#_Toc109898521)

[GRADE Evidence table: Serum/plasma copper concentration (µg/dL) 34](#_Toc109898522)

[GRADE Evidence table: Iron deficiency 43](#_Toc109898523)

[GRADE Evidence table: Iron deficiency anemia 47](#_Toc109898524)

[GRADE Evidence table: Serum/soluble transferrin receptor concentration (mg/L) 50](#_Toc109898525)

[GRADE Evidence table: Hematocrit (proportion by volume of red blood cells in blood) 53](#_Toc109898526)

[GRADE Evidence table: Raised C-reactive protein 57](#_Toc109898527)

[GRADE Evidence table: Erythrocyte super oxide dismutase (IU/mg hemoglobin) 60](#_Toc109898528)

[GRADE Evidence table: Zinc protoporphyrin (µmol/mol heme) 62](#_Toc109898529)

[GRADE Evidence table: Serum total cholesterol (mg/dL) 64](#_Toc109898530)

[GRADE Evidence table: Lactulose:mannitol (molar ratio) 67](#_Toc109898531)

[GRADE Evidence table: Serum iron (µg/dL) 69](#_Toc109898532)

[Funnel plots to assess publication bias 72](#_Toc109898533)

[Hemoglobin (g/L) 72](#_Toc109898534)

[Anemia (odds ratio) 75](#_Toc109898535)

[Ferritin 79](#_Toc109898536)

[Serum/plasma copper (µg/dL) 83](#_Toc109898537)

[Iron deficiency (odds ratio) 85](#_Toc109898538)

[Iron deficiency anemia (odds ratio) 87](#_Toc109898539)

[Serum soluble transferrin receptor (sTfR) (mg/L) 89](#_Toc109898540)

[Joanna Briggs Institute Critical Appraisal 91](#_Toc109898541)

[Methodological Assessment of Case reports 91](#_Toc109898542)

[References 92](#_Toc109898543)

#
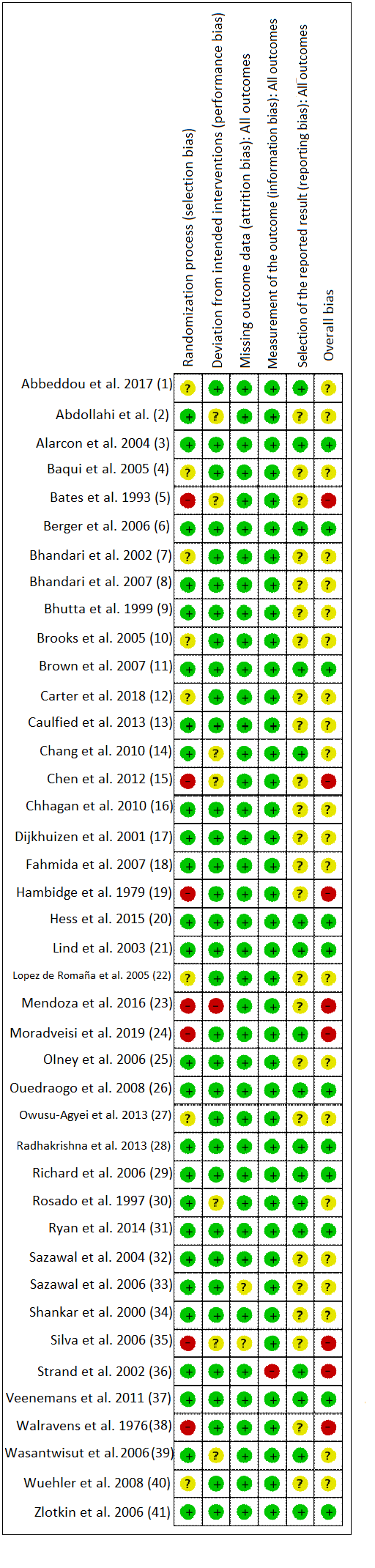
Risk of Bias Summary

## Studies included in the meta-analyses

Figure 1Risk of bias summary of studies included in meta-analysis: Report authors' judgements about each risk of bias item for each included study


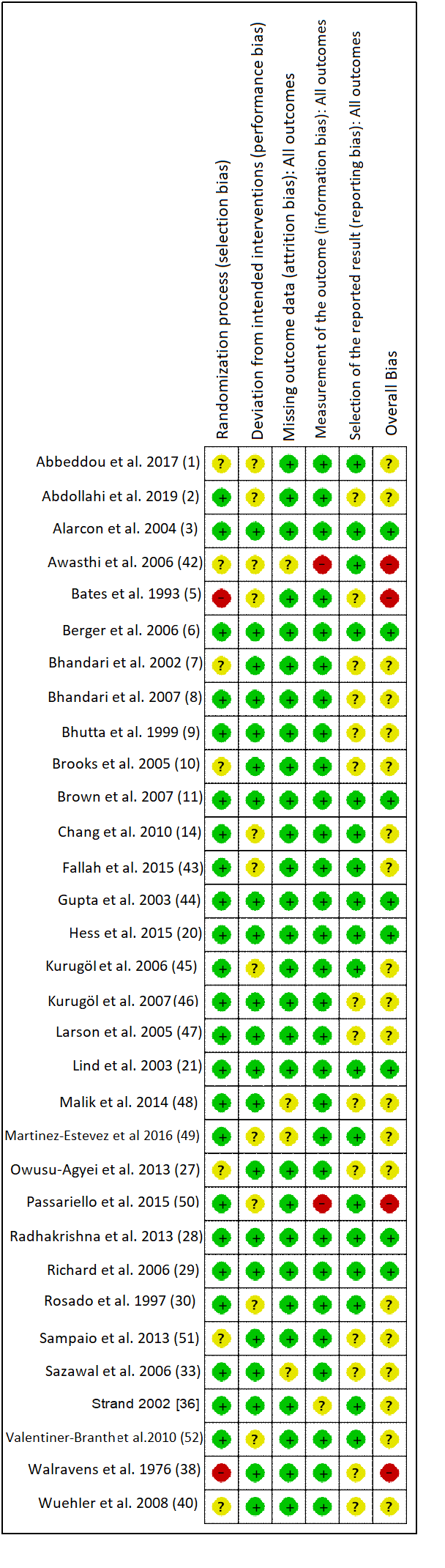


Studies included in the narrative

Figure 2 Risk of bias summary of studies included in the narrative: Report authors' judgements about each risk of bias item for each included study

# Risk of Bias Graph

## Studies included in the meta-analyses


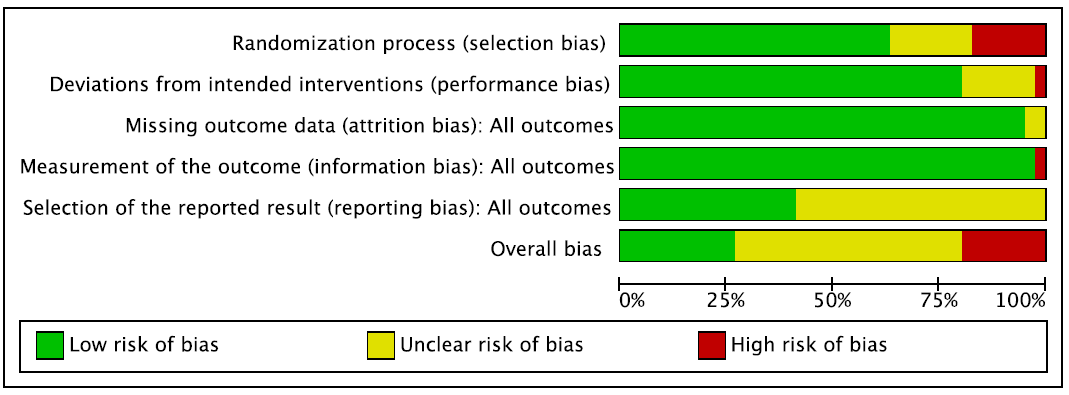


Figure 3 Risk of bias graph of studies included in the meta-analyses: report authors' judgements about each risk of bias item presented as percentages across all included studies. Overall bias is calculated according to the Cochrane RoB 2 algorithm (high risk if one or more categories has high risk or many categories have some concerns; some concerns if at least one category has some concerns, and low risk if all categories low risk).

## Studies in the narrative


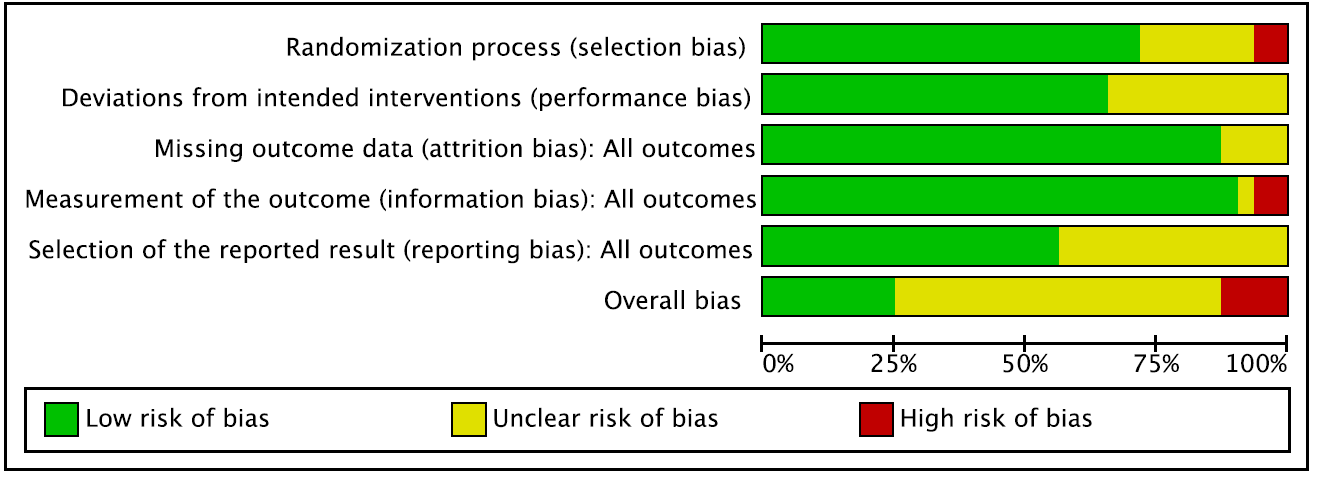


Figure 4 Risk of bias graph of studies included in the narrative: report authors' judgements about each risk of bias item presented as percentages across all included studies. Overall bias is calculated according to the Cochrane RoB 2 algorithm (high risk if one or more categories has high risk or many categories have some concerns; some concerns if at least one category has some concerns, and low risk if all categories low risk). (high risk if

# GRADE

## GRADE Evidence table: Hemoglobin concentration (g/L)

| **Certainty assessment** | | | | | | | **Summary of findings** | | | | | **Importance** |
| --- | --- | --- | --- | --- | --- | --- | --- | --- | --- | --- | --- | --- |
|  |  |  |  |  |  |  | **No of patients** | | **Effect** | | **Certainty** |  |
| **No of studies** | **Design** | **Risk of bias** | **Inconsistency** | **Indirectness** | **Imprecision** | **Other considerations** | **Intervention** | **Control** | **Relative (95% CI)** | **Absolute (95% CI)** |  |  |
| **Hemoglobin concentration (g/L): by age and dose** | | | | | | | | | | | | |
| *In children aged 0-90 days, <5 mg* | | | | | | | | | | | | |
| 1 | NRCT | serious^1^ | NA | not serious | serious^2^ | none | 34 | 34 | - | 1.00 g/L  (-7.17, 9.17) | ⊕⊕⊝⊝  LOW^a^ | Critical |
| *In children aged 0-90 days, 5-10 mg* | | | | | | | | | | | | |
| 1 (2 comparisons) | RCT | not serious | not serious | not serious | serious^2^ | none | 326 | 333 | - | 0.00 g/L  (-2.06, 2.06) | ⊕⊕⊕⊝  MODERATE^b^ | Critical |
| *In children aged 0-90 days, OVERALL* | | | | | | | | | | | | |
| 2 (3 comparisons) | RCT/ NRCT | serious^1^ | not serious | not serious | serious^2^ | none | 360 | 367 | - | 0.06 g/L  (-1.93, 2.05) | ⊕⊕⊝⊝  LOW^a^ | Critical |
| *In children aged 91-180 days, 5-10 mg/d* | | | | | | | | | | | | |
| 5 (8 comparisons) | RCT | not serious | not serious | serious^3^ | not serious | none | 862 | 878 | - | -2.39 g/L  (-3.94, -0.84) | ⊕⊕⊕⊝  MODERATE^c^ | Critical |
| *In children aged 91-180 days, bolus 21 mg/wk* | | | | | | | | | | | | |
| 1 | RCT | not serious | NA | not serious | serious^2^ | none | 329 | 309 | - | -1.00 g/L  (-2.44, 0.44) | ⊕⊕⊕⊝  MODERATE^b^ | Critical |
| *In children aged 91-180 days, OVERALL* | | | | | | | | | | | | |
| 6 (9 comparisons) | RCT | not serious | not serious | serious^3^ | not serious | none | 1191 | 1187 | - | -2.02 g/L  (-3.31, -0.72) | ⊕⊕⊕⊝  MODERATE^c^ | Critical |
| *In children aged 6–12 months, <5 mg/d* | | | | | | | | | | | | |
| 1 (2 comparisons) | RCT | not serious | not serious | serious^3^ | serious^2^ | none | 137 | 76 | - | 1.91 g/L  (-1.16, 4.98) | ⊕⊕⊕⊝  MODERATE^d^ | Critical |
| *In children aged 6–12 months, 5-10 mg/d* | | | | | | | | | | | | |
| 9 (15 comparisons) | RCT | not serious | serious^4^ | serious^5^ | serious^6^ | none | 2033 | 1953 | - | -0.02 g/L  (-1.60, 1.55) | ⊕⊝⊝⊝  VERY LOW^e^ | Critical |
| *In children aged 6-12 months, bolus 20 mg/wk* | | | | | | | | | | | | |
| 1 (2 comparisons) | RCT | not serious | not serious | not serious | serious^2^ | none | 121 | 128 | - | -2.87 g/L  (-6.13, 0.39) | ⊕⊕⊕⊝  MODERATE^b^ | Critical |
| *In children aged 6–12 months, OVERALL* | | | | | | | | | | | | |
| 11 (19 comparisons) | RCT | not serious | serious^4^ | serious^5^ | serious^6^ | publication bias not detected | 2291 | 2157 | - | -0.09 g/L  (-1.45, 1.27) | ⊕⊝⊝⊝  VERY LOW^e^ | Critical |
| *In children aged >12 months, <5 mg/d* | | | | | | | | | | | | |
| 3 | RCT/ NRCT | serious^7^ | not serious | serious^8^ | serious^2^ | none | 43 | 51 | - | -2.40 g/L  (-8.43, 3.64) | ⊕⊝⊝⊝  VERY LOW^f^ | Critical |
| *In children aged >12 months, 5-10 mg/d* | | | | | | | | | | | | |
| 7 (13 comparisons) | RCT | not serious | not serious | serious^9^ | serious^6^ | none | 731 | 730 | - | 0.74 g/L  (-0.98, 2.46) | ⊕⊕⊝⊝  LOW^g^ | Critical |
| *In children aged >12 months, 10.1-20 mg/d* | | | | | | | | | | | | |
| 4 (6 comparisons) | RCT | not serious | not serious | serious^10^ | serious^6^ | none | 618 | 600 | - | -0.09 g/L  (-1.28, 1.11) | ⊕⊕⊝⊝  LOW^h^ | Critical |
| *In children aged >12 months, bolus 140 mg/wk (70 mg twice weekly)* | | | | | | | | | | | | |
| 1 | NRCT | serious^1^ | NA | not serious | serious^2^ | none | 50 | 55 | - | 1.00 g/L  (-6.55, 8.55) | ⊕⊕⊝⊝  LOW^a^ | Critical |
| *In children aged >12 months, OVERALL* | | | | | | | | | | | | |
| 13 (23 comparisons) | RCT/ NRCT | not serious | not serious | serious^9^ | serious^6^ | publication bias suspected | 1442 | 1436 | - | 0.34 g/L  (-0.73, 1.41) | ⊕⊕⊝⊝  LOW^g^ | Critical |
| **Hemoglobin concentration (g/L): by treatment duration and dose** | | | | | | | | | | | | |
| *In children receiving interventions for < 3 months, < 5 mg/d* | | | | | | | | | | | | |
| 1 | RCT | not serious | NA | serious^3^ | serious^2^ | none | 5 | 6 | - | -5.14 g/L  (-24.21, 13.41) | ⊕⊕⊝⊝  LOW^d^ | Critical |
| *In children receiving interventions for < 3 months, 5-10 mg/d* | | | | | | | | | | | | |
| 2 (3 comparisons) | RCT | not serious | not serious | serious^11^ | serious^12^ | none | 136 | 126 | - | -5.26 g/L  (-8.98, -1.54) | ⊕⊕⊝⊝  LOW^i^ | Critical |
| *In children receiving interventions for < 3 months, 10.1-20 mg/d* | | | | | | | | | | | | |
| 1 | RCT | serious^1^ | NA | serious^13^ | serious^2^ | none | 48 | 40 | - | 0.40 g/L  (-3.67, 4.47) | ⊕⊝⊝⊝  VERY LOW^j^ | Critical |
| *In children receiving interventions for < 3 months, OVERALL* | | | | | | | | | | | | |
| 3 (5 comparisons) | RCT | serious^1^ | not serious | serious^14^ | serious^2^ | none | 189 | 172 | - | -3.13 g/L  (-6.82, 0.57) | ⊕⊝⊝⊝  VERY LOW^k^ | Critical |
| *In children receiving interventions for > 3 months, < 5 mg/d* | | | | | | | | | | | | |
| 4 (5 comparisons) | RCT/ NRCT | serious^7^ | not serious | serious^8^ | serious^2^ | none | 209 | 155 | - | 0.39 g/L  (-2.70, 3.48) | ⊕⊝⊝⊝  VERY LOW^f^ | Critical |
| *In children receiving interventions for > 3 months, 5-10 mg/d* | | | | | | | | | | | | |
| 20 (35 comparisons) | RCT | not serious | serious^4^ | serious^15^ | serious^6^ | none | 3815 | 3774 | - | -0.21 g/L  (-1.20, 0.77) | ⊕⊝⊝⊝  VERY LOW^l^ | Critical |
| *In children receiving interventions for > 3 months, 10.1-20 mg/d* | | | | | | | | | | | | |
| 3 (5 comparisons) | RCT | not serious | not serious | serious^16^ | serious^6^ | none | 570 | 560 | - | -0.13 g/L  (-1.39, 1.12) | ⊕⊕⊝⊝  LOW^m^ | Critical |
| *In children receiving interventions for > 3 months, bolus 20 mg/wk, 21 mg/wk, or 140 mg/wk (70 mg twice weekly)* | | | | | | | | | | | | |
| 3 (4 comparisons) | RCT/ NRCT | serious^1^ | not serious | not serious | serious^6^ | none | 500 | 492 | - | -1.24 g/L  (-2.53, 0.06) | ⊕⊕⊝⊝  LOW^n^ | Critical |
| *In children receiving interventions for > 3 months, OVERALL* | | | | | | | | | | | | |
| 29 (49 comparisons) | RCT/ NRCT | not serious | serious^4^ | serious^17^ | serious^6^ | publication bias not detected | 5094 | 4981 | - | -0.25 g/L  (-1.00, 0.50) | ⊕⊝⊝⊝  VERY LOW^o^ | Critical |
| **Hemoglobin concentration (g/L): by zinc form and dose** | | | | | | | | | | | | |
| *In children receiving zinc as zinc gluconate, 5-10 mg/d* | | | | | | | | | | | | |
| 5 | RCT | serious^1^ | serious^4^ | serious^15^ | serious^6^ | none | 449 | 433 | - | -1.26 g/L  (-4.19, 1.67) | ⊕⊝⊝⊝  VERY LOW^l^ | Critical |
| *In children receiving zinc as zinc gluconate, bolus 140 mg/wk (70 mg twice weekly)* | | | | | | | | | | | | |
| 1 | NRCT | serious^1^ | NA | not serious | serious^2^ | none | 50 | 55 | - | 1.00 g/L  (-6.55, 8.55) | ⊕⊕⊝⊝  LOW^a^ | Critical |
| *In children receiving zinc as zinc gluconate, OVERALL* | | | | | | | | | | | | |
| 6 | RCT/ NRCT | serious^1^ | serious^4^ | serious^15^ | serious^6^ | none | 499 | 488 | - | -1.01 g/L  (-3.63, 1.61) | ⊕⊝⊝⊝  VERY LOW^l^ | Critical |
| *In children receiving zinc as zinc sulphate, < 5 mg/d* | | | | | | | | | | | | |
| 4 (5 comparisons) | RCT/ NRCT | serious^1^ | not serious | serious^3^ | serious^2^ | none | 193 | 135 | - | 1.82 g/L  (-0.89, 4.53) | ⊕⊝⊝⊝  VERY LOW^p^ | Critical |
| *In children receiving zinc as zinc sulphate, 5-10 mg/d* | | | | | | | | | | | | |
| 12 (22 comparisons) | RCT | not serious | serious^4^ | serious^10^ | serious^6^ | none | 1796 | 1832 | - | -0.97 g/L  (-2.51, 0.56) | ⊕⊝⊝⊝  VERY LOW^q^ | Critical |
| *In children receiving zinc as zinc sulphate, 10.1-20 mg/d* | | | | | | | | | | | | |
| 2 (3 comparisons) | RCT | serious^1^ | not serious | serious^16^ | serious^6^ | none | 424 | 412 | - | -0.03 g/L  (-1.54, 1.49) | ⊕⊝⊝⊝  VERY LOW^r^ | Critical |
| *In children receiving zinc as zinc sulphate, OVERALL* | | | | | | | | | | | | |
| 16 (30 comparisons) | RCT/ NRCT | not serious | serious^4^ | serious^16^ | serious^6^ | publication bias suspected | 2413 | 2379 | - | -0.46 g/L  (-1.60, 0.68) | ⊕⊝⊝⊝  VERY LOW^s^ | Critical |
| *In children receiving zinc as zinc acetate (bolus 20 or 21 mg/wk), OVERALL* | | | | | | | | | | | | |
| 2 (3 comparisons) | RCT | not serious | not serious | not serious | serious^6^ | none | 450 | 437 | - | -1.30 g/L  (-2.62, 0.01) | ⊕⊕⊕⊝  MODERATE^t^ | Critical |
| *In children receiving an unstated form of zinc, 5-10 mg/d* | | | | | | | | | | | | |
| 5 (11 comparisons) | RCT | not serious | serious^4^ | serious^16^ | serious^6^ | none | 1706 | 1635 | - | 0.59 g/L  (-1.01, 2.18) | ⊕⊝⊝⊝  VERY LOW^u^ | Critical |
| *In children receiving an unstated form of zinc, 10.1-20 mg/d* | | | | | | | | | | | | |
| 1 | RCT | not serious | NA | serious^11^ | serious^2^ | none | 100 | 96 | - | -0.80 g/L  (-4.80, 3.20) | ⊕⊕⊝⊝  LOW^v^ | Critical |
| *In children receiving an unstated form of zinc, OVERALL* | | | | | | | | | | | | |
| 6 (12 comparisons) | RCT | not serious | serious^4^ | serious^18^ | serious^6^ | none | 1806 | 1731 | - | 0.47 g/L  (-1.01, 1.96) | ⊕⊝⊝⊝  VERY LOW^w^ | Critical |
| *In children receiving an ‘other’ form of zinc < 5 mg/d* | | | | | | | | | | | | |
| 1 | NRCT | serious^7^ | NA | serious^8^ | serious^2^ | none | 21 | 26 | - | -5.10 g/L  (-10.24, 0.04) | ⊕⊝⊝⊝  VERY LOW^f^ | Critical |
| *In children receiving an ‘other’ form of zinc 10.1-20 mg/d* | | | | | | | | | | | | |
| 1 (2 comparisons) | RCT | not serious | not serious | serious^3^ | serious^2^ | none | 94 | 92 | - | 0.00 g/L  (-2.24, 2.24) | ⊕⊕⊝⊝  LOW^d^ | Critical |
| *In children receiving an ‘other’ form of zinc, OVERALL* | | | | | | | | | | | | |
| 2 (3 comparisons) | RCT/ NRCT | serious^7^ | not serious | serious^8^ | serious^2^ | none | 115 | 118 | - | -1.06 g/L  (-3.73, 1.62) | ⊕⊝⊝⊝  VERY LOW^f^ | Critical |
| **Hemoglobin concentration (g/L): zinc versus placebo by dose** | | | | | | | | | | | | |
| *In children receiving < 5 mg/d* | | | | | | | | | | | | |
| 2 | RCT/ NRCT | serious^7^ | serious^19^ | serious^8^ | serious^2^ | none | 71 | 45 | - | -1.12 g/L  (-9.70, 7.47) | ⊕⊝⊝⊝  VERY LOW^x^ | Critical |
| *In children receiving 5-10 mg/d* | | | | | | | | | | | | |
| 14 (16 comparisons) | RCT | not serious | serious^4^ | serious^11^ | serious^6^ | none | 2329 | 1638 | - | 0.25 g/L  (-1.11, 1.61) | ⊕⊝⊝⊝  VERY LOW^y^ | Critical |
| *In children receiving 10.1-20 mg/d* | | | | | | | | | | | | |
| 2 | RCT | not serious | not serious | serious^16^ | serious^2^ | none | 238 | 235 | - | -0.18 g/L  (-2.09, 1.73) | ⊕⊕⊝⊝  LOW^z^ | Critical |
| *In children receiving bolus 20 mg/wk, 21 mg/wk or 140 mg/wk (70 mg twice weekly)* | | | | | | | | | | | | |
| 3 | RCT/ NRCT | serious^1^ | not serious | not serious | serious^6^ | none | 443 | 424 | - | -1.04 g/L  (-2.39, 0.31) | ⊕⊕⊝⊝  LOW^†^ | Critical |
| *In children receiving zinc versus placebo, OVERALL* | | | | | | | | | | | | |
| 20 (23 comparisons) | RCT/ NRCT | not serious | not serious | serious^16^ | serious^6^ | publication bias not detected | 3081 | 2342 | - | -0.10 g/L  (-1.10, 0.90) | ⊕⊕⊝⊝  LOW^‡^ | Critical |
| **Hemoglobin concentration (g/L): low versus high dose zinc** | | | | | | | | | | | | |
| *In children receiving low (3, 5, 7 mg) versus high dose zinc (7, 9, 10 mg/d), OVERALL* | | | | | | | | | | | | |
| 3 (5 comparisons) | RCT | not serious | not serious | serious^11^ | serious^2^ | none | 336 | 335 | - | 0.35 g/L  (-1.89, 2.58) | ⊕⊕⊝⊝  LOW^v^ | Critical |

**Explanations**

^1^High risk of bias in randomization process (selection bias)

^2^Wide confidence intervals including benefit and harm, small sample size

^3^Underlying malnutrition and anemia

^4^Large difference in point estimates, limited overlap in confidence intervals, I^2^ ≥50%

^5^Acute gastroenteritis/diarrhoea, underlying malnutrition, anemia and malaria, HIV

^6^Wide confidence intervals including benefit and harm

^7^High risk of bias in randomization process (selection bias) and deviations from intended interventions (performance bias)

^8^Underlying malnutrition and anemia, age range up to 6 years

^9^Underlying malnutrition, anemia and malaria, age range up to 6 years

^10^Underlying malnutrition and anemia, age range up to 59 months

^11^Underlying malnutrition, anemia and malaria

^12^Small sample size

^13^Underlying anemia, age range up to 48 months

^14^Underlying malnutrition, anemia and malaria, age range up to 48 months

^15^Acute gastroenteritis/diarrhoea, underlying malnutrition, anemia and malaria, HIV, age range up to 72 months

^16^Underlying malnutrition, anemia and malaria, age range up to 15 years

^17^Acute gastroenteritis/diarrhoea, underlying malnutrition, anemia and malaria, HIV, age range up to 15 years

^18^Underlying malnutrition, anemia and malaria, age range up to 60 months

^19^Large difference in point estimates, I^2^ ≥50%

^a^Downgraded one level for study limitations (selection bias) and one level for imprecision (wide confidence intervals including benefit and harm, small sample size)

^b^Downgraded one level for imprecision (wide confidence intervals including benefit and harm, small sample size)

^c^Downgraded one level for indirectness (underlying malnutrition and anemia)

^d^Downgraded one level indirectness (underlying malnutrition and anemia) and one level for (wide confidence intervals including benefit and harm, small sample size)

^e^Downgraded one level for inconsistency (large difference in point estimates, limited overlap in confidence intervals, I^2^ ≥50%), one level for indirectness (acute gastroenteritis/diarrhoea, underlying malnutrition, anemia and malaria, HIV) and one level for imprecision (wide confidence intervals including benefit and harm)

^f^Downgraded one level for study limitations (selection and performance bias), one level for indirectness (underlying malnutrition and anemia, age range up to 6 years) and one level for imprecision (wide confidence intervals including benefit and harm, small sample size)

^g^Downgraded one level for indirectness (underlying malnutrition, anemia and malaria, age range up to 6 years) and on level for imprecision (wide confidence intervals including benefit and harm). Not downgraded for study limitations in this case as most data (>90%) were from studies at low or unclear risk of bias. The possibility of publication bias was not excluded but was not considered sufficient to downgrade the evidence

^h^Downgraded one level for indirectness (underlying malnutrition and anemia, age range up to 59 months) and one level for imprecision (wide confidence intervals including benefit and harm). Not downgraded for study limitations in this case as most data (>90%) were from studies at low or unclear risk of bias

^i^Downgraded one level for indirectness (underlying malnutrition, anemia and malaria) and one level for imprecision (small sample size)

^j^Downgraded one level for study limitations (selection bias), one level for indirectness (underlying anemia, age range up to 48 months) and one level for imprecision (wide confidence intervals including benefit and harm, small sample size)

^k^Downgraded one level for study limitations (selection bias), one level for indirectness (underlying malnutrition, anemia and malaria, age range up to 48 months) and one level for imprecision (wide confidence intervals including benefit and harm, small sample size)

^l^Downgraded one level for inconsistency (large difference in point estimates, limited overlap in confidence intervals, I^2^ ≥50%), one level for indirectness (acute gastroenteritis/diarrhoea, underlying malnutrition, anemia and malaria, HIV, age range up to 72 months) and one level for imprecision (wide confidence intervals including benefit and harm). Not downgraded for study limitations in this case as most data (>90%) were from studies at low or unclear risk of bias

^m^Downgraded one level for indirectness (underlying malnutrition, anemia and malaria, age range up to 15 years) and one level for imprecision (wide confidence intervals including benefit and harm)

^n^Downgraded one level for study limitations (selection bias) and one level for imprecision (wide confidence intervals including benefit and harm)

^o^Downgraded one level for inconsistency (large difference in point estimates, limited overlap in confidence intervals, I^2^ ≥50%), one level for indirectness (acute gastroenteritis/diarrhoea, underlying malnutrition, anemia and malaria, HIV, age range up to 15 years) and one level for imprecision (wide confidence intervals including benefit and harm). Not downgraded for study limitations in this case as most data (>90%) were from studies at low or unclear risk of bias

^p^Downgraded one level for study limitations (selection bias), one level for indirectness (underlying malnutrition and anemia) and one level for imprecision (wide confidence intervals including benefit and harm, small sample size)

^q^Downgraded one level for inconsistency (large difference in point estimates, limited overlap in confidence intervals, I^2^ ≥50%), one level for indirectness (underlying malnutrition and anemia, age range up to 59 months) and one level for imprecision (wide confidence intervals including benefit and harm). Not downgraded for study limitations in this case as most data (>90%) were from studies at low or unclear risk of bias

^r^Downgraded one level for study limitations (selection bias), one level for indirectness (underlying malnutrition, anemia and malaria, age range up to 15 years) and one level for imprecision (wide confidence intervals including benefit and harm)

^s^Downgraded one level for inconsistency (large difference in point estimates, limited overlap in confidence intervals, I^2^ ≥50%), one level for indirectness (underlying malnutrition, anemia and malaria, age range up to 15 years) and one level for imprecision (wide confidence intervals including benefit and harm). Not downgraded for study limitations in this case as most data (>90%) were from studies at low or unclear risk of bias. The possibility of publication bias was not excluded but was not considered sufficient to downgrade the evidence

^t^Downgraded one level for imprecision (wide confidence intervals including benefit and harm)

^u^Downgraded one level for inconsistency (large difference in point estimates, limited overlap in confidence intervals, I^2^ ≥50%), one level for indirectness (underlying malnutrition, anemia and malaria, age range up to 15 years) and one level for imprecision (wide confidence intervals including benefit and harm)

^v^Downgraded one level for indirectness (underlying malnutrition, anemia and malaria) and one level for imprecision (wide confidence intervals including benefit and harm)

^w^Downgraded one level for inconsistency (large difference in point estimates, limited overlap in confidence intervals, I^2^ ≥50%), one level for indirectness (underlying malnutrition, anemia and malaria, age range up to 60 months) and one level for imprecision (wide confidence intervals including benefit and harm)

^x^Downgraded one level for study limitations (selection and performance bias), one level for inconsistency (large difference in point estimates, I^2^ ≥50%), one level for indirectness (underlying malnutrition and anemia, age range up to 6 years) and one level for imprecision (wide confidence intervals including benefit and harm, small sample size)

^y^Downgraded one level for inconsistency (large difference in point estimates, limited overlap in confidence intervals, I^2^ ≥50%), one level for indirectness (underlying malnutrition, anemia and malaria) and one level for imprecision (wide confidence intervals including benefit and harm). Not downgraded for study limitations in this case as most data (>90%) were from studies at low or unclear risk of bias

^z^Downgraded one level for indirectness (underlying malnutrition, anemia and malaria, age range up to 15 years) and one level for imprecision (wide confidence intervals including benefit and harm, small sample size)

^†^Downgraded one level for study limitations (selection bias) and one level for imprecision (wide confidence intervals including benefit and harm)

^‡^Downgraded one level for indirectness (underlying malnutrition, anemia and malaria, age range up to 15 years) and one level for imprecision (wide confidence intervals including benefit and harm). Not downgraded for study limitations in this case as most data (>90%) were from studies at low or unclear risk of bias

CI: confidence interval, NA: not applicable (as only 1 comparison), NRCT: non-randomized controlled trial, RCT: randomized controlled trial

**GRADE Working Group grades of evidence**
**High certainty:** We are very confident that the true effect lies close to that of the estimate of the effect
**Moderate certainty:** We are moderately confident in the effect estimate: The true effect is likely to be close to the estimate of the effect, but there is a possibility that it is substantially different
**Low certainty:** Our confidence in the effect estimate is limited: The true effect may be substantially different from the estimate of the effect
**Very low certainty:** We have very little confidence in the effect estimate: The true effect is likely to be substantially different from the estimate of effect

## GRADE Evidence table: Anemia

| **Certainty assessment** | | | | | | | **Summary of findings** | | | | | **Importance** |
| --- | --- | --- | --- | --- | --- | --- | --- | --- | --- | --- | --- | --- |
|  |  |  |  |  |  |  | **No of patients** | | **Effect** | | **Certainty** |  |
| **No of studies** | **Design** | **Risk of bias** | **Inconsistency** | **Indirectness** | **Imprecision** | **Other considerations** | **Intervention** | **Control** | **Relative**  **(95% CI)** | **Absolute** |  |  |
| **Anemia: by age and dose** | | | | | | | | | | | | |
| *In children aged 0-90 days (5-10 mg/d), OVERALL* | | | | | | | | | | | | |
| 1 | RCT | not serious | NA | not serious | serious^1^ | none | 573/669 | 598/718 | OR 1.20  (0.89, 1.60) | 86 per 100 in Zn vs 83 per 100 in control | ⊕⊕⊕⊝  MODERATE^a^ | Critical |
| *In children aged 91-180 days (5-10 mg/d), OVERALL* | | | | | | | | | | | | |
| 3 (6 comparisons) | RCT | not serious | serious^2^ | serious^3^ | serious^4^ | none | 237/675 | 207/694 | OR 1.40  (0.93, 2.10) | 35 per 100 in Zn vs 30 per 100 in control | ⊕⊝⊝⊝  VERY LOW^b^ | Critical |
| *In children aged 6–12 months, < 5 mg/d* | | | | | | | | | | | | |
| 1 (3 comparisons) | RCT | not serious | not serious | serious^3^ | serious^4^ | none | 87/137 | 56/76 | OR 0.62  (0.34, 1.15) | 64 per 100 in Zn vs 74 per 100 in control | ⊕⊕⊝⊝  LOW^c^ | Critical |
| *In children aged 6–12 months, 5-10 mg/d* | | | | | | | | | | | | |
| 6 (12 comparisons) | RCT | not serious | not serious | serious^5^ | serious^1^ | none | 1051/1564 | 984/1492 | OR 1.04  (0.85, 1.28) | 67 per 100 in Zn vs 66 per 100 in control | ⊕⊕⊝⊝  LOW^d^ | Critical |
| *In children aged 6–12 months, OVERALL* | | | | | | | | | | | | |
| 7 (14 comparisons) | RCT | not serious | not serious | serious^5^ | serious^1^ | publication bias not detected | 1138/1701 | 1040/1568 | OR 1.00 (0.82, 1.22) | 67 per 100 in Zn vs 66 per 100 in control | ⊕⊕⊝⊝  LOW^d^ | Critical |
| *In children aged > 12 months, < 5 mg/d* | | | | | | | | | | | | |
| 2 | RCT/ NRCT | serious^6^ | not serious | serious^7^ | serious^4^ | none | 6/26 | 2/32 | OR 3.87  (0.81, 18.45 | 23 per 100 in Zn vs 6 per 100 in control | ⊕⊝⊝⊝  VERY LOW^e^ | Critical |
| *In children aged > 12 months, 5-10 mg/d* | | | | | | | | | | | | |
| 4 (6 comparisons) | RCT | serious^8^ | not serious | serious^9^ | serious^4^ | none | 212/490 | 217/492 | OR 0.97  (0.74, 1.29) | 43 per 100 in Zn vs 44 per 100 in control | ⊕⊝⊝⊝  VERY LOW^f^ | Critical |
| *In children aged > 12 months, 10.1-20 mg/d* | | | | | | | | | | | | |
| 1 | RCT | not serious | NA | serious^10^ | serious^4^ | none | 61/100 | 57/96 | OR 1.07  (0.60, 1.90) | 61 per 100 in Zn vs 59 per 100 in control | ⊕⊕⊝⊝  LOW^g^ | Critical |
| *In children aged >12 months, OVERALL* | | | | | | | | | | | | |
| 6 (9 comparisons) | RCT/ NRCT | serious^8^ | not serious | serious^9^ | serious^4^ | none | 279/616 | 276/620 | OR 1.03 (0.80, 1.31) | 45 per 100 in Zn vs 45 per 100 in control | ⊕⊝⊝⊝  VERY LOW^f^ | Critical |
| **Anemia: by treatment duration and dose** | | | | | | | | | | | | |
| *In children receiving interventions for 0–3 months, < 5 mg/d* | | | | | | | | | | | | |
| 1 | RCT | not serious | NA | serious^3^ | serious^4^ | none | 1/5 | 0/6 | OR 4.33  (0.14, 132.32) | 20 per 100 in Zn vs 0 per 100 in control | ⊕⊝⊝⊝  VERY LOW^h^ | Critical |
| *In children receiving interventions for 0–3 months, 5-10 mg/d* | | | | | | | | | | | | |
| 2 (3 comparisons) | RCT | not serious | not serious | serious^10^ | serious^11^ | none | 48/136 | 30/126 | OR 1.74  (1.01, 2.98) | 35 per 100 in Zn vs 24 per 100 in control | ⊕⊕⊝⊝  LOW^i^ | Critical |
| *In children receiving interventions for 0–3 months, OVERALL* | | | | | | | | | | | | |
| 2 (4 comparisons) | RCT | not serious | not serious | serious^10^ | serious^11^ | none | 49/141 | 30/132 | OR 1.78  (1.04, 3.03) | 35 per 100 in Zn vs 23 per 100 in control | ⊕⊕⊝⊝  LOW^i^ | Critical |
| *In children receiving interventions for > 3 months, < 5 mg/d* | | | | | | | | | | | | |
| 2 (3 comparisons) | RCT/ NRCT | serious^6^ | not serious | serious^7^ | serious^4^ | none | 92/158 | 58/102 | OR 0.85  (0.37, 1.95) | 58 per 100 in Zn vs 57 per 100 in control | ⊕⊝⊝⊝  VERY LOW^e^ | Critical |
| *In children receiving interventions for > 3 months, 5-10 mg/d* | | | | | | | | | | | | |
| 12 (22 comparisons) | RCT | not serious | not serious | serious^12^ | serious^1^ | none | 2025/3262 | 1976/3270 | OR 1.08  (0.93, 1.25) | 62 per 100 in Zn vs 60 per 100 in control | ⊕⊕⊝⊝  LOW^j^ | Critical |
| *In children receiving interventions for > 3 months, 10.1-20 mg/d* | | | | | | | | | | | | |
| 1 | RCT | not serious | NA | serious^10^ | serious^4^ | none | 61/100 | 57/96 | OR 1.07  (0.60, 1.90) | 61 per 100 in Zn vs 59 per 100 in control | ⊕⊕⊝⊝  LOW^g^ | Critical |
| *In children receiving interventions for > 3 months, OVERALL* | | | | | | | | | | | | |
| 15 (26 comparisons) | RCT/ NRCT | not serious | not serious | serious^13^ | not serious | publication bias suspected | 2178/3520 | 2091/3468 | OR 1.06 (0.92, 1.22) | 62 per 100 in Zn vs 60 per 100 in control | ⊕⊕⊕⊝  MODERATE^k^ | Critical |
| **Anemia: by zinc form and dose** | | | | | | | | | | | | |
| *In children receiving zinc as zinc gluconate (5-10 mg/d), OVERALL* | | | | | | | | | | | | |
| 4 | RCT | serious^8^ | not serious | serious^12^ | serious^4^ | none | 123/376 | 109/363 | OR 1.10 (0.71, 1.72) | 33 per 100 in Zn vs 30 per 100 in control | ⊕⊝⊝⊝  VERY LOW^l^ | Critical |
| *In children receiving zinc as zinc sulphate, < 5 mg/d* | | | | | | | | | | | | |
| 2 (3 comparisons) | RCT | not serious | not serious | serious^3^ | serious^4^ | none | 88/142 | 56/82 | OR 0.66  (0.36, 1.21) | 62 per 100 in Zn vs 68 per 100 in control | ⊕⊕⊝⊝  LOW^h^ | Critical |
| *In children receiving zinc as zinc sulphate, 5-10 mg/d* | | | | | | | | | | | | |
| 6 (11 comparisons) | RCT | not serious | not serious | serious^3^ | serious^1^ | none | 911/1626 | 903/1702 | OR 1.28  (0.98, 1.67) | 56 per 100 in Zn vs 53 per 100 in control | ⊕⊕⊝⊝  LOW^m^ | Critical |
| *In children receiving zinc as zinc sulphate, OVERALL* | | | | | | | | | | | | |
| 7 (14 comparisons) | RCT | not serious | not serious | serious^3^ | serious^1^ | publication bias not detected | 999/1768 | 959/1784 | OR 1.18  (0.92, 1.53) | 56 per 100 in Zn vs 54 per 100 in control | ⊕⊕⊝⊝  LOW^m^ | Critical |
| *In children receiving an unstated form of zinc, 5-10 mg/d* | | | | | | | | | | | | |
| 4 (10 comparisons) | RCT | not serious | not serious | serious^14^ | serious^1^ | none | 1039/1396 | 994/1331 | OR 0.97 (0.81, 1.15) | 74 per 100 in Zn vs 75 per 100 in control | ⊕⊕⊝⊝  LOW^n^ | Critical |
| *In children receiving an unstated form of zinc, 10.1-20 mg/d* | | | | | | | | | | | | |
| 1 | RCT | not serious | NA | serious^10^ | serious^4^ | none | 61/100 | 57/96 | OR 1.07  (0.60, 1.90) | 61 per 100 in Zn vs 59 per 100 in control | ⊕⊕⊝⊝  LOW^g^ | Critical |
| *In children receiving an unstated form of zinc, OVERALL* | | | | | | | | | | | | |
| 5 (11 comparisons) | RCT | not serious | not serious | serious^14^ | serious^1^ | publication bias not detected | 1100/1496 | 1051/1427 | OR 0.97 (0.82, 1.15) | 74 per 100 in Zn vs 74 per 100 in control | ⊕⊕⊝⊝  LOW^n^ | Critical |
| *In children receiving an ‘other’ form of zinc (zinc lactate), OVERALL* | | | | | | | | | | | | |
| 1 | NRCT | serious^6^ | NA | serious^7^ | serious^4^ | none | 5/21 | 2/26 | OR 3.75 (0.65, 21.74) | 24 per 100 in Zn vs 8 per 100 in control | ⊕⊝⊝⊝  VERY LOW^o^ | Critical |
| **Anemia: zinc versus placebo by dose** | | | | | | | | | | | | |
| *In children receiving zinc versus placebo, < 5 mg/d* | | | | | | | | | | | | |
| 1 | NRCT | serious^6^ | NA | serious^7^ | serious^4^ | none | 5/21 | 2/26 | OR 3.75 (0.65, 21.74) | 24 per 100 in Zn vs 8 per 100 in control | ⊕⊝⊝⊝  VERY LOW^o^ | Critical |
| *In children receiving zinc versus placebo, 5-10 mg/d* | | | | | | | | | | | | |
| 9 (10 comparisons) | RCT | not serious | not serious | serious^14^ | serious^1^ | none | 1210/1837 | 857/1356 | OR 0.89 (0.75, 1.05) | 66 per 100 in Zn vs 63 per 100 in control | ⊕⊕⊝⊝  LOW^n^ | Critical |
| *In children receiving zinc versus placebo, OVERALL* | | | | | | | | | | | | |
| 10 (11 comparisons) | RCT/ NRCT | not serious | not serious | serious^9^ | serious^1^ | publication bias not detected | 1215/1858 | 859/1382 | OR 0.90 (0.76, 1.06) | 65 per 100 in Zn vs 62 per 100 in control | ⊕⊕⊝⊝  LOW^p^ | Critical |
| **Anemia: low versus high dose zinc** | | | | | | | | | | | | |
| *In children receiving low versus high dose zinc (5 mg vs 10 mg/d), OVERALL* | | | | | | | | | | | | |
| 2 | RCT | not serious | not serious | serious^10^ | serious^4^ | none | 394/501 | 391/508 | OR 1.13 (0.83, 1.53) | 79 per 100 in Zn vs 77 per 100 in control | ⊕⊕⊝⊝  LOW^g^ | Critical |

**Explanations**

^1^Wide confidence intervals including benefit and harm

^2^Large difference in point estimates, I^2^ ≥50%

^3^Underlying malnutrition and anemia

^4^Wide confidence intervals including benefit and harm, small number of events

^5^Underlying malnutrition, anemia, malaria and HIV

^6^High risk of bias in randomization process (selection bias) and deviations from intended interventions (performance bias)

^7^Underlying malnutrition and anemia, age range up to 6 years

^8^High risk of bias in randomization process (selection bias)

^9^Underlying malnutrition, anemia and malaria, age range up to 72 months

^10^Underlying malnutrition, anemia and malaria

^11^Wide confidence intervals, small number of events

^12^Underlying malnutrition, anemia, malaria and HIV, age range up to 72 months

^13^Underlying malnutrition, anemia, malaria and HIV, age range up to 6 years

^14^Underlying malnutrition, anemia and malaria, age range up to 60 months

^a^Downgraded one level for imprecision (wide confidence intervals including benefit and harm)

^b^Downgraded one level for inconsistency (large difference in point estimates, I^2^ ≥50%), one level for indirectness (underlying malnutrition and anemia) and one level for imprecision (wide confidence intervals including benefit and harm, small number of events)

^c^Downgraded one level for indirectness (underlying malnutrition and anemia) and one level for imprecision (wide confidence intervals including benefit and harm, small number of events)

^d^Downgraded one level for indirectness (underlying malnutrition, anemia, malaria and HIV) and one level for imprecision (wide confidence intervals including benefit and harm)

^e^Downgraded one level for study limitations (selection and performance bias), one level for indirectness (underlying malnutrition and anemia, age range up to 6 years) and one level for imprecision (wide confidence intervals including benefit and harm, small number of events)

^f^Downgraded one level for study limitations (selection bias), one level for indirectness (underlying malnutrition, anemia and malaria, age range up to 72 months) and one level for imprecision (wide confidence intervals including benefit and harm, small number of events)

^g^Downgraded one level for indirectness (underlying malnutrition, anemia and malaria) and one level for imprecision (wide confidence intervals including benefit and harm, small number of events)

^h^Downgraded one level for indirectness (underlying malnutrition and anemia) and two levels for imprecision (very wide confidence intervals including benefit and harm, very small number of events)

^i^Downgraded one level for indirectness (underlying malnutrition, anemia and malaria) and one level for imprecision (wide confidence intervals, small number of events)

^j^Downgraded one level for indirectness (underlying malnutrition, anemia, malaria and HIV, age range up to 72 months) and one level for imprecision (wide confidence intervals including benefit and harm). Not downgraded for study limitations in this case as most data (>90%) were from studies at low or unclear risk of bias

^k^Downgraded one level for indirectness (underlying malnutrition, anemia, malaria and HIV, age range up to 6 years). Not downgraded for study limitations in this case as most data (>90%) were from studies at low or unclear risk of bias. The possibility of publication bias was not excluded but was not considered sufficient to downgrade the evidence

^l^Downgraded one level for study limitations (selection bias), one level for indirectness (underlying malnutrition, anemia, malaria and HIV, age range up to 72 months) and one level for imprecision (wide confidence intervals including benefit and harm, small number of events)

^m^Downgraded one level for indirectness (underlying malnutrition and anemia) and one level for imprecision (wide confidence intervals including benefit and harm)

^n^Downgraded one level for indirectness (underlying malnutrition, anemia and malaria, age range up to 60 months) and one level for imprecision (wide confidence intervals including benefit and harm)

^o^Downgraded one level for study limitations (selection and performance bias), one level for indirectness (underlying malnutrition and anemia, age range up to 6 years) and two levels for imprecision (very wide confidence intervals including benefit and harm, very small number of events)

^p^Downgraded one level for indirectness (underlying malnutrition, anemia and malaria, age range up to 72 months) and one level for imprecision (wide confidence intervals including benefit and harm). Not downgraded for study limitations in this case as most data (>90%) were from studies at low or unclear risk of bias

CI: confidence interval, NA: not applicable (as only 1 comparison), NRCT: non-randomized controlled trial, RCT: randomized controlled trial, OR: odds ratio

**GRADE Working Group grades of evidence**
**High certainty:** We are very confident that the true effect lies close to that of the estimate of the effect
**Moderate certainty:** We are moderately confident in the effect estimate: The true effect is likely to be close to the estimate of the effect, but there is a possibility that it is substantially different
**Low certainty:** Our confidence in the effect estimate is limited: The true effect may be substantially different from the estimate of the effect
**Very low certainty:** We have very little confidence in the effect estimate: The true effect is likely to be substantially different from the estimate of effect

## GRADE Evidence table: Ferritin concentration (µg/L)

| **Certainty assessment** | | | | | | | **Summary of findings** | | | | | **Importance** |
| --- | --- | --- | --- | --- | --- | --- | --- | --- | --- | --- | --- | --- |
|  |  |  |  |  |  |  | **No of patients** | | **Effect** | | **Certainty** |  |
| **No of studies** | **Design** | **Risk of bias** | **Inconsistency** | **Indirectness** | **Imprecision** | **Other considerations** | **Intervention** | **Control** | **Relative (95% CI)** | **Absolute (95% CI)** |  |  |
| **Ferritin concentration (µg/L): by age and dose** | | | | | | | | | | | | |
| *In children aged 0-90 days (5-10 mg/d), OVERALL* | | | | | | | | | | | | |
| 1 (2 comparisons) | RCT | not serious | serious^1^ | not serious | serious^2^ | none | 295 | 298 | - | 1.50 µg/L  (-0.65, 3.66) | ⊕⊕⊝⊝  LOW^a^ | Critical |
| *In children aged 91-180 days (5-10 mg/d), OVERALL* | | | | | | | | | | | | |
| 4 (7 comparisons) | RCT | not serious | serious^3^ | serious^4^ | not serious | none | 659 | 679 | - | -3.50 µg/L  (-6.31, -0.69) | ⊕⊕⊝⊝  LOW^b^ | Critical |
| *In children aged 6–12 months, < 5 mg/d* | | | | | | | | | | | | |
| 1 (2 comparisons) | RCT | not serious | not serious | serious^4^ | serious^2^ | none | 137 | 76 | - | -4.32 µg/L  (-12.86, 4.22) | ⊕⊕⊝⊝  LOW^c^ | Critical |
| *In children aged 6–12 months, 5-10 mg/d* | | | | | | | | | | | | |
| 4 (7 comparisons) | RCT | not serious | serious^1^ | serious^6^ | serious^5^ | none | 906 | 897 | - | -6.27 µg/L  (-14.95, 2.41) | ⊕⊝⊝⊝  VERY LOW^d^ | Critical |
| *In children aged 6–12 months, bolus 20 mg/wk* | | | | | | | | | | | | |
| 1 (2 comparisons) | RCT | not serious | not serious | not serious | serious^2^ | none | 81 | 75 | - | 5.35 µg/L  (-2.68, 13.39) | ⊕⊕⊕⊝  MODERATE^e^ | Critical |
| *In children aged 6–12 months, OVERALL* | | | | | | | | | | | | |
| 6 (11 comparisons) | RCT | not serious | serious^1^ | serious^6^ | serious^5^ | publication bias not detected | 1124 | 1048 | - | -3.73 µg/L  (-10.64, 3.18) | ⊕⊝⊝⊝  VERY LOW^d^ | Critical |
| *In children aged >12 months, < 5 mg/d* | | | | | | | | | | | | |
| 2 | RCT/ NRCT | serious^7^ | not serious | serious^8^ | serious^2^ | none | 26 | 32 | - | 2.89 µg/L  (-9.50, 15.28) | ⊕⊝⊝⊝  VERY LOW^f^ | Critical |
| *In children aged >12 months, 5-10 mg/d* | | | | | | | | | | | | |
| 5 (7 comparisons) | RCT | not serious | serious^1^ | serious^9^ | serious^5^ | none | 569 | 557 | - | 1.01 µg/L  (-0.85, 2.87) | ⊕⊝⊝⊝  VERY LOW^g^ | Critical |
| *In children aged >12 months, 10.1-20 mg/d* | | | | | | | | | | | | |
| 2 (3 comparisons) | RCT | serious^10^ | not serious | serious^4^ | serious^2^ | none | 142 | 132 | - | -1.18 µg/L  (-4.21, 1.85) | ⊕⊝⊝⊝  VERY LOW^h^ | Critical |
| *In children aged >12 months, bolus 140 mg/wk (70 mg twice weekly)* | | | | | | | | | | | | |
| 1 | NRCT | serious^10^ | NA | not serious | serious^2^ | none | 40 | 45 | - | 1.30 µg/L  (-5.84, 8.44) | ⊕⊕⊝⊝  LOW^i^ | Critical |
| *In children aged >12 months, OVERALL* | | | | | | | | | | | | |
| 10 (13 comparisons) | RCT/ NRCT | serious^7^ | serious^1^ | serious^8^ | serious^5^ | publication bias not detected | 777 | 766 | - | 0.55 µg/L  (-0.98, 2.09) | ⊕⊝⊝⊝  VERY LOW^j^ | Critical |
| **Ferritin concentration (µg/L): by treatment duration and dose** | | | | | | | | | | | | |
| *In children receiving interventions for < 3 months, < 5 mg/d* | | | | | | | | | | | | |
| 1 | RCT | not serious | NA | serious^4^ | serious^2^ | none | 5 | 6 | - | -3.00 µg/L  (-22.84, 16.84) | ⊕⊝⊝⊝  VERY LOW^k^ | Critical |
| *In children receiving interventions for < 3 months, 5-10 mg/d* | | | | | | | | | | | | |
| 1 (2 comparisons) | RCT | not serious | not serious | serious^4^ | serious^2^ | none | 12 | 11 | - | -3.79 µg/L  (-16.21, 8.63) | ⊕⊝⊝⊝  VERY LOW^k^ | Critical |
| *In children receiving interventions for < 3 months, 10.1-20 mg/d* | | | | | | | | | | | | |
| 1 | RCT | serious^10^ | NA | serious^11^ | serious^2^ | none | 48 | 40 | - | -0.10 µg/L  (-3.85, 3.65) | ⊕⊝⊝⊝  VERY LOW^l^ | Critical |
| *In children receiving interventions for < 3 months, OVERALL* | | | | | | | | | | | | |
| 2 (4 comparisons) | RCT | serious^10^ | not serious | serious^4^ | serious^2^ | none | 65 | 57 | - | -0.49 µg/L  (-4.02, 3.04) | ⊕⊝⊝⊝  VERY LOW^m^ | Critical |
| *In children receiving interventions for > 3 months, < 5 mg/d* | | | | | | | | | | | | |
| 2 (3 comparisons) | RCT/ NRCT | serious^7^ | not serious | serious^8^ | serious^2^ | none | 158 | 102 | - | -1.85 µg/L  (-9.37, 5.67) | ⊕⊝⊝⊝  VERY LOW^f^ | Critical |
| *In children receiving interventions for > 3 months, 5-10 mg/d* | | | | | | | | | | | | |
| 13 (21 comparisons) | RCT | not serious | serious^1^ | serious^9^ | serious^5^ | none | 2405 | 2420 | - | -2.32 µg/L  (-5.18, 0.53) | ⊕⊝⊝⊝  VERY LOW^g^ | Critical |
| *In children receiving interventions for > 3 months, 10.1-20 mg/d* | | | | | | | | | | | | |
| 1 (2 comparisons) | RCT | not serious | not serious | serious^4^ | serious^2^ | none | 94 | 92 | - | -3.23 µg/L  (-8.40, 1.94) | ⊕⊕⊝⊝  LOW^c^ | Critical |
| *In children receiving interventions for > 3 months, bolus 20 mg/wk or 140 mg/wk (70 mg twice weekly)* | | | | | | | | | | | | |
| 2 (3 comparisons) | RCT/ NRCT | serious^10^ | not serious | not serious | serious^2^ | none | 121 | 120 | - | 3.09 µg/L  (-2.25, 8.43) | ⊕⊕⊝⊝  LOW^i^ | Critical |
| *In children receiving interventions for > 3 months, OVERALL* | | | | | | | | | | | | |
| 19 (29 comparisons) | RCT/ NRCT | not serious | serious^1^ | serious^8^ | serious^5^ | publication bias not detected | 2778 | 2734 | - | -1.80 µg/L  (-4.31, 0.72) | ⊕⊝⊝⊝  VERY LOW^n^ | Critical |
| **Ferritin concentration (µg/L): by zinc form and dose** | | | | | | | | | | | | |
| *In children receiving zinc as zinc gluconate (bolus 140 mg/wk (70 mg twice weekly), OVERALL* | | | | | | | | | | | | |
| 1 | NRCT | serious^10^ | NA | not serious | serious^2^ | none | 40 | 45 | - | 1.30 µg/L  (-5.84, 8.44) | ⊕⊕⊝⊝  LOW^i^ | Critical |
| *In children receiving zinc as zinc sulphate < 5 mg/d* | | | | | | | | | | | | |
| 2 (3 comparisons) | RCT | not serious | not serious | serious^4^ | serious^2^ | none | 142 | 82 | - | -4.11 µg/L  (-11.95, 3.73) | ⊕⊕⊝⊝  LOW^c^ | Critical |
| *In children receiving zinc as zinc sulphate 5-10 mg/d* | | | | | | | | | | | | |
| 11 (17 comparisons) | RCT | not serious | serious^1^ | serious^6^ | serious^5^ | none | 1569 | 1597 | - | -2.50 µg/L  (-6.69, 1.70) | ⊕⊝⊝⊝  VERY LOW^d^ | Critical |
| *In children receiving zinc as zinc sulphate, 10.1-20 mg/d* | | | | | | | | | | | | |
| 1 | RCT | serious^10^ | NA | serious^11^ | serious^2^ | none | 48 | 40 | - | -0.10 µg/L  (-3.85, 3.65) | ⊕⊝⊝⊝  VERY LOW^l^ | Critical |
| *In children receiving zinc as zinc sulphate, OVERALL* | | | | | | | | | | | | |
| 13 (21 comparisons) | RCT | not serious | serious^1^ | serious^6^ | serious^5^ | publication bias suspected | 1759 | 1719 | - | -2.48 µg/L  (-6.30, 1.34) | ⊕⊝⊝⊝  VERY LOW^o^ | Critical |
| *In children receiving zinc as zinc acetate (10.1-20 mg/d), OVERALL* | | | | | | | | | | | | |
| 1 (2 comparisons) | RCT | not serious | not serious | not serious | serious^2^ | none | 81 | 75 | - | 5.35 µg/L  (-2.68, 13.39) | ⊕⊕⊕⊝  MODERATE^e^ | Critical |
| *In children receiving an unstated form of zinc (5-10 mg/d), OVERALL* | | | | | | | | | | | | |
| 3 (6 comparisons) | RCT | not serious | serious^12^ | serious^9^ | serious^5^ | none | 848 | 834 | - | -0.93 µg/L  (-2.46, 0.61) | ⊕⊝⊝⊝  VERY LOW^p^ | Critical |
| *In children receiving an ‘other’ form of zinc, < 5 mg/d* | | | | | | | | | | | | |
| 1 | NRCT | serious^7^ | NA | serious^13^ | serious^2^ | none | 21 | 26 | - | 6.65 µg/L  (-9.21, 22.51) | ⊕⊝⊝⊝  VERY LOW^q^ | Critical |
| *In children receiving an ‘other’ form of zinc, 10.1-20 mg/d* | | | | | | | | | | | | |
| 1 (2 comparisons) | RCT | not serious | not serious | serious^4^ | serious^2^ | none | 94 | 92 | - | -3.23 µg/L  (-8.40, 1.94) | ⊕⊕⊝⊝  LOW^c^ | Critical |
| *In children receiving an ‘other’ form of zinc, OVERALL* | | | | | | | | | | | | |
| 2 (3 comparisons) | RCT/ NRCT | serious^7^ | not serious | serious^8^ | serious^2^ | none | 115 | 118 | - | -2.28 µg/L  (-7.19, 2.63) | ⊕⊝⊝⊝  VERY LOW^f^ | Critical |
| **Ferritin concentration (µg/L): zinc versus placebo by dose** | | | | | | | | | | | | |
| *In children receiving < 5 mg/d* | | | | | | | | | | | | |
| 1 | NRCT | serious^7^ | NA | serious^13^ | serious^2^ | none | 21 | 26 | - | 6.65 µg/L  (-9.21, 22.51) | ⊕⊝⊝⊝  VERY LOW^q^ | Critical |
| *In children receiving 5-10 mg/d* | | | | | | | | | | | | |
| 10 (11 comparisons) | RCT | not serious | serious^12^ | serious^9^ | serious^5^ | none | 1084 | 1026 | - | -0.56 µg/L  (-2.35, 1.23) | ⊕⊝⊝⊝  VERY LOW^p^ | Critical |
| *In children receiving 10.1-20 mg/d* | | | | | | | | | | | | |
| 1 | RCT | not serious | NA | serious^4^ | serious^2^ | none | 47 | 46 | - | -3.20 µg/L  (-9.33, 2.93) | ⊕⊕⊝⊝  LOW^c^ | Critical |
| *In children receiving bolus (20 mg/wk or 140 mg/wk [70 mg twice weekly])* | | | | | | | | | | | | |
| 2 | RCT/ NRCT | serious^10^ | not serious | not serious | serious^2^ | none | 80 | 82 | - | 2.75 µg/L  (-3.30, 8.79) | ⊕⊕⊝⊝  LOW^i^ | Critical |
| *In children receiving zinc versus placebo, OVERALL* | | | | | | | | | | | | |
| 14 (15 comparisons) | RCT/ NRCT | not serious | serious^12^ | serious^9^ | serious^5^ | publication bias not detected | 1232 | 1180 | - | -0.41 µg/L  (-2.08, 1.25) | ⊕⊝⊝⊝  VERY LOW^r^ | Critical |
| **Ferritin concentration (µg/L): low versus high dose zinc** | | | | | | | | | | | | |
| *In children receiving low versus high dose zinc, OVERALL* | | | | | | | | | | | | |
| 2 | RCT | not serious | not serious | serious^6^ | serious^2^ | none | 91 | 83 | - | -8.10 µg/L  (-18.98, 2.78) | ⊕⊕⊝⊝  LOW^s^ | Critical |

**Explanations**

^1^Large difference in point estimates, limited overlap in confidence intervals, I^2^ ≥75%

^2^Wide confidence intervals including benefit and harm, small sample size

^3^Large variation in point estimates, I^2^ ≥50%

^4^Underlying malnutrition and anemia

^5^Wide confidence intervals including benefit and harm

^6^Underlying malnutrition, anemia and malaria

^7^High risk of bias in randomization process (selection bias) and deviations from intended interventions (performance bias)

^8^Underlying malnutrition and anemia, age range up to 6 years

^9^Underlying malnutrition, anemia and malaria, age range up to 60 months

^10^High risk of bias in randomization process (selection bias)

^11^Underlying anemia, age range up to 48 months

^12^Large variation in point estimates, I^2^ ≥75%

^13^Underlying anemia, age range up to 6 years

^a^Downgraded one level for inconsistency (large difference in point estimates, limited overlap in confidence intervals, I^2^ ≥75%) and one level for imprecision (wide confidence intervals including benefit and harm, small sample size)

^b^Downgraded one level for inconsistency (large variation in point estimates, I^2^ ≥50%) and one level for indirectness (underlying malnutrition and anemia)

^c^Downgraded one level for indirectness (underlying malnutrition and anemia) and one level for imprecision (wide confidence intervals including benefit and harm, small sample size)

^d^Downgraded one level for inconsistency (large difference in point estimates, limited overlap in confidence intervals, I^2^ ≥75%), one level for indirectness (underlying malnutrition, anemia and malaria) and one level for imprecision (wide confidence intervals including benefit and harm)

^e^Downgraded one level for imprecision (wide confidence intervals including benefit and harm, small sample size)

^f^Downgraded one level for study limitations (selection and performance bias), one level for indirectness (underlying malnutrition and anemia, age range up to 6 years) and one level for imprecision (wide confidence intervals including benefit and harm, small sample size)

^g^Downgraded one level for inconsistency (large difference in point estimates, limited overlap in confidence intervals, I^2^ ≥75%), one level for indirectness (underlying malnutrition, anemia and malaria, age range up to 60 months) and one level for imprecision (wide confidence intervals including benefit and harm)

^h^Downgraded one level for study limitations (selection bias), one level for indirectness (underlying malnutrition and anemia) and one level for imprecision (wide confidence intervals including benefit and harm, small sample size)

^i^Downgraded one level for study limitations (selection bias) and one level for imprecision (wide confidence intervals including benefit and harm, small sample size)

^j^Downgraded one level for study limitations (selection and performance bias), one level for inconsistency (large difference in point estimates, limited overlap in confidence intervals, I^2^ ≥75%), one level for indirectness (underlying malnutrition and anemia, age range up to 6 years) and one level for imprecision (wide confidence intervals including benefit and harm)

^k^Downgraded one level for indirectness (underlying malnutrition and anemia) and two levels for imprecision (very wide confidence intervals including benefit and harm, very small sample size)

^l^Downgraded one level for study limitations (selection bias), one level for indirectness (underlying anemia, age range up to 48 months) and one level for imprecision (wide confidence intervals including benefit and harm, small sample size)

^m^Downgraded one level for study limitations (selection bias), one level for indirectness (underlying and malnutrition anemia) and one level for imprecision (wide confidence intervals including benefit and harm, small sample size)

^n^Downgraded one level for inconsistency (large difference in point estimates, limited overlap in confidence intervals, I^2^ ≥75%), one level for indirectness (underlying malnutrition and anemia, age range up to 6 years) and one level for imprecision (wide confidence intervals including benefit and harm). Not downgraded for study limitations in this case as most data (>90%) were from studies at low or unclear risk of bias

^o^Downgraded one level for inconsistency (large difference in point estimates, limited overlap in confidence intervals, I^2^ ≥75%), one level for indirectness (underlying malnutrition, anemia and malaria) and one level for imprecision (wide confidence intervals including benefit and harm). Not downgraded for study limitations in this case as most data (>90%) were from studies at low or unclear risk of bias. The possibility of publication bias was not excluded but was not considered sufficient to downgrade the evidence

^p^Downgraded one level for inconsistency (large variation in point estimates, I^2^ ≥75%), one level for indirectness (underlying malnutrition, anemia and malaria, age range up to 60 months) and one level for imprecision (wide confidence intervals including benefit and harm)

^q^Downgraded one level for study limitations (selection and performance bias), one level for indirectness (underlying anemia, age range up to 6 years) and one level for imprecision (wide confidence intervals including benefit and harm, small sample size)

^r^Downgraded one level for inconsistency (large variation in point estimates, I^2^ ≥75%), one level for indirectness (underlying malnutrition, anemia and malaria, age range up to 60 months) and one level for imprecision (wide confidence intervals including benefit and harm). Not downgraded for study limitations in this case as most data (>90%) were from studies at low or unclear risk of bias

^s^Downgraded one level for indirectness (underlying malnutrition, anemia and malaria) and one level for imprecision (wide confidence intervals including benefit and harm, small sample size)

CI: confidence interval, NA: not applicable (as only 1 comparison), NRCT: non-randomized controlled trial, RCT: randomized controlled trial

**GRADE Working Group grades of evidence**
**High certainty:** We are very confident that the true effect lies close to that of the estimate of the effect
**Moderate certainty:** We are moderately confident in the effect estimate: The true effect is likely to be close to the estimate of the effect, but there is a possibility that it is substantially different
**Low certainty:** Our confidence in the effect estimate is limited: The true effect may be substantially different from the estimate of the effect
**Very low certainty:** We have very little confidence in the effect estimate: The true effect is likely to be substantially different from the estimate of effect

## GRADE Evidence table: Serum/plasma copper concentration (µg/dL)

| **Certainty assessment** | | | | | | | **Summary of findings** | | | | | **Importance** |
| --- | --- | --- | --- | --- | --- | --- | --- | --- | --- | --- | --- | --- |
|  |  |  |  |  |  |  | **No of patients** | | **Effect** | | **Certainty** |  |
| **No of studies** | **Design** | **Risk of bias** | **Inconsistency** | **Indirectness** | **Imprecision** | **Other considerations** | **Intervention** | **Control** | **Relative (95% CI)** | **Absolute (95% CI)** |  |  |
| **Serum/plasma copper concentration (µg/dL): by age and dose** | | | | | | | | | | | | |
| *In children aged 0-90 days (4 mg/d), OVERALL* | | | | | | | | | | | | |
| 1 | NRCT | serious^1^ | NA | not serious | serious^2^ | none | 34 | 34 | - | 11.90 µg/dL  (-4.60, 28.40) | ⊕⊕⊝⊝  LOW^a^ | Critical |
| *In children aged 91-180 days, 5 mg/d* | | | | | | | | | | | | |
| 1 | RCT | not serious | NA | serious^3^ | serious^2^ | none | 34 | 34 | - | -7.20 µg/dL  (-20.05, 5.65) | ⊕⊕⊝⊝  LOW^b^ | Critical |
| *In children aged 91-180 days, bolus 21 mg/wk* | | | | | | | | | | | | |
| 1 | RCT | not serious | NA | not serious | serious^2^ | none | 329 | 309 | - | 0.00 µg/dL  (-3.50, 3.50) | ⊕⊕⊕⊝  MODERATE^c^ | Critical |
| *In children aged 91-180 days, OVERALL* | | | | | | | | | | | | |
| 2 | RCT | not serious | not serious | serious^3^ | serious^2^ | none | 363 | 343 | - | -0.84 µg/dL  (-5.36, 3.68) | ⊕⊕⊝⊝  LOW^b^ | Critical |
| *In children aged 6–12 months, < 5 mg/d* | | | | | | | | | | | | |
| 1 (2 comparisons) | RCT | not serious | not serious | serious^3^ | serious^4^ | none | 137 | 76 | - | -10.25 µg/dL  (-20.18, -0.33) | ⊕⊕⊝⊝  LOW^d^ | Critical |
| *In children aged 6–12 months, 5-10 mg/d* | | | | | | | | | | | | |
| 3 (4 comparisons) | RCT | not serious | not serious | serious^5^ | serious^6^ | none | 406 | 412 | - | -1.49 µg/dL  (-5.60, 2.63) | ⊕⊕⊝⊝  LOW^e^ | Critical |
| *In children aged 6–12 months, bolus 20 mg/wk* | | | | | | | | | | | | |
| 1 (2 comparisons) | RCT | not serious | not serious | not serious | serious^2^ | none | 83 | 79 | - | -4.23 µg/dL  (-10.38, 1.93) | ⊕⊕⊕⊝  MODERATE^c^ | Critical |
| *In children aged 6–12 months, OVERALL* | | | | | | | | | | | | |
| 5 (8 comparisons) | RCT | not serious | not serious | serious^7^ | serious^6^ | none | 626 | 567 | - | -3.17 µg/dL  (-6.41, 0.06) | ⊕⊕⊝⊝  LOW^f^ | Critical |
| *In children aged > 12 months, < 5 mg/d* | | | | | | | | | | | | |
| 1 | NRCT | serious^1^ | NA | serious^8^ | serious^2^ | none | 30 | 28 | - | -7.52 µg/dL  (-21.69, 6.65) | ⊕⊝⊝⊝  VERY LOW^g^ | Critical |
| *In children aged > 12 months, 5-10 mg/d* | | | | | | | | | | | | |
| 1 | RCT | not serious | NA | serious^9^ | serious^2^ | none | 53 | 49 | - | 1.40 µg/dL  (-7.82, 10.62) | ⊕⊕⊝⊝  LOW^h^ | Critical |
| *In children aged > 12 months, 10.1-20 mg/d* | | | | | | | | | | | | |
| 2 | RCT | not serious | not serious | serious^5^ | not serious | none | 1253 | 1265 | - | -15.61 µg/dL  (-18.17,  -13.06) | ⊕⊕⊕⊝  MODERATE^i^ | Critical |
| *In children aged > 12 months, >20 mg/d* | | | | | | | | | | | | |
| 1 (2 comparisons) | RCT | serious^10^ | not serious | serious^5^ | serious^6^ | none | 877 | 450 | - | 0.02 µg/dL  (-0.41, 0.45) | ⊕⊝⊝⊝  VERY LOW^j^ | Critical |
| *In children aged > 12 months, OVERALL* | | | | | | | | | | | | |
| 5 (6 comparisons | RCT | serious^11^ | serious^12^ | serious^13^ | not serious | none | 2223 | 1792 | - | -5.25 µg/dL  (-8.11, -2.39) | ⊕⊝⊝⊝  VERY LOW^k^ | Critical |
| **Serum/plasma copper concentration (µg/dL): by treatment duration and dose** | | | | | | | | | | | | |
| *In children receiving interventions for 0–3 months, 10.1-20 mg/d* | | | | | | | | | | | | |
| 1 | RCT | not serious | NA | serious^5^ | serious^4^ | none | 43 | 44 | - | -16.50 µg/dL  (-24.09, -8.91) | ⊕⊕⊝⊝  LOW^l^ | Critical |
| *In children receiving interventions for 0–3 months, > 20 mg/d* | | | | | | | | | | | | |
| 1 (2 comparisons) | RCT | serious^10^ | not serious | serious^5^ | serious^6^ | none | 887 | 450 | - | 0.02 µg/dL  (-0.41, 0.45) | ⊕⊝⊝⊝  VERY LOW^j^ | Critical |
| *In children receiving interventions for 0–3 months, OVERALL* | | | | | | | | | | | | |
| 2 (3 comparisons) | RCT | serious^10^ | serious^12^ | serious^5^ | serious^6^ | none | 930 | 494 | - | -0.50 µg/dL  (-1.90, 0.90) | ⊕⊝⊝⊝  VERY LOW^m^ | Critical |
| *In children receiving interventions for > 3 months, < 5 mg/d* | | | | | | | | | | | | |
| 3 (4 comparisons) | RCT/ NRCT | serious^1^ | serious^14^ | serious^15^ | serious^2^ | none | 201 | 138 | - | -4.62 µg/dL  (-15.01, 5.78) | ⊕⊝⊝⊝  VERY LOW^n^ | Critical |
| *In children receiving interventions for > 3 months, 5–10 mg/d* | | | | | | | | | | | | |
| 5 (6 comparisons) | RCT | not serious | not serious | serious^5^ | serious^6^ | none | 493 | 495 | - | -1.50 µg/dL  (-5.10, 2.11) | ⊕⊕⊝⊝  LOW^e^ | Critical |
| *In children receiving interventions for > 3 months, 10.1-20 mg/d* | | | | | | | | | | | | |
| 1 | RCT | not serious | NA | serious^9^ | not serious | none | 1210 | 1221 | - | -15.50 µg/dL  (-18.21,  -12.79) | ⊕⊕⊕⊝  MODERATE^o^ | Critical |
| *In children receiving interventions for > 3 months, bolus 20 mg/wk or 21 mg/wk* | | | | | | | | | | | | |
| 2 (3 comparisons) | RCT | not serious | not serious | not serious | serious^6^ | none | 412 | 388 | - | -1.03 µg/dL  (-4.08, 2.01) | ⊕⊕⊕⊝  MODERATE^p^ | Critical |
| *In children receiving interventions for > 3 months, OVERALL* | | | | | | | | | | | | |
| 11 (14 comparisons) | RCT/ NRCT | not serious | serious^12^ | serious^15^ | serious^6^ | publication bias not detected | 2316 | 2242 | - | -4.00 µg/dL  (-8.82, 0.82) | ⊕⊝⊝⊝  VERY LOW^q^ | Critical |
| **Serum/plasma copper concentration (µg/dL): by zinc form and dose** | | | | | | | | | | | | |
| *In children receiving zinc as zinc gluconate, 5-10 mg/d* | | | | | | | | | | | | |
| 1 | RCT | not serious | NA | serious^5^ | serious^2^ | none | 58 | 52 | - | -5.20 µg/dL  (-20.16, 9.76) | ⊕⊕⊝⊝  LOW^r^ | Critical |
| *In children receiving zinc as zinc gluconate, 10.1-20 mg/d* | | | | | | | | | | | | |
| 1 | RCT | not serious | NA | serious^9^ | not serious | none | 1210 | 1221 | - | -15.50 µg/dL  (-18.21,  -12.79) | ⊕⊕⊕⊝  MODERATE^o^ | Critical |
| *In children receiving zinc as zinc gluconate, OVERALL* | | | | | | | | | | | | |
| 2 | RCT | not serious | not serious | serious^5^ | not serious | none | 1268 | 1273 | - | -13.08 µg/dL  (-21.64,  -4.53) | ⊕⊕⊕⊝  MODERATE^i^ | Critical |
| *In children receiving zinc as zinc sulphate, < 5 mg/d* | | | | | | | | | | | | |
| 1 (2 comparisons) | RCT | not serious | not serious | serious^3^ | serious^4^ | none | 137 | 76 | - | -10.25 µg/dL  (-20.18, -0.33) | ⊕⊕⊝⊝  LOW^d^ | Critical |
| *In children receiving zinc as zinc sulphate, 5-10 mg/d* | | | | | | | | | | | | |
| 5 (6 comparisons) | RCT/ NRCT | not serious | not serious | serious^3^ | serious^6^ | none | 469 | 477 | - | -0.63 µg/dL  (-4.26, 2.99) | ⊕⊕⊝⊝  LOW^t^ | Critical |
| *In children receiving zinc as zinc sulphate, 10.1-20 mg/d* | | | | | | | | | | | | |
| 1 | RCT | not serious | NA | serious^5^ | serious^4^ | none | 43 | 44 | - | -16.50 µg/dL  (-24.09, -8.91) | ⊕⊕⊝⊝  LOW^l^ | Critical |
| *In children receiving zinc as zinc sulphate, OVERALL* | | | | | | | | | | | | |
| 7 (9 comparisons) | RCT | not serious | serious^14^ | serious^7^ | serious^6^ | none | 649 | 597 | - | -4.09 µg/dL  (-9.31, 1.12) | ⊕⊝⊝⊝  VERY LOW^u^ | Critical |
| *In children receiving zinc as zinc acetate (bolus 20 and 21 mg/wk), OVERALL* | | | | | | | | | | | | |
| 2 (3 comparisons) | RCT | not serious | not serious | not serious | serious^6^ | none | 412 | 388 | - | -1.03 µg/dL  (-4.08, 2.01) | ⊕⊕⊕⊝  MODERATE^p^ | Critical |
| *In children receiving an ‘other’ form of zinc (zinc oxide), OVERALL* | | | | | | | | | | | | |
| 1 | NRCT | serious^1^ | NA | serious^8^ | serious^2^ | none | 30 | 28 | - | -7.52 µg/dL  (-21.69, 6.65) | ⊕⊝⊝⊝  VERY LOW^g^ | Critical |
| **Serum/plasma copper concentration (µg/dL): zinc versus placebo by dose** | | | | | | | | | | | | |
| *In children receiving < 5 mg/d* | | | | | | | | | | | | |
| 1 | NRCT | serious^1^ | NA | serious^8^ | serious^2^ | none | 30 | 28 | - | -7.52 µg/dL  (-21.69, 6.65) | ⊕⊝⊝⊝  VERY LOW^g^ | Critical |
| *In children receiving 5-10 mg/d* | | | | | | | | | | | | |
| 3 | RCT | not serious | not serious | serious^3^ | serious^2^ | none | 221 | 226 | - | -1.41 µg/dL  (-6.76, 3.93) | ⊕⊕⊝⊝  LOW^b^ | Critical |
| *In children receiving 10.1–20 mg/d* | | | | | | | | | | | | |
| 2 | RCT | not serious | not serious | serious^5^ | not serious | none | 1253 | 1265 | - | -15.61 µg/dL  (-18.17,  -13.06) | ⊕⊕⊕⊝  MODERATE^i^ | Critical |
| *In children receiving > 20 mg/d* | | | | | | | | | | | | |
| 1 (2 comparisons) | RCT | serious^1^ | not serious | serious^7^ | serious^6^ | none | 877 | 450 | - | 0.02 µg/dL  (-0.41, 0.45) | ⊕⊝⊝⊝  VERY LOW^v^ | Critical |
| *In children receiving bolus 20 and 21 mg/wk* | | | | | | | | | | | | |
| 2 | RCT | not serious | not serious | not serious | serious^2^ | none | 371 | 347 | - | -0.21 µg/dL  (-3.52, 3.09) | ⊕⊕⊕⊝  MODERATE^c^ | Critical |
| *In children receiving zinc versus placebo, OVERALL* | | | | | | | | | | | | |
| 9 (10 comparisons) | RCT/ NRCT | serious^11^ | serious^12^ | serious^16^ | not serious | publication bias not detected | 2762 | 2316 | - | -4.11 µg/dL  (-6.48, -1.74) | ⊕⊝⊝⊝  VERY LOW^w^ | Critical |

**Explanations**

**^1^**High risk of bias in randomization process (selection bias)

^2^Wide confidence intervals including benefit and harm, small sample size

^3^Underlying malnutrition and anemia

^4^Small sample size

^5^Acute diarrhoea/gastroenteritis and underlying malnutrition

^6^Wide confidence intervals including benefit and harm

^7^Acute diarrhoea/gastroenteritis and underlying malnutrition and anemia

^8^Age range up to 90 months

^9^Underlying malnutrition

^10^High risk of information bias (1 zinc arm in Strand 2002 open)

^11^High risk of bias in randomization process (selection bias), high risk of information bias (1 zinc arm in Strand 2002 open)

^12^Large difference in point estimates, limited overlap in confidence intervals, I^2^≥75%

^13^Acute diarrhoea/gastroenteritis, underlying malnutrition, age range up to 90 months

^14^Large difference in point estimates, I^2^≥50%

^15^Underlying malnutrition and anemia, age range up to 90 months

^16^Acute diarrhoea/gastroenteritis, underlying anemia and malnutrition, age range up to 90 months

^a^Downgraded one level for study limitations (selection bias) and one level for imprecision (wide confidence intervals including benefit and harm, small sample size)

^b^Downgraded one level for indirectness (underlying malnutrition and anemia) and one level for imprecision (wide confidence intervals including benefit and harm, small sample size)

^c^Downgraded one level for imprecision (wide confidence intervals including benefit and harm, small sample size)

^d^Downgraded one level for indirectness (underlying malnutrition and anemia) and one level for imprecision (wide confidence intervals, small sample size)

^e^Downgraded one level for indirectness (acute diarrhoea/gastroenteritis and underlying malnutrition) and one level for imprecision (wide confidence intervals including benefit and harm)

^f^Downgraded one level for indirectness (acute diarrhoea/gastroenteritis and underlying malnutrition and anemia) and one level for imprecision (wide confidence intervals including benefit and harm)

^g^Downgraded one level for study limitations (selection bias), on level for indirectness (age range up to 90 months) and one level for imprecision (wide confidence intervals including benefit and harm, small sample size)

^h^Downgraded one level for indirectness (underlying malnutrition) and one level for imprecision (wide confidence intervals including benefit and harm, small sample size)

^i^Downgraded one level for indirectness (acute diarrhoea/gastroenteritis and underlying malnutrition)

^j^Downgraded one level for study limitations (information bias), one level for indirectness (acute diarrhoea/gastroenteritis and underlying malnutrition) and one level for imprecision (wide confidence intervals including benefit and harm)

^k^Downgraded one level for study limitations (selection and information bias), one level for inconsistency (large difference in point estimates, limited overlap in confidence intervals, I^2^≥75%) and one level for indirectness (acute diarrhoea/gastroenteritis, underlying malnutrition, age range up to 90 months)

^l^Downgraded one level for indirectness (acute diarrhoea/gastroenteritis and underlying malnutrition) and one level for imprecision (small sample size)

^m^Downgraded one level for study limitations (information bias), one level for inconsistency (large difference in point estimates, limited overlap in confidence intervals, I^2^≥75%), one level for indirectness (acute diarrhoea/gastroenteritis and underlying malnutrition) and one level for imprecision (wide confidence intervals including benefit and harm)

^n^Downgraded one level for study limitations (selection bias), one level for inconsistency (large difference in point estimates, I^2^≥50%), one level for indirectness (underlying malnutrition and anemia, age range up to 90 months) and one level for imprecision (wide confidence intervals including benefit and harm, small sample size)

^o^Downgraded one level for indirectness (underlying malnutrition)

^p^Downgraded one level for imprecision (wide confidence intervals including benefit and harm)

^q^Downgraded one level for inconsistency (large difference in point estimates, limited overlap in confidence intervals, I^2^≥75%), one level for indirectness (underlying malnutrition and anemia, age range up to 90 months) and one level for imprecision (wide confidence intervals including benefit and harm). Not downgraded for study limitations in this case as most data (>90%) were from studies at low or unclear risk of bias

^r^Downgraded one level for indirectness (acute diarrhoea/gastroenteritis and underlying malnutrition) and one level for imprecision (wide confidence intervals including benefit and harm, small sample size)

^s^Downgraded one level for indirectness (acute diarrhoea/gastroenteritis and underlying malnutrition) and one level for imprecision (wide confidence intervals)

^t^Downgraded one level for indirectness (underlying malnutrition and anemia) and one level for imprecision (wide confidence intervals including benefit and harm). Not downgraded for study limitations in this case as most data (>90%) were from studies at low or unclear risk of bias

^u^Downgraded one level for inconsistency (large difference in point estimates, I^2^≥50%), one level for indirectness (acute diarrhoea/gastroenteritis and underlying malnutrition and anemia) and one level for imprecision (wide confidence intervals including benefit and harm). Not downgraded for study limitations in this case as most data (>90%) were from studies at low or unclear risk of bias

^v^Downgraded one level for study limitations (selection bias), one level for indirectness (acute diarrhoea/gastroenteritis and underlying malnutrition and anemia) and one level for imprecision (wide confidence intervals including benefit and harm)

^w^Downgraded one level for study limitations (selection and information bias), one level for inconsistency (large difference in point estimates, limited overlap in confidence intervals, I^2^≥75%) and one level for indirectness (acute diarrhoea/gastroenteritis, underlying anemia and malnutrition, age range up to 90 months)

CI: confidence interval, NA: not applicable (as only 1 comparison), NRCT: non-randomized controlled trial, RCT: randomized controlled trial

**GRADE Working Group grades of evidence**
**High certainty:** We are very confident that the true effect lies close to that of the estimate of the effect
**Moderate certainty:** We are moderately confident in the effect estimate: The true effect is likely to be close to the estimate of the effect, but there is a possibility that it is substantially different
**Low certainty:** Our confidence in the effect estimate is limited: The true effect may be substantially different from the estimate of the effect
**Very low certainty:** We have very little confidence in the effect estimate: The true effect is likely to be substantially different from the estimate of effect

## GRADE Evidence table: Iron deficiency

| **Certainty assessment** | | | | | | | **Summary of findings** | | | | | **Importance** |
| --- | --- | --- | --- | --- | --- | --- | --- | --- | --- | --- | --- | --- |
|  |  |  |  |  |  |  | **No of patients** | | **Effect** | | **Certainty** |  |
| **No of studies** | **Design** | **Risk of bias** | **Inconsistency** | **Indirectness** | **Imprecision** | **Other considerations** | **Intervention** | **Control** | **Relative**  **(95% CI)** | **Absolute** |  |  |
| **Iron deficiency: by age** | | | | | | | | | | | | |
| *In children aged 0-90 days (5-10 mg/d)* | | | | | | | | | | | | |
| 1 (2 comparisons) | RCT | not serious | not serious | not serious | serious^1^ | none | 34/295 | 20/298 | OR 1.81  (1.02, 3.24) | 12 per 100 in Zn vs 7 per 100 in control | ⊕⊕⊕⊝  MODERATE^a^ | Critical |
| *In children aged 91-180 days (10 mg/d)* | | | | | | | | | | | | |
| 2 (4 comparisons) | RCT | not serious | not serious | serious^2^ | serious^3^ | none | 69/482 | 65/502 | OR 1.27  (0.69, 2.36) | 14 per 100 in Zn vs 13 per 100 in control | ⊕⊕⊝⊝  LOW^b^ | Critical |
| *In children aged 6–12 months (3-10 mg/d)* | | | | | | | | | | | | |
| 5 (8 comparisons) | RCT | not serious | not serious | serious^4^ | serious^5^ | none | 351/1030 | 319/945 | OR 0.90 (0.68, 1.19) | 34 per 100 in Zn vs 34 per 100 in control | ⊕⊕⊝⊝  LOW^c^ | Critical |
| *In children aged >12 months (10-20 mg/d)* | | | | | | | | | | | | |
| 2 (4 comparisons) | RCT | not serious | not serious | serious^6^ | serious^3^ | none | 28/391 | 32/393 | OR 0.86  (0.49, 1.49) | 7 per 100 in Zn vs 8 per 100 in control | ⊕⊕⊝⊝  LOW^d^ | Critical |
| *In children aged 0–>12 months (3-20 mg/d), OVERALL* | | | | | | | | | | | | |
| 10 (18 comparisons) | RCT | not serious | not serious | serious^6^ | serious^5^ | publication bias not detected | 482/2198 | 436/2138 | OR 1.01 (0.83, 1.23) | 22 per 100 in Zn vs 20 per 100 in control | ⊕⊕⊝⊝  LOW^e^ | Critical |
| **Iron deficiency: by treatment duration** | | | | | | | | | | | | |
| *In children receiving interventions for < 3 months (10 mg/d), OVERALL* | | | | | | | | | | | | |
| 1 | RCT | not serious | NA | serious^4^ | serious^3^ | none | 17/110 | 22/92 | OR 0.58  (0.29, 1.18) | 15 per 100 in Zn vs 24 per 100 in control | ⊕⊕⊝⊝  LOW^c^ | Critical |
| *In children receiving interventions for > 3 months (3-20 mg/d), OVERALL* | | | | | | | | | | | | |
| 9 (17 comparisons) | RCT | not serious | not serious | serious^6^ | serious^5^ | publication bias not detected^7^ | 465/2088 | 414/2038 | OR 1.02 (0.80, 1.30) | 22 per 100 in Zn vs 20 per 100 in control | ⊕⊕⊝⊝  LOW^e^ | Critical |
| **Iron deficiency: by zinc form** | | | | | | | | | | | | |
| *In children receiving zinc as zinc gluconate (10 mg/d), OVERALL* | | | | | | | | | | | | |
| 1 | RCT | not serious | NA | serious^4^ | serious^3^ | none | 17/110 | 22/92 | OR 0.58  (0.29, 1.18) | 15 per 100 in Zn vs 24 per 100 in control | ⊕⊕⊝⊝  LOW^c^ | Critical |
| *In children receiving zinc as zinc sulphate (3-10 mg/d), OVERALL* | | | | | | | | | | | | |
| 5 (10 comparisons) | RCT | not serious | not serious | serious^2^ | serious^3^ | publication bias suspected^8^ | 249/1184 | 202/1155 | OR 1.14  (0.82, 1.60) | 21 per 100 in Zn vs 17 per 100 in control | ⊕⊕⊝⊝  LOW^b^ | Critical |
| *In children receiving an unstated form of zinc (5-10 mg/d), OVERALL* | | | | | | | | | | | | |
| 3 (5 comparisons) | RCT | not serious | not serious | serious^6^ | serious^3^ | none | 205/810 | 201/799 | OR 1.04 (0.80, 1.34) | 25 per 100 in Zn vs 25 per 100 in control | ⊕⊕⊝⊝  LOW^d^ | Critical |
| *In children receiving an ‘other’ form of zinc (zinc methionine 12 mg/d), OVERALL* | | | | | | | | | | | | |
| 1 (2 comparisons) | RCT | not serious | NA | serious^2^ | serious^3^ | none | 11/94 | 11/92 | OR 0.97 (0.37, 2.53) | 12 per 100 in Zn vs 12 per 100 in control | ⊕⊝⊝⊝  VERY LOW^f^ | Critical |
| **Iron deficiency: zinc versus placebo** | | | | | | | | | | | | |
| *In children receiving zinc versus placebo (5-20 mg/d), OVERALL* | | | | | | | | | | | | |
| 7 | RCT | not serious | not serious | serious^6^ | serious^3^ | none | 189/747 | 196/775 | OR 1.04 (0.79, 1.37) | 25 per 100 in Zn vs 25 per 100 in control | ⊕⊕⊝⊝  LOW^d^ | Critical |

**Explanations**

^1^Wide confidence intervals, small number of events

^2^Underlying malnutrition and anemia

^3^Wide confidence intervals including benefit and harm, small number of events

^4^Underlying malnutrition, anemia and malaria

^5^Wide confidence intervals including benefit and harm

^6^Underlying malnutrition, anemia and malaria, age range up to 60 months

^7^Funnel plot includes all treatment durations

^8^Funnel plot includes all zinc forms

^a^Downgraded one level for imprecision (wide confidence intervals, small number of events)

^b^Downgraded one level for indirectness (underlying malnutrition and anemia) and one level for imprecision (wide confidence intervals including benefit and harm, small number of events). The possibility of publication bias was not excluded for zinc sulphate, but was not considered sufficient to downgrade the evidence

^c^Downgraded one level for indirectness (underlying malnutrition, anemia and malaria) and one level for imprecision (wide confidence intervals including benefit and harm, small number of events)

^d^Downgraded one level for indirectness (underlying malnutrition, anemia and malaria, age range up to 60 months) and one level for imprecision (wide confidence intervals including benefit and harm, small number of events)

^e^Downgraded one level for indirectness (underlying malnutrition, anemia and malaria, age range up to 60 months) and one level for imprecision (wide confidence intervals including benefit and harm)

^f^Downgraded one level for indirectness (underlying malnutrition and anemia) and two levels for imprecision (very wide confidence intervals including benefit and harm, very small number of events)

CI: confidence interval, NA: not applicable (as only 1 comparison), RCT: randomized controlled trial, OR: odds ratio

**GRADE Working Group grades of evidence**
**High certainty:** We are very confident that the true effect lies close to that of the estimate of the effect
**Moderate certainty:** We are moderately confident in the effect estimate: The true effect is likely to be close to the estimate of the effect, but there is a possibility that it is substantially different
**Low certainty:** Our confidence in the effect estimate is limited: The true effect may be substantially different from the estimate of the effect
**Very low certainty:** We have very little confidence in the effect estimate: The true effect is likely to be substantially different from the estimate of effect

## GRADE Evidence table: Iron deficiency anemia

| **Certainty assessment** | | | | | | | **Summary of findings** | | | | | **Importance** |
| --- | --- | --- | --- | --- | --- | --- | --- | --- | --- | --- | --- | --- |
|  |  |  |  |  |  |  | **No of patients** | | **Effect** | | **Certainty** |  |
| **No of studies** | **Design** | **Risk of bias** | **Inconsistency** | **Indirectness** | **Imprecision** | **Other considerations** | **Intervention** | **Control** | **Relative**  **(95% CI)** | **Absolute** |  |  |
| **Iron deficiency anemia: by age** | | | | | | | | | | | | |
| *In children aged 91-180 days (10 mg/d)* | | | | | | | | | | | | |
| 3 (6 comparisons) | RCT | not serious | serious^1^ | serious^2^ | serious^3^ | none | 74/653 | 74/679 | OR 1.23  (0.61, 2.47) | 11 per 100 in Zn vs 11 per 100 in control | ⊕⊝⊝⊝  VERY LOW^a^ | Critical |
| *In children aged 6–12 months (5-10 mg/d)* | | | | | | | | | | | | |
| 3 (7 comparisons) | RCT | not serious | not serious | serious^4^ | serious^3^ | none | 100/518 | 80/470 | OR 0.83 (0.57, 1.22) | 19 per 100 in Zn vs 17 per 100 in control | ⊕⊕⊝⊝  LOW^b^ | Critical |
| *In children aged 91 days–12 months (5-10 mg/d), OVERALL* | | | | | | | | | | | | |
| 6 (13 comparisons) | RCT | not serious | not serious | serious^4^ | serious^3^ | publication bias not detected | 174/1171 | 154/1149 | OR 1.01 (0.73, 1.39) | 15 per 100 in Zn vs 13 per 100 in control | ⊕⊕⊝⊝  LOW^b^ | Critical |
| **Iron deficiency anemia: by treatment duration** | | | | | | | | | | | | |
| *In children receiving interventions for > 3 months (3-10 mg/d), OVERALL* | | | | | | | | | | | | |
| 6 (13 comparisons) | RCT | not serious | not serious | serious^4^ | serious^3^ | publication bias not detected | 174/1171 | 154/1149 | OR 1.01 (0.73, 1.39) | 15 per 100 in Zn vs 13 per 100 in control | ⊕⊕⊝⊝  LOW^b^ | Critical |
| **Iron deficiency anemia: by zinc form** | | | | | | | | | | | | |
| *In children receiving zinc as zinc sulphate (3-10 mg/d), OVERALL* | | | | | | | | | | | | |
| 5 (10 comparisons) | RCT | not serious | not serious | serious^2^ | serious^3^ | publication bias not detected^5^ | 167/1060 | 149/1034 | OR 0.99  (0.68, 1.44) | 16 per 100 in Zn vs 14 per 100 in control | ⊕⊕⊝⊝  LOW^c^ | Critical |
| *In children receiving an unstated form of zinc (5-10 mg/d), OVERALL* | | | | | | | | | | | | |
| 1 (3 comparisons) | RCT | not serious | not serious | serious^4^ | serious^3^ | none | 7/111 | 5/115 | OR 1.46 (0.42, 5.06) | 6 per 100 in Zn vs 4 per 100 in control | ⊕⊝⊝⊝  VERY LOW^d^ | Critical |
| **Iron deficiency anemia: zinc versus placebo** | | | | | | | | | | | | |
| *In children receiving zinc versus placebo (5-10 mg/d), OVERALL* | | | | | | | | | | | | |
| 6 (6 comparisons) | RCT | not serious | not serious | serious^4^ | serious^3^ | none | 94/609 | 93/552 | OR 0.98 (0.67, 1.45) | 15 per 100 in Zn vs 17 per 100 in control | ⊕⊕⊝⊝  LOW^b^ | Critical |
| **Iron deficiency anemia: low versus high dose zinc** | | | | | | | | | | | | |
| *In children receiving high versus low dose zinc (10 mg vs 5 mg/d), OVERALL* | | | | | | | | | | | | |
| 1 | RCT | not serious | NA | serious^4^ | serious^3^ | none | 5/76 | 1/71 | OR 4.93 (0.56, 43.27) | 7 per 100 in Zn vs 1 per 100 in control | ⊕⊝⊝⊝  VERY LOW^d^ | Critical |

**Explanations**

^1^Large difference in point estimates, limited overlap in confidence intervals, I^2^≥50%

^2^Underlying malnutrition and anemia

^3^Wide confidence intervals including benefit and harm, small number of events

^4^Underlying anemia, malaria and malnutrition

^5^Funnel plot also includes unstated zinc form

^a^Downgraded one level for inconsistency (large difference in point estimates, limited overlap in confidence intervals, I^2^≥50%), one level for indirectness (underlying malnutrition and anemia), and one level for imprecision (wide confidence intervals including benefit and harm, small number of events)

^b^Downgraded one level for indirectness (underlying anemia, malaria and malnutrition) and one level for imprecision (wide confidence intervals including benefit and harm, small number of events)

^c^Downgraded one level for indirectness (underlying malnutrition and anemia), and one level for imprecision (wide confidence intervals including benefit and harm, small number of events)

^d^Downgraded one level for indirectness (underlying anemia, malaria and malnutrition) and two levels for imprecision (very wide confidence intervals including benefit and harm, very small number of events)

CI: confidence interval, NA: not applicable (as only 1 comparison), RCT: randomized controlled trial, OR: odds ratio

**GRADE Working Group grades of evidence**
**High certainty:** We are very confident that the true effect lies close to that of the estimate of the effect
**Moderate certainty:** We are moderately confident in the effect estimate: The true effect is likely to be close to the estimate of the effect, but there is a possibility that it is substantially different
**Low certainty:** Our confidence in the effect estimate is limited: The true effect may be substantially different from the estimate of the effect
**Very low certainty:** We have very little confidence in the effect estimate: The true effect is likely to be substantially different from the estimate of effect

## GRADE Evidence table: Serum/soluble transferrin receptor concentration (mg/L)

| **Certainty assessment** | | | | | | | **Summary of findings** | | | | | **Importance** |
| --- | --- | --- | --- | --- | --- | --- | --- | --- | --- | --- | --- | --- |
|  |  |  |  |  |  |  | **No of patients** | | **Effect** | | **Certainty** |  |
| **No of studies** | **Design** | **Risk of bias** | **Inconsistency** | **Indirectness** | **Imprecision** | **Other considerations** | **Intervention** | **Control** | **Relative (95% CI)** | **Absolute (95% CI)** |  |  |
| **Serum/soluble transferrin receptor concentration (mg/L): by age** | | | | | | | | | | | | |
| *In children aged 0-90 days* | | | | | | | | | | | | |
| 1 (2 comparisons) | RCT | not serious | serious^1^ | not serious | serious^2^ | none | 295 | 295 | - | 0.05 mg/L  (-0.05, 0.15) | ⊕⊕⊝⊝  LOW^a^ | Critical |
| *In children aged 6–12 months* | | | | | | | | | | | | |
| 4 (10 comparisons) | RCT | not serious | not serious | serious^3^ | not serious | none | 733 | 662 | - | 0.63 mg/L  (0.42, 0.85) | ⊕⊕⊕⊝  MODERATE^b^ | Critical |
| *In children aged 0-12 months, OVERALL* | | | | | | | | | | | | |
| 5 (12 comparisons) | RCT | not serious | serious^4^ | serious^3^ | not serious | publication bias not detected | 1028 | 957 | - | 0.19 mg/L  (0.08, 0.29) | ⊕⊕⊝⊝  LOW^c^ | Critical |
| **Serum/soluble transferrin receptor concentration (mg/L): by treatment duration** | | | | | | | | | | | | |
| *In children receiving interventions for > 3 months, OVERALL* | | | | | | | | | | | | |
| 5 (12 comparisons) | RCT | not serious | serious^4^ | serious^3^ | not serious | publication bias not detected | 1028 | 957 | - | 0.19 mg/L  (0.08, 0.29) | ⊕⊕⊝⊝  LOW^c^ | Critical |
| **Serum/soluble transferrin receptor concentration (mg/L): by zinc form** | | | | | | | | | | | | |
| *In children receiving zinc as zinc sulphate (5-10 mg/d), OVERALL* | | | | | | | | | | | | |
| 2 (4 comparisons) | RCT | not serious | serious^4^ | not serious | not serious | none | 565 | 574 | - | 0.18 mg/L  (0.07, 0.29) | ⊕⊕⊕⊝  MODERATE^d^ | Critical |
| *In children receiving zinc as zinc acetate (20 mg/d), OVERALL* | | | | | | | | | | | | |
| 1 (2 comparisons | RCT | not serious | serious^5^ | not serious | serious^2^ | none | 83 | 80 | - | 0.44 mg/L  (-1.08, 1.96) | ⊕⊕⊝⊝  LOW^e^ | Critical |
| *In children receiving an unstated form of zinc (5–10 mg/d), OVERALL* | | | | | | | | | | | | |
| 2 (6 comparisons) | RCT | not serious | not serious | serious^3^ | serious^2^ | publication bias not detected^7^ | 380 | 303 | - | 0.26 mg/L  (-0.13, 0.65) | ⊕⊕⊝⊝  LOW^f^ | Critical |
| **Serum/soluble transferrin receptor concentration (mg/L): zinc versus placebo** | | | | | | | | | | | | |
| *In children receiving zinc versus placebo (5-20 mg/d), OVERALL* | | | | | | | | | | | | |
| 5 (6 comparisons) | RCT | not serious | serious^5^ | serious^3^ | serious^6^ | none | 566 | 505 | - | 0.43 mg/L  (-0.06, 0.93) | ⊕⊝⊝⊝  VERY LOW^g^ | Critical |
| **Serum/soluble transferrin receptor concentration (mg/L): low versus high dose zinc** | | | | | | | | | | | | |
| *In children receiving low versus high dose zinc (5 vs 10 mg/d), OVERALL* | | | | | | | | | | | | |
| 1 | RCT | not serious | NA | serious^8^ | serious^2^ | none | 79 | 75 | - | -0.10 mg/L  (-1.19, 0.99) | ⊕⊕⊝⊝  LOW^h^ | Critical |

**Explanations**

^1^I^2^≥75%

^2^Wide confidence intervals including benefit and harm, small sample size

^3^Underlying malnutrition, anemia and malaria

^4^Large variation in point estimates, limited overlap in confidence intervals, I^2^≥75%

^5^Large variation in point estimates, limited overlap in confidence intervals, I^2^≥50%

^6^Wide confidence intervals including benefit and harm

^7^Funnel plot includes all zinc forms

^8^Underlying anemia and malaria

^a^Downgraded one level for inconsistency (I^2^≥75%) and one level for imprecision (wide confidence intervals including benefit and harm, small sample size)

^b^Downgraded one level for indirectness (underlying malnutrition, anemia and malaria)

^c^Downdgraded one level for inconsistency (large variation in point estimates, limited overlap in confidence intervals, I^2^≥75%) and one level for indirectness (underlying malnutrition, anemia and malaria)

^d^Downgraded one level for inconsistency (large variation in point estimates, limited overlap in confidence intervals, I^2^≥75%)

^e^Downgraded one level for inconsistency (large variation in point estimates, limited overlap in confidence intervals, I^2^≥50%) and one level for imprecision (wide confidence intervals including benefit and harm, small sample size)

^f^Downgraded one level for indirectness (underlying malnutrition, anemia and malaria) and one level for imprecision (wide confidence intervals including benefit and harm, small sample size)

^g^Downgraded one level for inconsistency (large variation in point estimates, limited overlap in confidence intervals, I^2^≥50%), one level for indirectness (underlying malnutrition, anemia and malaria) and one level for imprecision (wide confidence intervals including benefit and harm)

^h^Downgraded one level for indirectness (underlying anemia and malaria) and one level for imprecision (wide confidence intervals including benefit and harm, small sample size)

CI: confidence interval, NA: not applicable (as only 1 comparison), RCT: randomized controlled trial

**GRADE Working Group grades of evidence**
**High certainty:** We are very confident that the true effect lies close to that of the estimate of the effect
**Moderate certainty:** We are moderately confident in the effect estimate: The true effect is likely to be close to the estimate of the effect, but there is a possibility that it is substantially different
**Low certainty:** Our confidence in the effect estimate is limited: The true effect may be substantially different from the estimate of the effect
**Very low certainty:** We have very little confidence in the effect estimate: The true effect is likely to be substantially different from the estimate of effect

## GRADE Evidence table: Hematocrit (proportion by volume of red blood cells in blood)

| **Certainty assessment** | | | | | | | **Summary of findings** | | | | | **Importance** |
| --- | --- | --- | --- | --- | --- | --- | --- | --- | --- | --- | --- | --- |
|  |  |  |  |  |  |  | **No of patients** | | **Effect** | | **Certainty** |  |
| **No of studies** | **Design** | **Risk of bias** | **Inconsistency** | **Indirectness** | **Imprecision** | **Other considerations** | **Intervention** | **Control** | **Relative (95% CI)** | **Absolute (95% CI)** |  |  |
| **Hematocrit (proportion): by age** | | | | | | | | | | | | |
| *In children aged 0-90 days (4 mg/d), OVERALL* | | | | | | | | | | | | |
| 1 | NRCT | serious^1^ | NA | not serious | serious^2^ | none | 34 | 34 | - | 0.00  (-0.02, 0.03) | ⊕⊕⊝⊝  LOW^a^ | Critical |
| *In children aged 6–12 months (5–10 mg/d), OVERALL* | | | | | | | | | | | | |
| 2 | RCT | not serious | serious^3^ | serious^4^ | not serious | none | 616 | 601 | - | 0.01  (-0.01, 0.02) | ⊕⊕⊝⊝  LOW^b^ | Critical |
| *In children aged >12 months (10 mg/d), OVERALL* | | | | | | | | | | | | |
| 3 | RCT/ NRCT | serious^5^ | not serious | serious^6^ | serious^2^ | none | 99 | 94 | - | 0.00  (-0.01, 0.01) | ⊕⊝⊝⊝  VERY LOW^c^ | Critical |
| *In children aged 0–>12 months (5-10 mg/d), OVERALL* | | | | | | | | | | | | |
| 6 | RCT/ NRCT | serious^5^ | not serious | serious^7^ | not serious | none | 749 | 729 | - | 0.00  (-0.01, 0.01) | ⊕⊕⊝⊝  LOW^d^ | Critical |
| **Hematocrit (proportion): by treatment duration** | | | | | | | | | | | | |
| *In children receiving interventions for < 3 months (12.3 mg/d), OVERALL* | | | | | | | | | | | | |
| 1 | RCT | serious^1^ | NA | serious^8^ | serious^2^ | none | 48 | 40 |  | -0.01 (-0.04, 0.02) | ⊕⊝⊝⊝  VERY LOW^e^ | Critical |
| *In children receiving interventions for > 3 months (4–10 mg/d), OVERALL* | | | | | | | | | | | | |
| 5 | RCT/ NRCT | serious^5^ | not serious | serious^7^ | not serious | none | 701 | 689 | - | 0.00  (-0.01, 0.01) | ⊕⊕⊝⊝  LOW^f^ | Critical |
| **Hematocrit (proportion): by zinc form** | | | | | | | | | | | | |
| *In children receiving zinc as zinc gluconate (10 mg/d), OVERALL* | | | | | | | | | | | | |
| 1 | RCT | not serious | NA | serious^4^ | serious^2^ | none | 61 | 54 | - | 0.02  (0.00, 0.03) | ⊕⊕⊝⊝  LOW^g^ | Critical |
| *In children receiving zinc as zinc sulphate (4-12 mg/d), OVERALL* | | | | | | | | | | | | |
| 3 | RCT | serious^1^ | not serious | serious^9^ | serious^2^ | none | 112 | 102 | - | 0.00  (-0.01, 0.01) | ⊕⊝⊝⊝  VERY LOW^h^ | Critical |
| *In children receiving an unstated form of zinc (5–10 mg/d), OVERALL* | | | | | | | | | | | | |
| 1 | RCT | not serious | NA | serious^10^ | not serious | none | 555 | 547 | - | 0.00  (0.00, 0.00) | ⊕⊕⊕⊝  MODERATE^i^ | Critical |
| *In children receiving an ‘other’ form of zinc (4 mg/d), OVERALL* | | | | | | | | | | | | |
| 1 | RCT | serious^5^ | NA | serious^11^ | serious^2^ | none | 21 | 26 | - | -0.01  (-0.02, 0.01) | ⊕⊝⊝⊝  VERY LOW^j^ | Critical |
| **Hematocrit (proportion): zinc versus placebo** | | | | | | | | | | | | |
| *In children receiving zinc versus placebo (5-10 mg/d), OVERALL* | | | | | | | | | | | | |
| 2 | RCT | serious^5^ | not serious | serious^6^ | serious^2^ | none | 51 | 54 | - | 0.00  (-0.01, 0.01) | ⊕⊝⊝⊝  VERY LOW^c^ | Critical |

**Explanations**

^1^High risk of bias in randomization process (selection bias)

^2^Small sample size

^3^Large variation in point estimates, limited overlap in confidence intervals, I^2^≥75%

^4^Acute diarrhoea/gastroenteritis and underlying malnutrition

^5^High risk of bias in randomization process (selection bias) and deviations from intended interventions (performance bias)

^6^Underlying malnutrition and anemia, age range up to 6 years

**^7^**Acute diarrhoea/gastroenteritis, underlying malnutrition and anemia, age range up to 6 years

^8^Underlying anemia, age range up to 48 months

^9^Underlying malnutrition and anemia, age range up to 59 months

^10^Underlying malnutrition

^11^Underlying malnutrition and anemia

^a^Downgraded one level for study limitations (selection bias) and one level for imprecision (small sample size)

^b^Downgraded one level for inconsistency (large variation in point estimates, limited overlap in confidence intervals, I^2^≥75%) and one level for indirectness (acute diarrhoea/gastroenteritis and underlying malnutrition)

^c^Downgraded one level for study limitations (selection and performance bias), one level for indirectness (underlying malnutrition and anemia, age range up to 6 years) and one level for imprecision (small sample size)

^d^Downgraded one level for study limitations (selection and performance bias) and one level for indirectness (acute diarrhoea/gastroenteritis, underlying malnutrition and anemia, age range up to 6 years)

^e^Downgraded one level for study limitations (selection bias), one level for indirectness (underlying anemia, age range up to 48 months) and one level for imprecision (small sample size)

^f^Downgraded one level for study limitations (selection and performance bias) and one level for indirectness (acute diarrhoea/gastroenteritis, underlying malnutrition and anemia, age range up to 6 years)

^g^Downgraded one level for indirectness (acute diarrhoea/gastroenteritis and underlying malnutrition) and one level for imprecision (small sample size)

^h^Downgraded one level for study limitations (selection bias), one level for indirectness (underlying malnutrition and anemia, age range up to 59 months) and one level for imprecision (small sample size)

^i^Downgraded one level for indirectness (underlying malnutrition)

^j^Downgraded one level for study limitations (selection and performance bias), one level for indirectness (underlying malnutrition and anemia) and one level for imprecision (small sample size)

CI: confidence interval, NA: not applicable (as only 1 comparison), RCT: randomized controlled trial

**GRADE Working Group grades of evidence**
**High certainty:** We are very confident that the true effect lies close to that of the estimate of the effect
**Moderate certainty:** We are moderately confident in the effect estimate: The true effect is likely to be close to the estimate of the effect, but there is a possibility that it is substantially different
**Low certainty:** Our confidence in the effect estimate is limited: The true effect may be substantially different from the estimate of the effect
**Very low certainty:** We have very little confidence in the effect estimate: The true effect is likely to be substantially different from the estimate of effect

## GRADE Evidence table: Raised C-reactive protein

| **Certainty assessment** | | | | | | | **Summary of findings** | | | | | **Importance** |
| --- | --- | --- | --- | --- | --- | --- | --- | --- | --- | --- | --- | --- |
|  |  |  |  |  |  |  | **No of patients** | | **Effect** | | **Certainty** |  |
| **No of studies** | **Design** | **Risk of bias** | **Inconsistency** | **Indirectness** | **Imprecision** | **Other considerations** | **Intervention** | **Control** | **Relative**  **(95% CI)** | **Absolute** |  |  |
| **Raised CRP: by age** | | | | | | | | | | | | |
| *In children aged 6–12 months (5-10 mg/d)* | | | | | | | | | | | | |
| 2 (6 comparisons) | RCT | not serious | not serious | serious^1^ | serious^2^ | none | 68/377 | 56/302 | OR 1.08 (0.71, 1.65) | 18 per 100 in Zn vs 19 per 100 in control | ⊕⊕⊝⊝  LOW^a^ | Important |
| *In children aged > 12 months (10 mg/d)* | | | | | | | | | | | | |
| 1 (2 comparisons) | RCT | not serious | not serious | serious^3^ | serious^2^ | none | 104/297 | 90/301 | OR 1.26  (0.89, 1.78) | 35 per 100 in Zn vs 30 per 100 in control | ⊕⊕⊝⊝  LOW^b^ | Important |
| *In children aged 6–> 12 months, OVERALL* | | | | | | | | | | | | |
| 3 (8 comparisons) | RCT | not serious | not serious | serious^3^ | serious^2^ | none | 172/674 | 146/603 | OR 1.19 (0.91, 1.55) | 26 per 100 in Zn vs 24 per 100 in control | ⊕⊕⊝⊝  LOW^b^ | Important |
| **Raised CRP: by treatment duration** | | | | | | | | | | | | |
| *In children receiving interventions for > 3 months (5-10 mg/d), OVERALL* | | | | | | | | | | | | |
| 3 (8 comparisons) | RCT | not serious | not serious | serious^3^ | serious^2^ | none | 172/674 | 146/603 | OR 1.19 (0.91, 1.55) | 26 per 100 in Zn vs 24 per 100 in control | ⊕⊕⊝⊝  LOW^b^ | Important |
| **Raised CRP: by zinc form** | | | | | | | | | | | | |
| *In children receiving an unstated form of zinc (5-10 mg/d), OVERALL* | | | | | | | | | | | | |
| 3 (8 comparisons) | RCT | not serious | not serious | serious^3^ | serious^2^ | none | 172/674 | 146/603 | OR 1.19 (0.91, 1.55) | 26 per 100 in Zn vs 24 per 100 in control | ⊕⊕⊝⊝  LOW^b^ | Important |
| **Raised CRP: zinc versus placebo** | | | | | | | | | | | | |
| *In children receiving zinc versus placebo (5-10 mg/d), OVERALL* | | | | | | | | | | | | |
| 3 (4 comparisons) | RCT | not serious | not serious | serious^3^ | serious^2^ | none | 110/388 | 86/323 | OR 1.05 (0.74, 1.48) | 28 per 100 in Zn vs 27 per 100 in control | ⊕⊕⊝⊝  LOW^b^ | Important |
| **Raised CRP: high versus low dose zinc** | | | | | | | | | | | | |
| *In children receiving high versus low dose zinc (10 mg/d vs 5 mg/d), OVERALL* | | | | | | | | | | | | |
| 1 | RCT | not serious | NA | serious^1^ | serious^2^ | none | 23/79 | 27/75 | OR 0.73 (0.37, 1.44) | 29 per 100 in Zn vs 36 per 100 in control | ⊕⊕⊝⊝  LOW^a^ | Important |

**Explanations**

^1^Underlying malnutrition, anemia and malaria

^2^Wide confidence intervals including benefit and harm, small number of events

^3^Underlying malnutrition, anemia and malaria, age range up to 60 months

^a^Downgraded one level for indirectness (underlying malnutrition, anemia and malaria) and one level for imprecision (wide confidence intervals including benefit and harm, small number of events)

^b^Downgraded one level for indirectness (underlying malnutrition, anemia and malaria, age range up to 60 months) and one level for imprecision (wide confidence intervals including benefit and harm, small number of events)

CI: confidence interval, NA: not applicable (as only 1 comparison), RCT: randomized controlled trial, OR: odds ratio

**GRADE Working Group grades of evidence**
**High certainty:** We are very confident that the true effect lies close to that of the estimate of the effect
**Moderate certainty:** We are moderately confident in the effect estimate: The true effect is likely to be close to the estimate of the effect, but there is a possibility that it is substantially different
**Low certainty:** Our confidence in the effect estimate is limited: The true effect may be substantially different from the estimate of the effect
**Very low certainty:** We have very little confidence in the effect estimate: The true effect is likely to be substantially different from the estimate of effect

## GRADE Evidence table: Erythrocyte super oxide dismutase (IU/mg hemoglobin)

| **Certainty assessment** | | | | | | | **Summary of findings** | | | | | **Importance** |
| --- | --- | --- | --- | --- | --- | --- | --- | --- | --- | --- | --- | --- |
|  |  |  |  |  |  |  | **No of patients** | | **Effect** | | **Certainty** |  |
| **No of studies** | **Design** | **Risk of bias** | **Inconsistency** | **Indirectness** | **Imprecision** | **Other considerations** | **Intervention** | **Control** | **Relative**  **(95% CI)** | **Absolute** |  |  |
| **Erythrocyte super oxide dismutase: by dose** | | | | | | | | | | | | |
| *In children aged > 12 months (5-10 mg/d)* | | | | | | | | | | | | |
| 1 | RCT | not serious | NA | serious^1^ | serious^2^ | none | 53 | 49 | 0.25 IU/mg Hb (0.00, 0.50) | - | ⊕⊕⊝⊝  LOW^a^ | Critical |
| *In children aged > 12 months (bolus 140 mg/wk [70 mg twice weekly])* | | | | | | | | | | | | |
| 1 | NRCT | serious^3^ | NA | not serious | serious^4^ | none | 53 | 53 | 0.04 IU/mg Hb (-0.20, 0.28) | - | ⊕⊕⊝⊝  LOW^b^ | Critical |
| *In children aged > 12 months, OVERALL* | | | | | | | | | | | | |
| 2 | RCT/ NRCT | serious^3^ | not serious | serious^1^ | serious^4^ | none | 106 | 102 | 0.14 IU/mg Hb (-0.06, 0.35) | - | ⊕⊕⊝⊝  LOW | Critical |

**Explanations**

^1^Underlying malnutrition

^2^Wide confidence intervals including no effect, small sample size

^3^High risk of bias in randomization process (selection bias)

^4^Wide confidence intervals including benefit and harm, small sample size

^a^Downgraded one level for indirectness (underlying malnutrition) and one level for imprecision (wide confidence intervals including no effect, small sample size)

^b^Downgraded one level for study limitations (selection bias) and one level for imprecision (wide confidence intervals including benefit and harm, small sample size)

^c^Downgraded one level for study limitations (selection bias), one level for indirectness (underlying malnutrition) and one level for imprecision (wide confidence intervals including benefit and harm, small sample size)

CI: confidence interval, NA: not applicable (as only 1 comparison), NRCT: non-randomized controlled trial, RCT: randomized controlled trial

**GRADE Working Group grades of evidence**
**High certainty:** We are very confident that the true effect lies close to that of the estimate of the effect
**Moderate certainty:** We are moderately confident in the effect estimate: The true effect is likely to be close to the estimate of the effect, but there is a possibility that it is substantially different
**Low certainty:** Our confidence in the effect estimate is limited: The true effect may be substantially different from the estimate of the effect
**Very low certainty:** We have very little confidence in the effect estimate: The true effect is likely to be substantially different from the estimate of effect

## GRADE Evidence table: Zinc protoporphyrin (µmol/mol heme)

| **Certainty assessment** | | | | | | | **Summary of findings** | | | | | **Importance** |
| --- | --- | --- | --- | --- | --- | --- | --- | --- | --- | --- | --- | --- |
|  |  |  |  |  |  |  | **No of patients** | | **Effect** | | **Certainty** |  |
| **No of studies** | **Design** | **Risk of bias** | **Inconsistency** | **Indirectness** | **Imprecision** | **Other considerations** | **Intervention** | **Control** | **Relative**  **(95% CI)** | **Absolute** |  |  |
| **Zinc protoporphyrin** | | | | | | | | | | | | |
| *In children aged > 6-12 months (5-10 mg/d), OVERALL* | | | | | | | | | | | | |
| 2 (5 comparisons) | RCT | not serious | serious^1^ | serious^2^ | serious^3^ | none | 194 | 205 | -5.81 µmol/mol haeme  (-28.23, 16.61) | - | ⊕⊝⊝⊝  VERY LOW^a^ | Critical |

**Explanations**

^1^Large difference in point estimates, limited overlap in confidence intervals, I^2^≥50%

^2^Underlying malnutrition, anemia and malaria

^3^Wide confidence intervals including benefit and harm, small sample size

^a^Downgraded one level for inconsistency (large difference in point estimates, limited overlap in confidence intervals, I^2^≥50%), one level for indirectness (underlying malnutrition, anemia and malaria) and one level for imprecision (wide confidence intervals including benefit and harm, small sample size)

CI: confidence interval, RCT: randomized controlled trial

**GRADE Working Group grades of evidence**
**High certainty:** We are very confident that the true effect lies close to that of the estimate of the effect
**Moderate certainty:** We are moderately confident in the effect estimate: The true effect is likely to be close to the estimate of the effect, but there is a possibility that it is substantially different
**Low certainty:** Our confidence in the effect estimate is limited: The true effect may be substantially different from the estimate of the effect
**Very low certainty:** We have very little confidence in the effect estimate: The true effect is likely to be substantially different from the estimate of effect

## GRADE Evidence table: Serum total cholesterol (mg/dL)

| **Certainty assessment** | | | | | | | **Summary of findings** | | | | | **Importance** |
| --- | --- | --- | --- | --- | --- | --- | --- | --- | --- | --- | --- | --- |
|  |  |  |  |  |  |  | **No of patients** | | **Effect** | | **Certainty** |  |
| **No of studies** | **Design** | **Risk of bias** | **Inconsistency** | **Indirectness** | **Imprecision** | **Other considerations** | **Intervention** | **Control** | **Relative**  **(95% CI)** | **Absolute** |  |  |
| **Serum total cholesterol: by age** | | | | | | | | | | | | |
| *In children aged 0-90 days (4 mg/d)* | | | | | | | | | | | | |
| 1 | NRCT | serious^1^ | NA | not serious | serious^2^ | none | 34 | 34 | 1.60 mg/dL (-15.44, 18.64) | - | ⊕⊕⊝⊝  LOW^a^ | Important |
| *In children aged > 12 months (3.75-10 mg/d)* | | | | | | | | | | | | |
| 2 | RCT/ NRCT | serious^1^ | not serious | serious^3^ | serious^2^ | none | 86 | 74 | -1.07 mg/dL  (-6.82, 4.69) | - | ⊕⊝⊝⊝  VERY LOW^b^ | Important |
| *In children aged 0- > 12 months, OVERALL* | | | | | | | | | | | | |
| 3 | RCT/ NRCT | serious^1^ | not serious | serious^3^ | serious^2^ | none | 120 | 108 | -0.79 mg/dL  (-6.24, 4.66) | - | ⊕⊝⊝⊝  VERY LOW^b^ | Important |
| **Serum total cholesterol: by treatment duration** | | | | | | | | | | | | |
| *In children receiving zinc for > 3 months, OVERALL* | | | | | | | | | | | | |
| 3 | RCT/ NRCT | serious^1^ | not serious | serious^3^ | serious^2^ | none | 120 | 108 | -0.79 mg/dL  (-6.24, 4.66) | - | ⊕⊝⊝⊝  VERY LOW^b^ | Important |
| **Serum total cholesterol: by zinc form** | | | | | | | | | | | | |
| *In children receiving zinc sulphate (4-10 mg/d)* | | | | | | | | | | | | |
| 2 | RCT/ NRCT | serious^1^ | not serious | serious^4^ | serious^2^ | none | 87 | 83 | -1.73 mg/dL (-7.70, 4.23) | - | ⊕⊝⊝⊝  VERY LOW^c^ | Important |
| *In children receiving an ‘other’ form of zinc (zinc oxide, 3.75 mg/d)* | | | | | | | | | | | | |
| 1 | NRCT | serious^1^ | NA | serious^5^ | serious^2^ | none | 33 | 25 | 4.00 mg/dL  (-9.45, 17.45) | - | ⊕⊝⊝⊝  VERY LOW^d^ | Important |
| **Serum total cholesterol: zinc versus placebo** | | | | | | | | | | | | |
| *In children receiving zinc versus placebo (3.75-10 mg/d), OVERALL* | | | | | | | | | | | | |
| 2 | RCT/ NRCT | serious^1^ | not serious | serious^3^ | serious^2^ | none | 86 | 74 | -1.07 mg/dL  (-6.82, 4.69) | - | ⊕⊝⊝⊝  VERY LOW^b^ | Important |

**Explanations**

^1^High risk of bias in randomization process (selection bias)

^2^Wide confidence intervals including benefit and harm, small sample size

^3^Underlying malnutrition, age range up to 90 months

^4^Underlying malnutrition

^5^Age range up to 90 months

^a^Downgraded one level for study limitations (selection bias) and one level for imprecision (wide confidence intervals including benefit and harm, small sample size)

^b^Downgraded one level for study limitations (selection bias), one level for indirectness (underlying malnutrition, age range up to 90 months) and one level for imprecision (wide confidence intervals including benefit and harm, small sample size)

^c^Downgraded one level for study limitations (selection bias), one level for indirectness (underlying malnutrition) and one level for imprecision (wide confidence intervals including benefit and harm, small sample size)

^d^Downgraded one level for study limitations (selection bias), one level for indirectness (age range up to 90 months) and one level for imprecision (wide confidence intervals including benefit and harm, small sample size)

CI: confidence interval, NA: not applicable (as only 1 comparison), NRCT: non-randomized controlled trial, RCT: randomized controlled trial

**GRADE Working Group grades of evidence**
**High certainty:** We are very confident that the true effect lies close to that of the estimate of the effect
**Moderate certainty:** We are moderately confident in the effect estimate: The true effect is likely to be close to the estimate of the effect, but there is a possibility that it is substantially different
**Low certainty:** Our confidence in the effect estimate is limited: The true effect may be substantially different from the estimate of the effect
**Very low certainty:** We have very little confidence in the effect estimate: The true effect is likely to be substantially different from the estimate of effect

## GRADE Evidence table: Lactulose:mannitol (molar ratio)

| **Certainty assessment** | | | | | | | **Summary of findings** | | | | | **Importance** |
| --- | --- | --- | --- | --- | --- | --- | --- | --- | --- | --- | --- | --- |
|  |  |  |  |  |  |  | **No of patients** | | **Effect** | | **Certainty** |  |
| **No of studies** | **Design** | **Risk of bias** | **Inconsistency** | **Indirectness** | **Imprecision** | **Other considerations** | **Intervention** | **Control** | **Relative**  **(95% CI)** | **Absolute** |  |  |
| **Lactulose:mannitol (molar ratio): by age** | | | | | | | | | | | | |
| *In children aged > 12 months (20mg/d–140 mg/wk [70 mg bolus twice weekly]), OVERALL* | | | | | | | | | | | | |
| 2 | RCT/ NRCT | serious^1^ | not serious | serious^2^ | serious^3^ | none | 116 | 116 | -0.08  (-0.14, -0.02) | - | ⊕⊝⊝⊝  VERY LOW^a^ | Important |
| **Lactulose:mannitol (molar ratio): by treatment duration** | | | | | | | | | | | | |
| *In children receiving zinc for 0-3 months (20 mg/d)* | | | | | | | | | | | | |
| 1 | RCT | not serious | NA | serious^2^ | serious^3^ | none | 72 | 77 | -0.08  (-0.15, -0.01) | - | ⊕⊕⊝⊝  LOW^b^ | Important |
| *In children receiving zinc for > 3 months (140 mg/wk [70 mg bolus twice weekly)* | | | | | | | | | | | | |
| 1 | NRCT | serious^1^ | NA | not serious | serious^4^ | none | 44 | 39 | -0.10  (-0.27, 0.07) | - | ⊕⊕⊝⊝  LOW^c^ | Important |
| **Lactulose:mannitol (molar ratio): by zinc form** | | | | | | | | | | | | |
| *In children receiving zinc gluconate (140 mg/wk [70 mg bolus twice weekly)* | | | | | | | | | | | | |
| 1 | NRCT | serious^1^ | NA | not serious | serious^4^ | none | 44 | 39 | -0.10  (-0.27, 0.07) | - | ⊕⊕⊝⊝  LOW^c^ | Important |
| *In children receiving zinc acetate (20 mg/d)* | | | | | | | | | | | | |
| 1 | RCT | not serious | NA | serious^2^ | serious^3^ | none | 72 | 77 | -0.08  (-0.15, -0.01) | - | ⊕⊕⊝⊝  LOW^b^ | Important |
| **Lactulose:mannitol (molar ratio): zinc versus placebo** | | | | | | | | | | | | |
| *In children receiving zinc versus placebo (20mg/d–140 mg/wk [70 mg bolus twice weekly]), OVERALL* | | | | | | | | | | | | |
| 2 | RCT/ NRCT | serious^1^ | not serious | serious^2^ | serious^3^ | none | 116 | 116 | -0.08  (-0.14, -0.02) | - | ⊕⊝⊝⊝  VERY LOW^a^ | Important |

**Explanations**

^1^High risk of bias in randomization process (selection bias)

^2^Underlying malnutrition and environmental enteric dysfunction

^3^Small sample size

^4^Wide confidence intervals including benefit and harm, small sample size

^a^Downgraded one level for study limitations (selection bias), one level for indirectness (underlying malnutrition and environmental enteric dysfunction) and one level for imprecision (small sample size)

^b^Downgraded one level for indirectness (underlying malnutrition and environmental enteric dysfunction) and one level for imprecision (small sample size)

^c^Downgraded one level for study limitations (selection bias) and one level for imprecision (wide confidence intervals including benefit and harm, small sample size)

CI: confidence interval, NA: not applicable (as only 1 comparison), NRCT: non-randomized controlled trial, RCT: randomized controlled trial

**GRADE Working Group grades of evidence**
**High certainty:** We are very confident that the true effect lies close to that of the estimate of the effect
**Moderate certainty:** We are moderately confident in the effect estimate: The true effect is likely to be close to the estimate of the effect, but there is a possibility that it is substantially different
**Low certainty:** Our confidence in the effect estimate is limited: The true effect may be substantially different from the estimate of the effect
**Very low certainty:** We have very little confidence in the effect estimate: The true effect is likely to be substantially different from the estimate of effect

## GRADE Evidence table: Serum iron (µg/dL)

| **Certainty assessment** | | | | | | | **Summary of findings** | | | | | **Importance** |
| --- | --- | --- | --- | --- | --- | --- | --- | --- | --- | --- | --- | --- |
|  |  |  |  |  |  |  | **No of patients** | | **Effect** | | **Certainty** |  |
| **No of studies** | **Design** | **Risk of bias** | **Inconsistency** | **Indirectness** | **Imprecision** | **Other considerations** | **Intervention** | **Control** | **Relative**  **(95% CI)** | **Absolute** |  |  |
| **Serum iron: by age** | | | | | | | | | | | | |
| *In children aged > 12 months (10–12.3 mg/d), OVERALL* | | | | | | | | | | | | |
| 2 | RCT | serious^1^ | serious^2^ | serious^3^ | serious^4^ | none | 78 | 68 | 7.40 µg/dL  (-4.74, 19.54) | - | ⊕⊝⊝⊝  VERY LOW^a^ | Important |
| **Serum iron: by treatment duration** | | | | | | | | | | | | |
| *In children receiving zinc for 0–3 months (12.3 mg/d)* | | | | | | | | | | | | |
| 1 | RCT | serious^1^ | NA | serious^5^ | serious^4^ | none | 48 | 40 | -0.12 µg/dL  (-11.16, 10.92) | - | ⊕⊝⊝⊝  VERY LOW^b^ | Important |
| *In children receiving zinc for > 3 months (10 mg/d)* | | | | | | | | | | | | |
| 1 | RCT | serious^1^ | NA | serious^6^ | serious^7^ | none | 30 | 28 | 12.50 µg/dL  (10.37, 14.63) | - | ⊕⊝⊝⊝  VERY LOW^c^ | Important |
| **Serum iron: by zinc form** | | | | | | | | | | | | |
| *In children receiving zinc sulphate (10–12.3 mg/d)* | | | | | | | | | | | | |
| 2 | RCT | serious^1^ | serious^2^ | serious^3^ | serious^4^ | none | 78 | 68 | 7.40 µg/dL  (-4.74, 19.54) | - | ⊕⊝⊝⊝  VERY LOW^a^ | Important |
| **Serum iron: zinc versus placebo** | | | | | | | | | | | | |
| *In children receiving zinc versus placebo (10 mg/d)* | | | | | | | | | | | | |
| 1 | RCT | serious^1^ | NA | serious^6^ | serious^7^ | none | 30 | 28 | 12.50 µg/dL  (10.37, 14.63) | - | ⊕⊝⊝⊝  VERY LOW^c^ | Important |

**Explanations**

^1^High risk of bias in randomization process (selection bias)

^2^Large difference in point estimates, limited overlap in confidence intervals, I^2^≥75%

^3^Underlying malnutrition and anemia, age range up to 59 months

^4^Wide confidence intervals including benefit and harm, small sample size

^5^Underlying anemia, age range up to 48 months

^6^Underlying malnutrition, age range up to 59 months

^7^Small sample size

^a^Downgraded one level for study limitations (selection bias), one level for inconsistency (large difference in point estimates, limited overlap in confidence intervals, I^2^≥75%), one level for indirectness (underlying malnutrition and anemia, age range up to 59 months) and one level for imprecision (wide confidence intervals including benefit and harm, small sample size)

^b^Downgraded one level for study limitations (selection bias), one level for indirectness (underlying anemia, age range up to 48 months) and one level for imprecision (wide confidence intervals including benefit and harm, small sample size)

^c^Downgraded one level for study limitations (selection bias), one level for indirectness (underlying malnutrition, age range up to 59 months) and one level for imprecision (small sample size)

CI: confidence interval, NA: not applicable (as only 1 comparison), NRCT: non-randomized controlled trial, RCT: randomized controlled trial

**GRADE Working Group grades of evidence**
**High certainty:** We are very confident that the true effect lies close to that of the estimate of the effect
**Moderate certainty:** We are moderately confident in the effect estimate: The true effect is likely to be close to the estimate of the effect, but there is a possibility that it is substantially different
**Low certainty:** Our confidence in the effect estimate is limited: The true effect may be substantially different from the estimate of the effect
**Very low certainty:** We have very little confidence in the effect estimate: The true effect is likely to be substantially different from the estimate of effect

#

# Funnel plots to assess publication bias

## Hemoglobin (g/L)


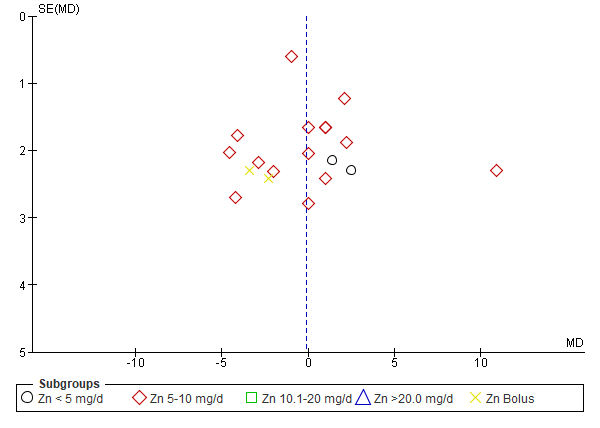


Figure 5 Hemoglobin (g/L) in children aged over 6 months to 12 months by zinc dose


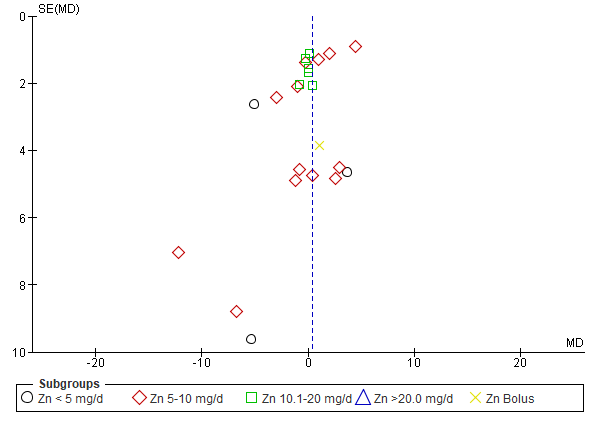


Figure 6 Hemoglobin (g/L) in children aged over 12 months by zinc dose


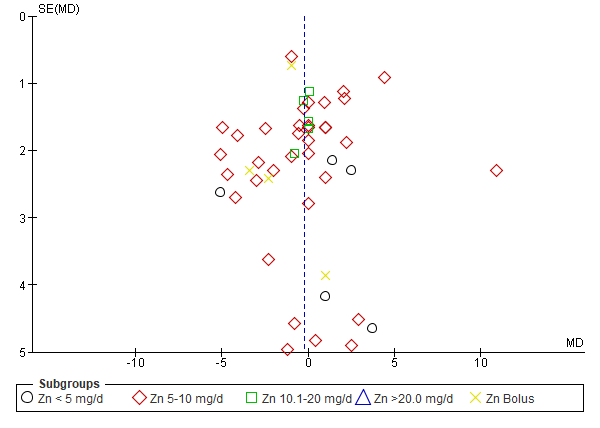


Figure 7 Hemoglobin (g/L) in children receiving interventions for >3 months by zinc dose


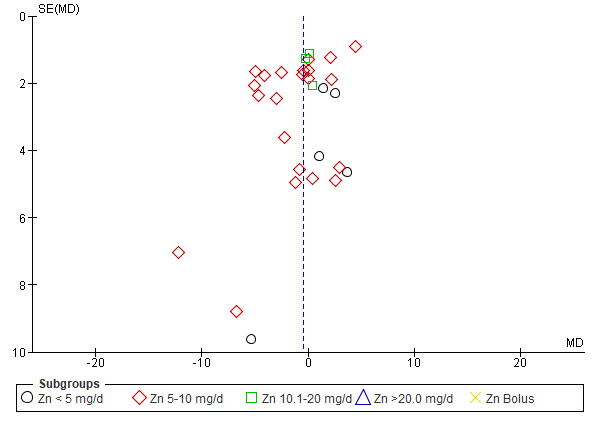


Figure 8 Hemoglobin (g/L) in children receiving zinc through zinc sulphate by zinc dose


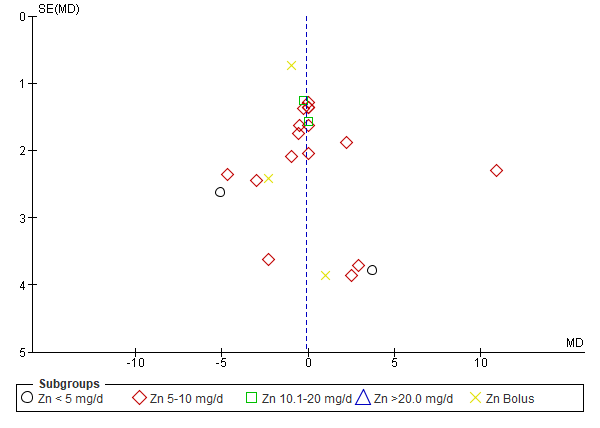


Figure 9 Hemoglobin (g/L) in children receiving zinc versus placebo by zinc dose

## Anemia (odds ratio)


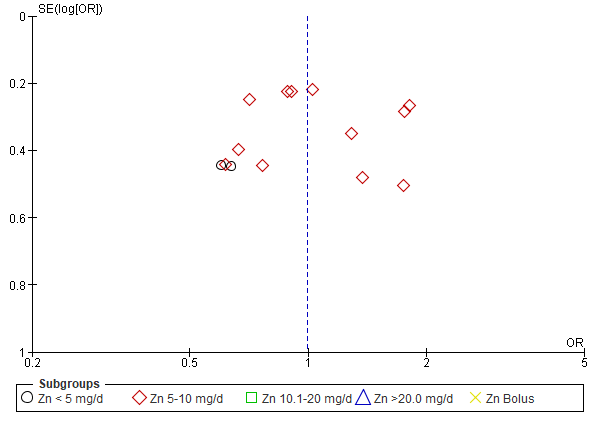


Figure 10 Anemia (odds ratio) in children aged over 6 months to 12 months by zinc dose


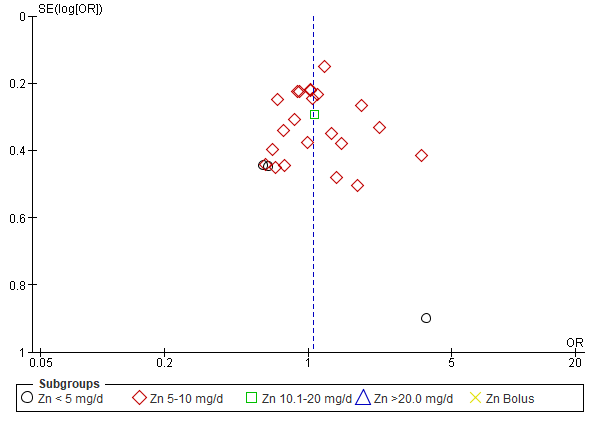


Figure 11 Anemia (odds ratio) in children receiving interventions for >3 months by zinc dose


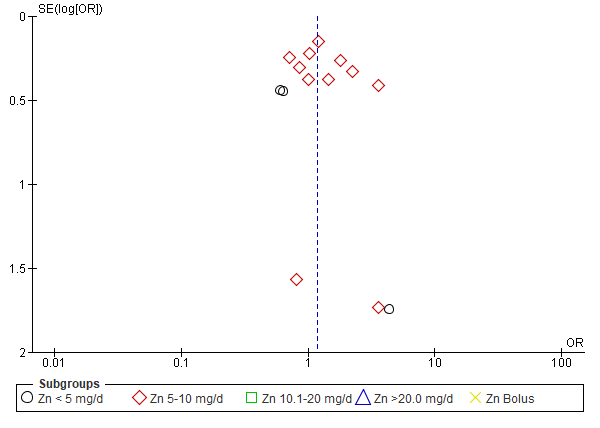


Figure 12 Anaemia (odds ratio) in children receiving zinc through zinc sulphate by zinc dose


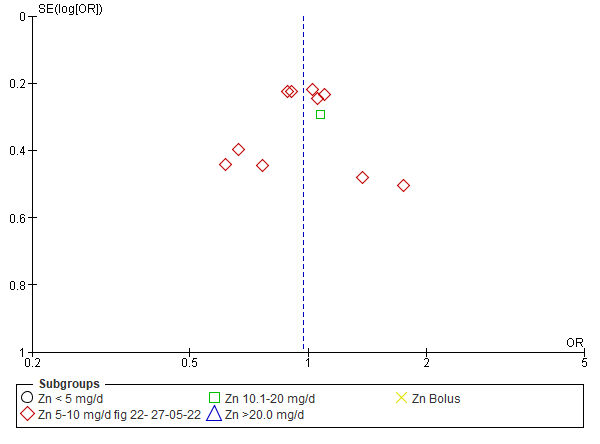


Figure 13 Anemia (odds ratio) in children receiving zinc through an unstated approach by zinc dose


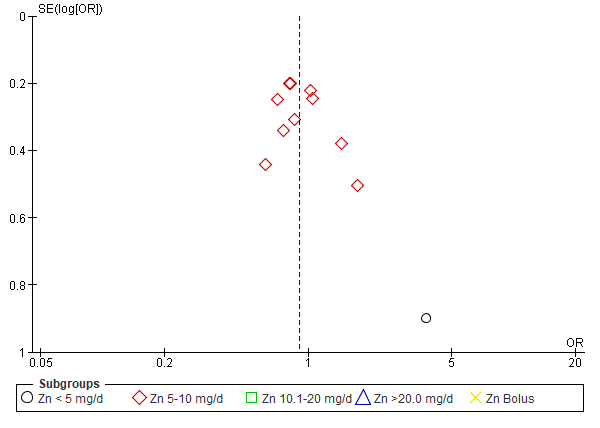


Figure 14 Anemia (odds ratio) in children receiving zinc versus placebo by zinc dose

## Ferritin


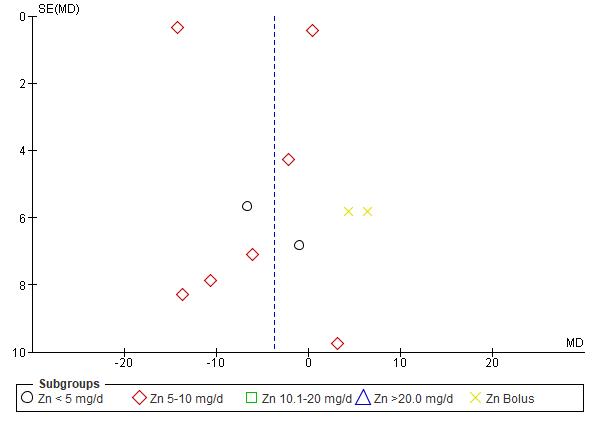


Figure 15Ferritin (µg/L) In children aged over 6 months to 12 months by zinc dose


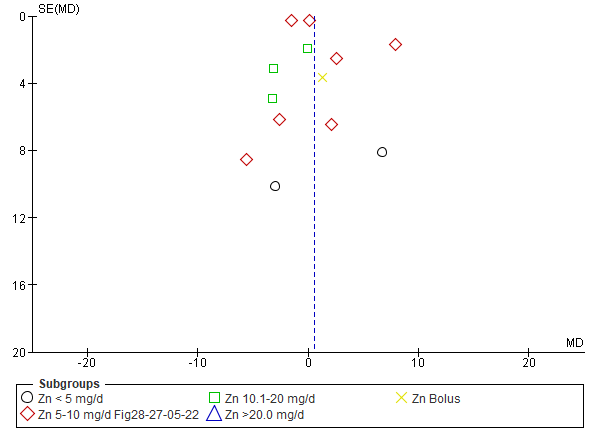


Figure 16 Ferritin in children aged over 12 months by zinc dose


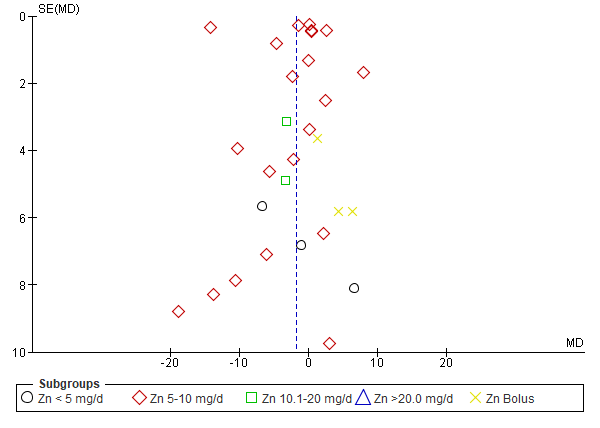


Figure 17 Ferritin (µg/L) in children receiving interventions for >3 months by zinc dose


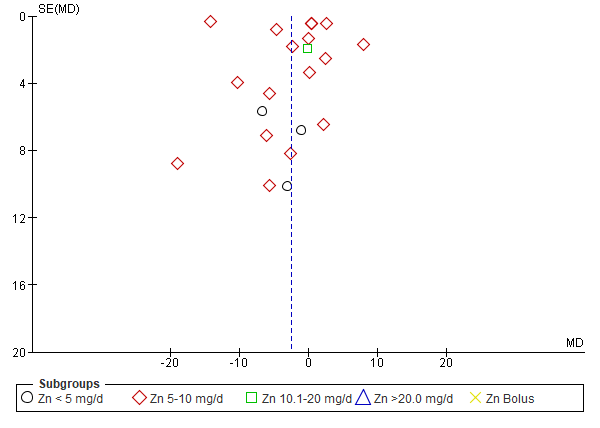


Figure 18 Ferritin (µg/L) in children receiving zinc through zinc sulphate by zinc dose


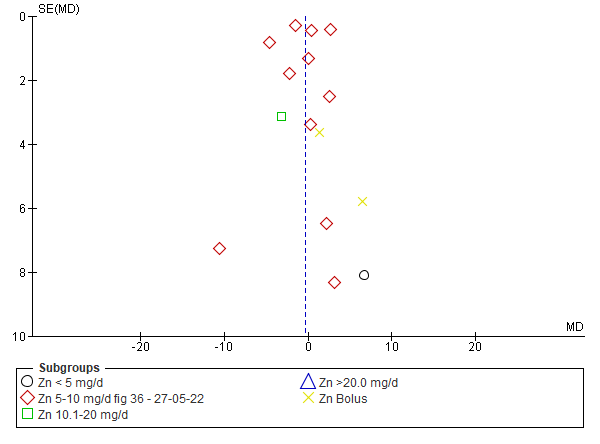


Figure 19 Ferritin (µg/L) in children receiving zinc versus placebo by zinc dose

## Serum/plasma copper (µg/dL)


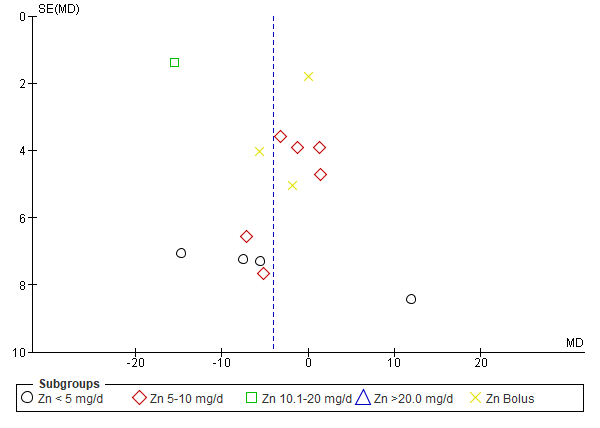


Figure 20 Serum/plasma copper (µg/dL) in children receiving interventions for >3 months by zinc dose


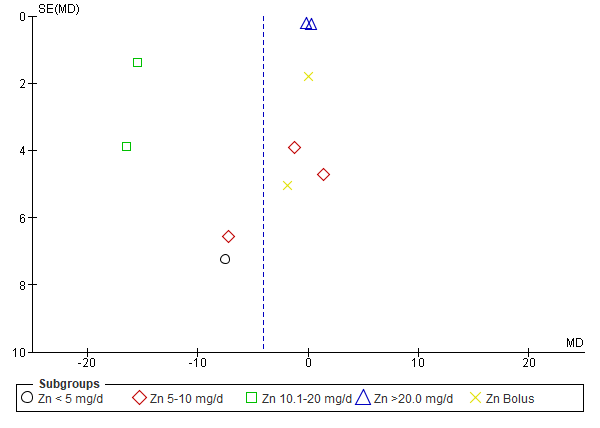


Figure 21 Serum/plasma copper (µg/dL) in children receiving zinc versus placebo by zinc dose

## Iron deficiency (odds ratio)


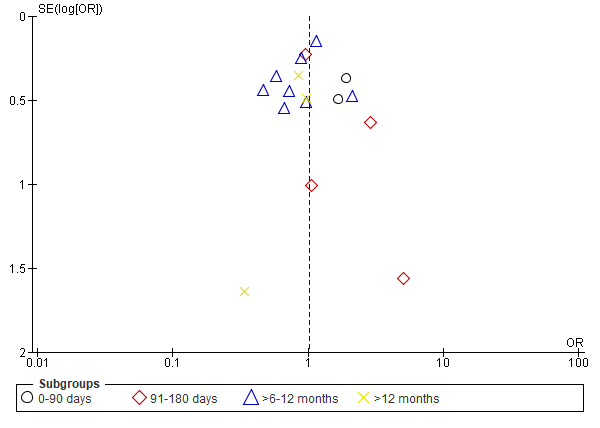


Figure 22 Iron deficiency (odds ratio) by age group


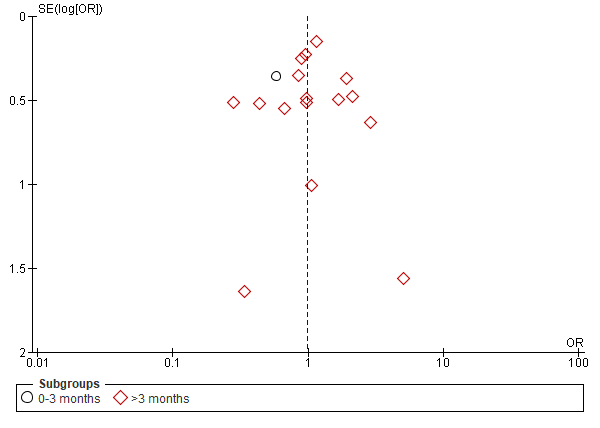


Figure 23 Iron deficiency (odds ratio) by treatment duration


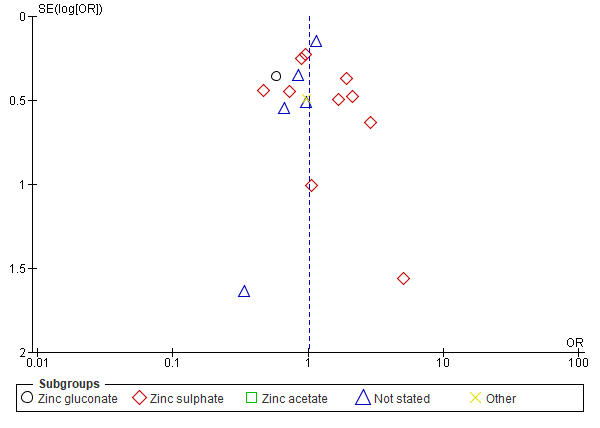


Figure 24 Iron deficiency (odds ratio) by delivery method

## Iron deficiency anemia (odds ratio)


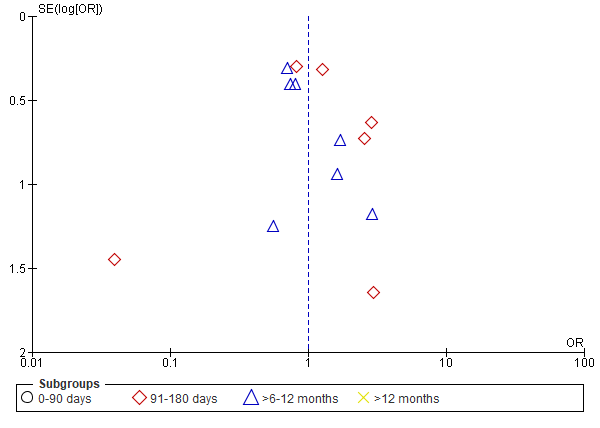


Figure 25 Iron deficiency anaemia (odds ratio) by age group


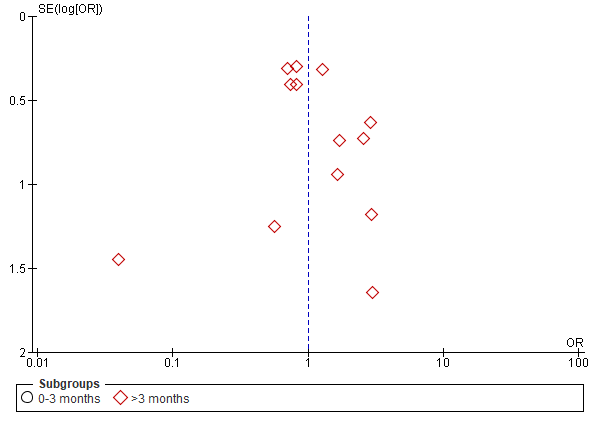


Figure 26 Iron deficiency anaemia (odds ratio) by duration of treatment


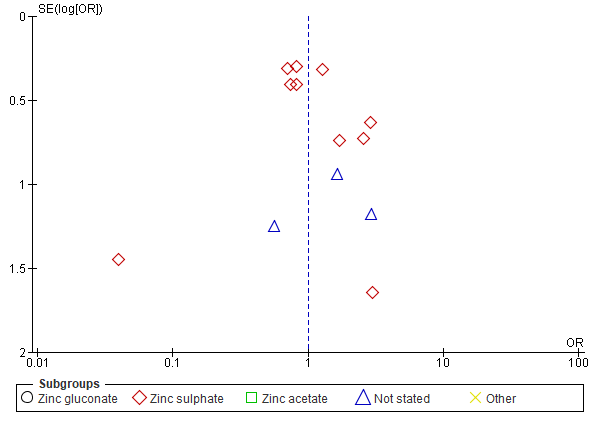


Figure 27 Iron deficiency anaemia (odds ratio) by delivery method

## Serum soluble transferrin receptor (sTfR) (mg/L)


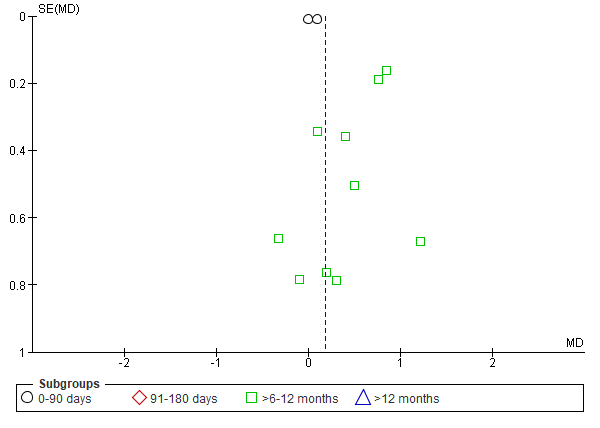


Figure 28 Serum soluble transferrin receptor (mg/L) by age group


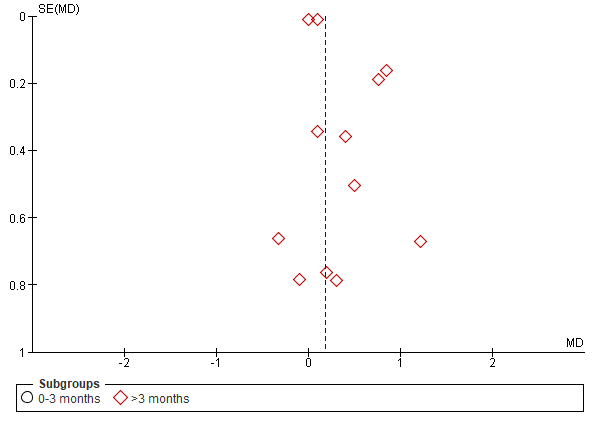


Figure 29 Serum/soluble transferrin receptor (mg/L) by treatment duration


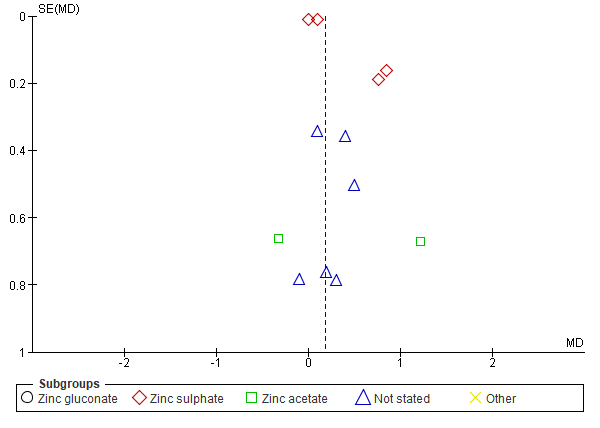


Figure 30 Serum/soluble transferrin receptor (mg/L) by delivery method

# Joanna Briggs Institute Critical Appraisal

## Methodological Assessment of Case reports

1. Were patient’s demographic characteristics clearly described?

2. Was the patient’s history clearly described and presented as a timeline?

3. Was the current clinical condition of the patient on presentation clearly described?

4. Were diagnostic tests or assessment methods and the results clearly described?

5. Was the intervention(s) or treatment procedure(s) clearly described?

6. Was the post-intervention clinical condition clearly described?

7. Were adverse events (harms) or unanticipated events identified and described?

8. Does the case report provide takeaway lessons?

**Note:** Reference numbers for JBI are per the reference list at the end of this supplementary file.

| Author and year | Question | | | | | | |  |  |
| --- | --- | --- | --- | --- | --- | --- | --- | --- | --- |
|  | 1 | 2 | 3 | 4 | 5 | 6 | 7 | 8 | Comments |
| Botash et al. (53) | Yes | Yes | Yes | Yes | Yes | Yes | Unclear | Yes |  |
| Sugiura et al. (54) | No | Yes | Yes | Yes | Yes | Yes | NA | Yes |  |

# References

1. Abbeddou S, Yakes Jimenez E, Somé JW, Ouédraogo JB, Brown. KH, Hess SY. Small-quantity lipid-based nutrient supplements containing different amounts of zinc along with diarrhea and malaria treatment increase iron and vitamin A status and reduce anemia prevalence, but do not affect zinc status in young Burkinabe children: A cl. BMC Pediatr [Internet]. 2017;17(1):1–17. Available from: http://dx.doi.org/10.1186/s12887-016-0765-9

2. Abdollahi M, Ajami M, Abdollahi Z, Kalantari N, Houshiarrad A, Fozouni F, et al. Zinc supplementation is an effective and feasible strategy to prevent growth retardation in 6 to 24 month children: A pragmatic double blind, randomized trial. Heliyon [Internet]. 2019 Nov;5(11):e02581. Available from: https://doi.org/10.1016/j.heliyon.2019.e02581

3. Alarcon K, Kolsteren PW, Prada AM, Chian AM, Velarde RE, Pecho IL, et al. Effects of separate delivery of zinc or zinc and vitamin A on hemoglobin response, growth, and diarrhea in young Peruvian children receiving iron therapy for anemia. Am J Clin Nutr. 2004;80(5):1276–82.

4. Baqui AH, Fischer Walker CL, Zaman K, El Arifeen S, Chowdhury HR, Wahed MA, et al. Weekly iron supplementation does not block increases in serum zinc due to weekly zinc supplementation in Bangladeshi infants. J Nutr. 2005;135(9):2187–91.

5. Bates CJ, Bates PH, Dardenne M, Prentice A, Lunn PG, Northrop-Clewes CA, et al. A trial of zinc supplementation in young rural Gambian children. Br J Nutr. 1993;69(1):243–55.

6. Berger J, Ninh NX, Khan NC, Nhien N V., Lien DK, Trung NQ, et al. Efficacy of combined iron and zinc supplementation on micronutrient status and growth in Vietnamese infants. Eur J Clin Nutr. 2006;60(4):443–54.

7. Bhandari N, Bahl R, Taneja S, Strand T, Molbak K, Ulvik RJ, et al. Substantial Reduction in Severe Diarrheal Morbidity by Daily Zinc Supplementation in Young North Indian Children. Pediatrics [Internet]. 2002 Jun 1;109(6):e86–e86. Available from: http://www.pediatrics.org/cgi/content/full/109/6/

8. Bhandari N, Taneja S, Mazumder S, Bahl R, Fontaine O, Bhan MK. Adding Zinc to Supplemental Iron and Folic Acid Does Not Affect Mortality and Severe Morbidity in Young Children. J Nutr [Internet]. 2007 Jan 1;137(1):112–7. Available from: https://academic.oup.com/jn/article/137/1/112/4664272

9. Bhutta ZA, Nizami SQ, Isani Z. Diarrhea in Pakistan. Pediatrics. 1999;103(4):1–9.

10. Brooks WA, Santosham M, Naheed A, Goswami D, Wahed MA, Diener-West M, et al. Effect of weekly zinc supplements on incidence of pneumonia and diarrhoea in children younger than 2 years in an urban, low-income population in Bangladesh: randomised controlled trial. Lancet [Internet]. 2005 Sep;366(9490):999–1004. Available from: https://ovidsp.ovid.com/ovidweb.cgi?T=JS&CSC=Y&NEWS=N&PAGE=fulltext&D=emed9&AN=41327324

11. Brown KH, De Romaña DL, Arsenault JE, Peerson JM, Penny ME. Comparison of the effects of zinc delivered in a fortified food or a liquid supplement on the growth, morbidity, and plasma zinc concentrations of young Peruvian children. Am J Clin Nutr. 2007;85(2):538–47.

12. Carter RC, Kupka R, Manji K, McDonald CM, Aboud S, Erhardt JG, et al. Zinc and multivitamin supplementation have contrasting effects on infant iron status: A randomized, double-blind, placebo-controlled clinical trial. Eur J Clin Nutr [Internet]. 2018 Jan 6;72(1):130–5. Available from: http://www.nature.com/articles/ejcn2017138

13. Caulfield LE, Zavaleta N, Chen P, Colombo J, Kannass K. Mineral status of non-anemic Peruvian infants taking an iron and copper syrup with or without zinc from 6 to 18 months of age: A randomized controlled trial. Nutrition [Internet]. 2013 Nov;29(11–12):1336–41. Available from: https://linkinghub.elsevier.com/retrieve/pii/S0899900713002815

14. Chang S, El Arifeen S, Bari S, Wahed MA, Rahman KM, Rahman MT, et al. Supplementing iron and zinc: Double blind, randomized evaluation of separate or combined delivery. Eur J Clin Nutr [Internet]. 2010 Feb 11;64(2):153–60. Available from: http://www.nature.com/articles/ejcn2009127

15. Chen L, Liu YF, Gong M, Jiang W, Fan Z, Qu P, et al. Effects of vitamin a, vitamin a plus zinc, and multiple micronutrients on anemia in preschool children in Chongqing, China. Asia Pac J Clin Nutr. 2012;21(1):3–11.

16. Chhagan MK, Van Den Broeck J, Luabeya KKA, Mpontshane N, Tomkins A, Bennish ML. Effect on longitudinal growth and anemia of zinc or multiple micronutrients added to vitamin A: A randomized controlled trial in children aged 6-24 months. BMC Public Health. 2010;10:1–11.

17. Dijkhuizen MA, Wieringa FT, West CE, Martuti S, Muhilal. Effects of iron and zinc supplementation in Indonesian infants on micronutrient status and growth. J Nutr. 2001;131(11):2860–5.

18. Fahmida U, Rumawas JSP, Utomo B, Patmonodewo S, Schultink W. Linear Growth of Stunted Infants With Low Haemoglobin. Asia Pac J Clin Nutr. 2007;16(October 2006):301–9.

19. Hambidge KM, Chavez MN, Brown RM, Walravens PA. Zinc nutritional status of young middle-income children and effects of consuming zinc-fortified breakfast cereals. Am J Clin Nutr. 1979;32(12):2532–9.

20. Hess SY, Abbeddou S, Jimenez EY, Somé JW, Vosti SA, Ouédraogo ZP, et al. Small-Quantity Lipid-Based Nutrient Supplements, Regardless of Their Zinc Content, Increase Growth and Reduce the Prevalence of Stunting and Wasting in Young Burkinabe Children: A Cluster-Randomized Trial. PLoS One. 2015;10(3):e0122242.

21. Lind T, Persson LÅ, Lönnerdal B, Stenlund H, Hernell O. Effects of weaning cereals with different phytate content on growth, development and morbidity: A randomized intervention trial in infants from 6 to 12 months of age. Acta Paediatr Int J Paediatr. 2004;93(12):1575–82.

22. López De Romaña D, Salazar M, Hambidge KM, Penny ME, Peerson JM, Krebs NF, et al. Longitudinal measurements of zinc absorption in Peruvian children consuming wheat products fortified with iron only or iron and 1 of 2 amounts of zinc. Am J Clin Nutr. 2005;81(3):637–47.

23. Mendoza NJ, Del Y, Peña CB, Frank Papalé-Centofanti J, Torres-Villanueva M, Castro M. Anthropometric and biochemical nutritional status, parasitic infestation, social stratification. The effects of zinc supplementation in children of Venezuelan public kindergartens. Rev Esp Nutr Comunitaria. 2016;22(2).

24. Moradveisi B, Yazdanifard P, Naleini N, Sohrabi M. Comparison of iron alone and zinc plus iron supplementation effect on the clinical and laboratory features of children with iron deficiency anemia. Int J Hematol Stem Cell Res. 2019;13(4):220–8.

25. Olney DK, Pollitt E, Kariger PK, Khalfan SS, Ali NS, Tielsch JM, et al. Combined iron and folic acid supplementation with or without zinc reduces time to walking unassisted among Zanzibari infants 5- to 11-mo old. J Nutr. 2006;136(9):2427–34.

26. Ouédraogo HZ, Dramaix-Wilmet M, Zeba AN, Hennart P, Donnen P. Effect of iron or multiple micronutrient supplements on the prevalence of anaemia among anaemic young children of a malaria-endemic area: A randomized double-blind trial. Trop Med Int Heal. 2008;13(10):1257–66.

27. Owusu-Agyei S, Newton S, Mahama E, Febir LG, Ali M, Adjei K, et al. Impact of vitamin A with zinc supplementation on malaria morbidity in Ghana. Nutr J. 2013;12(1):1–9.

28. Radhakrishna K V, Hemalatha R, Geddam JJB, Kumar PA, Balakrishna N, Shatrugna V. Effectiveness of zinc supplementation to full term normal infants: a community based double blind, randomized, controlled, clinical trial. PLoS One [Internet]. 2013;8(5):e61486. Available from: http://www.ncbi.nlm.nih.gov/pubmed/23737940

29. Richard SA, Zavaleta N, Caulfield LE, Black RE, Witzig RS, Shankar AH. Zinc and iron supplementation and malaria, diarrhea, and respiratory infections in children in the Peruvian Amazon. Am J Trop Med Hyg. 2006;75(1):126–32.

30. Rosado JL, López P, Muñoz E, Martinez H, Allen LH. Zinc supplementation reduced morbidity, but neither zinc nor iron supplementation affected growth or body composition of Mexican preschoolers. Am J Clin Nutr. 1997;65(1):13–9.

31. Ryan KN, Stephenson KB, Trehan I, Shulman RJ, Thakwalakwa C, Murray E, et al. Zinc or albendazole attenuates the progression of environmental enteropathy: A randomized controlled trial. Clin Gastroenterol Hepatol [Internet]. 2014;12(9):1–8. Available from: http://dx.doi.org/10.1016/j.cgh.2014.01.024

32. Sazawal S, Malik P, Jalla S, Krebs N, Bhan M, Black R. Zinc supplementation for four months does not affect plasma copper concentration in infants. Acta Paediatr [Internet]. 2004 May;93(5):599–602. Available from: https://ovidsp.ovid.com/ovidweb.cgi?T=JS&CSC=Y&NEWS=N&PAGE=fulltext&D=med5&AN=15174779

33. Sazawal S, Black RE, Ramsan M, Chwaya HM, Stoltzfus RJ, Dutta A, et al. Effects of routine prophylactic supplementation with iron and folic acid on admission to hospital and mortality in. Lancet [Internet]. 2006;367(9505):133–43. Available from: http://www.sciencedirect.com/science?_ob=GatewayURL&_origin=ScienceSearch&_method=citationSearch&_piikey=S0140673606679622&_version=1&_returnURL=&md5=59b88b4fa0756164e14fc63cc3316c5c

34. Shankar AH, Genton B, Baisor M, Jaino P, Tamja S, Adiguma T, et al. The influence of zinc supplementation on morbidity due to Plasmodium falciparum: A randomized trial in preschool children in Papua New Guinea. Am J Trop Med Hyg. 2000;62(6):663–9.

35. Silva APR, Vitolo MR, Zara LF, Castro CFS. Effects of zinc supplementation on 1- to 5-year old children. J Pediatr (Rio J). 2006;82(3):227–31.

36. Strand TA, Chandyo RK, Bahl R, Sharma PR, Adhikari RK, Bhandari N, et al. Effectiveness and Efficacy of Zinc for the Treatment of Acute Diarrhea in Young Children. Pediatrics [Internet]. 2002 May 1;109(5):898–903. Available from: http://pediatrics.aappublications.org/cgi/doi/10.1542/peds.109.5.898

37. Veenemans J, Milligan P, Prentice AM, Schouten LRA, Inja N, van der Heijden AC, et al. Effect of Supplementation with Zinc and Other Micronutrients on Malaria in Tanzanian Children: A Randomised Trial. von Seidlein L, editor. PLoS Med [Internet]. 2011 Nov 22;8(11):e1001125. Available from: https://dx.plos.org/10.1371/journal.pmed.1001125

38. Walravens PA, Hambidge KM. Growth of infants fed a zinc supplemented formula. Am J Clin Nutr. 1976;29(10):1114–21.

39. Wasantwisut E, Winichagoon P, Chitchumroonchokchai C, Yamborisut U, Boonpraderm A, Pongcharoen T, et al. Iron and Zinc Supplementation Improved Iron and Zinc Status, but Not Physical Growth, of Apparently Healthy, Breast-Fed Infants in Rural Communities of Northeast Thailand. J Nutr [Internet]. 2006 Sep 1;136(9):2405–11. Available from: https://academic.oup.com/jn/article/136/9/2405/4664952

40. Wuehler SE, Sempértegui F, Brown KH. Dose-response trial of prophylactic zinc supplements, with or without copper, in young Ecuadorian children at risk of zinc deficiency. Am J Clin Nutr [Internet]. 2008 Mar 1;87(3):723–33. Available from: https://academic.oup.com/ajcn/article/87/3/723/4633435

41. Zlotkin S, Arthur P, Schauer C, Antwi KY, Yeung G, Piekarz A. Home-Fortification with Iron and Zinc Sprinkles or Iron Sprinkles Alone Successfully Treats Anemia in Infants and Young Children. J Nutr [Internet]. 2003 Apr 1;133(4):1075–80. Available from: https://academic.oup.com/jn/article/133/4/1075/4688097

42. Awasthi S. Zinc Supplementation in Acute Diarrhea is Acceptable, Does Not Interfere with Oral Rehydration, and Reduces the Use of Other Medications. J Pediatr Gastroenterol Nutr [Internet]. 2006 Mar;42(3):300–5. Available from: http://journals.lww.com/jpgn

43. Fallah R, Sabbaghzadegan S, Karbasi SA, Binesh F. Efficacy of zinc sulfate supplement on febrile seizure recurrence prevention in children with normal serum zinc level: A randomised clinical trial. Nutrition. 2015 Nov 1;31(11–12):1358–61.

44. Gupta DN, Mondal SK, Ghosh S, Rajendran K, Sur D, Manna B. Impact of zinc supplementation on diarrhoeal morbidity in rural children of West Bengal, India. Acta Paediatr Int J Paediatr. 2003 May 1;92(5):531–6.

45. Kurugöl Z, Akilli M, Bayram N, Koturoglu G. The prophylactic and therapeutic effectiveness of zinc sulphate on common cold in children. Acta Paediatr [Internet]. 2006 Oct 1;95(10):1175–81. Available from: http://doi.wiley.com/10.1080/08035250600603024

46. Kurugöl Z, Bayram N, Atik T. Effect of zinc sulfate on common cold in children: Randomized, double blind study. Pediatr Int. 2007;49(6):842–7.

47. Larson CP, Hoque ABMM, Larson CP, Khan AM, Saha UR. Initiation of zinc treatment for acute childhood diarrhoea and risk for vomiting or regurgitation: A randomized, double-blind, placebo-controlled trial. J Heal Popul Nutr. 2005;23(4):311–9.

48. Malik A, Taneja DK, Devasenapathy N, Rajeshwari K. Zinc supplementation for prevention of acute respiratory infections in infants: A randomized controlled trial. Indian Pediatr. 2014;51(10):780–4.

49. Martinez-Estevez NS, Alvarez-Guevara AN, Rodriguez-Martinez CE. Effects of zinc supplementation in the prevention of respiratory tract infections and diarrheal disease in Colombian children: A 12-month randomised controlled trial. Allergol Immunopathol (Madr) [Internet]. 2016 Jul 1;44(4):368–75. Available from: https://linkinghub.elsevier.com/retrieve/pii/S0301054616300192

50. Passariello A, Nocerino R, Terrin G, Cecere G, De Marco G, Micillo M, et al. Acceptability and efficacy of a gel hypotonic oral rehydration solution in children with acute gastroenteritis. Eur J Gastroenterol Hepatol [Internet]. 2015 May 14;27(5):523–6. Available from: https://journals.lww.com/00042737-201505000-00007

51. Sampaio DLB, de Mattos ÂP, Ribeiro TCM, Leite ME de Q, Cole CR, Costa-Ribeiro H. Zinc and other micronutrients supplementation through the use of sprinkles: impact on the occurrence of diarrhea and respiratory infections in institutionalized children. J Pediatr (Rio J) [Internet]. 2013 May;89(3):286–93. Available from: https://www.cochranelibrary.com/central/doi/10.1002/central/CN-00959582/full

52. Valentiner-Branth P, Shrestha PS, Chandyo RK, Mathisen M, Basnet S, Bhandari N, et al. A randomized controlled trial of the effect of zinc as adjuvant therapy in children 2-35 mo of age with severe or nonsevere pneumonia in Bhaktapur, Nepal. Am J Clin Nutr. 2010 Jun 1;91(6):1667–74.

53. Botash AS, Nasca J, Dubowy R, Weinberger HL, Oliphant M. Zinc-Induced Copper Deficiency in an Infant. Arch Pediatr Adolesc Med [Internet]. 1992 Jun 1;146(6):709. Available from: https://ovidsp.ovid.com/ovidweb.cgi?T=JS&CSC=Y&NEWS=N&PAGE=fulltext&D=med3&AN=1595625

54. Sugiura T, Goto K, Ito K, Ueta A, Fujimoto S, Togari H. Chronic zinc toxicity in an infant who received zinc therapy for atopic dermatitis. Acta Paediatr [Internet]. 2005 Sep 5;94(9):1333–5. Available from: https://ovidsp.ovid.com/ovidweb.cgi?T=JS&CSC=Y&NEWS=N&PAGE=fulltext&D=med6&AN=16203677
